# *Ghost Neuron* preparation unlocks high-resolution structural mapping of intracellular architecture in axons

**DOI:** 10.1038/s41467-026-77741-4

**Published:** 2026-09-23

**Authors:** Satish Bodakuntla, Julia Marelli, Victor Vasquez-Montes, Simon Glauser, Christian Biertümpfel, Naoko Mizuno

**Affiliations:** 1 Laboratory of Structural Cell Biology, National Heart, Lung, and Blood Institute, National Institutes of Health, Bethesda, MD USA; 2 Eidgenössische Technische Hochschule Zürich, Department of Chemistry and Applied Biosciences, Institute of Pharmaceutical Sciences, Zürich, Switzerland; 3 National Institute of Arthritis and Musculoskeletal and Skin Diseases, National Institutes of Health, Bethesda, MD USA

**Keywords:** Cryoelectron microscopy, Electron microscopy

## Abstract

Cellular cryo-electron microscopy (cryo-EM) offers powerful tools to reveal molecular architecture inside cells, but dense cytoplasm often obscures fine molecular features, especially in narrow neuronal projections. Here, we introduce the *Ghost Neuron* technique, a preparation combining brief hypotonic treatment with controlled mechanical rupture immediately before freezing, facilitating high-contrast imaging of neuronal ultrastructure within its neurite topology. By removing the plasma membrane and reducing cytoplasmic density, the method allows high-resolution structural analysis while preserving the overall geometry of neurites. The method allows structural information to be mapped back onto neuronal architecture. The preparation also releases endogeneous macromolecular complexes onto EM grids, creating particle-rich regions suitable for high-resolution structural analysis. Using *Ghost Neuron*, we resolved axonal microtubules at 3.59 Å and 3.9 Å resolution, distinguishing expanded and compact lattice states, and mapped these states as interspersed patches along individual microtubules within axons. We also resolved axonal actin filaments and identified cofilin-decorated actin. These results establish *Ghost Neuron* as a discovery platform for linking molecular structure to neuronal organization.

## Abstract (web summary)

The authors introduce Ghost Neuron, a cryo-EM preparation that improves visualization of neuronal projections and maps macromolecular structures within axons at near-atomic resolution.

## Introduction

In situ cryogenic electron microscopy (cryo-EM) and electron tomography (cryo-ET) are techniques that facilitate the direct visualization of cellular architecture and organelles. This powerful imaging technique has led to unexpected discoveries of biological macromolecular assemblies underlying complex cellular functions^1–4^, revealing molecular organization and cellular behaviors directly within their native environment. However, in situ cryo-EM/cryo-ET remains technically limited to relatively small or thin biological specimens, such as bacteria^5^, yeast^6^, or peripheral regions of larger eukaryotic cells^7^. In contrast, for larger and thicker samples, cryo-focused ion beam (cryo-FIB) milling combined with correlative light and electron microscopy (CLEM) has been developed to access regions deep within cells^8^. Cryo-FIB produces thin lamellae suitable for cryo-EM and allows visualization of structures otherwise inaccessible in intact cells. Using this technique, molecular features such as the nuclear envelope, nucleosomes^9–11^, cytoplasmic granules^2^, and cytoskeleton elements^12,13^ have been successfully resolved. In addition, tissue and larger samples can be analyzed by cryo-FIB lift-out and subsequent lamella preparation^14,15^. Alternatively, isolated organelles, such as mitochondria^16,17^ or nuclei^18^, can be extracted for detailed structural characterization by single-particle analysis (SPA). While this isolation approach facilitates high-resolution analysis, it inevitably sacrifices the larger cellular context and may obscure critical interactions with surrounding structural or functional components.

Despite these advances, thin neuronal projections such as neurites remain difficult targets for high-resolution structural analysis. Although neurites are within the geometric thickness limits of cryo-EM and cryo-ET, their dense cytoplasm and the abundance of membrane-bound organelles substantially reduce image contrast, limiting alignment accuracy and ultimately achievable resolution. Several studies have imaged neuronal processes by cryo-EM and cryo-ET^19–25^, showing that neurites are accessible to cryogenic imaging, while also revealing persistent challenges in obtaining information at a molecular resolution due to poor contrast and cytoplasmic denseness. To analyze cellular components within membrane-rich regions, several specialized membrane-removal strategies have been developed. Cell unroofing mechanically removes the dorsal surface of adherent cells, thereby exposing the cytoplasmic face of the plasma membrane that remains attached to a solid substrate^26,27^. This approach allows for direct imaging of membrane-associated cytoskeletal and signaling structures. Similarly, ghost cell formation was originally described as a degenerative process in red blood cells (RBCs) that have lost hemoglobin and later adapted as a specialized preparation to visualize membrane architecture^28,29^. Both techniques utilize physical methods such as shear force to disrupt membranes while retaining much of the remaining cellular architecture, making them particularly amenable to investigation by classical EM approaches. Related surfactant-based extraction methods have provided informative access to cytoskeletal organization, including neuronal processes^30–32^. However, these methods are largely limited by the usage of chemical fixation and metal coating and, therefore, incompatible with high-resolution cryo-EM in a frozen-hydrated state.

Here, we introduce the *Ghost Neuron* approach, which selectively removes the plasma membrane from neurites while preserving overall neurite geometry. By controlled membrane rupture immediately before vitrification, the method facilitates high-resolution cryo-EM analysis of intracellular structures along neurite trajectories. Importantly, the preserved geometry allows structural information to be mapped back onto neuronal topology, while the dispersed material on the grid provides an additional source for single-particle analysis of endogenous assemblies. Using the *Ghost Neuron* approach, we obtained high-resolution structures of axonal microtubules, resolving coexisting expanded and compact lattice conformations within axons, as well as actin filaments with a sub-population of fully cofilin-decorated actin filaments. By back-plotting classified segments onto cryo-EM montages, we mapped microtubule conformational states and cytoskeletal architectures along individual neurites, revealing patches of expanded lattice within the shafts of axonal microtubules.

## Results

### Establishment of  the *Ghost Neuron* approach

**Fig. 1:**
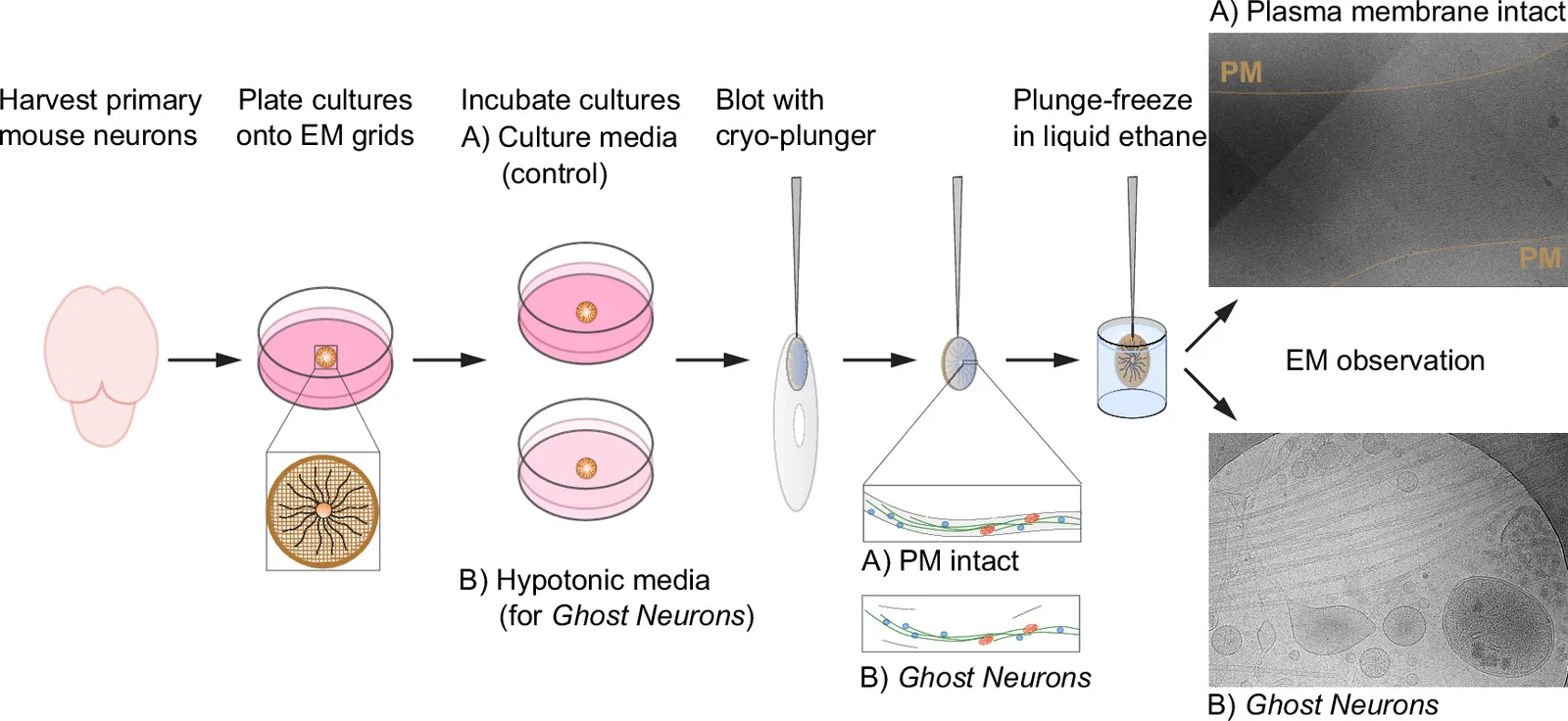
Schematic representation of the *Ghost Neuron* preparation. Primary thalamus tissue are dissected from embryo brains, cultured on coated EM grids, incubated with hypotoic media, blotted, and flash frozen in liquid ethane with a cryo-plunger. *Ghost Neurons* lose their plasma membrane (PM) upon vitrification whereas control cells stay intact.

*Ghost Neurons* were generated by exposing cultured neurons on EM grids to hypotonic media to increase membrane tension, followed by mechanical disruption by blotting the grid with filter paper and immediate vitrification (Fig. 1). During the incubation, the plasma membrane remains intact; rupture occurs only upon blotting immediately before freezing. This combined procedure selectively ruptures the plasma membrane while leaving the underlying cytoskeletal architecture and overall neurite geometry largely intact. As a result, the outer membrane barrier is removed and the cytoplasmic density is reduced, producing thin, high-contrast traces of neurites suitable for cryo-EM and cryo-ET imaging, and downstream high-resolution structural analysis.

### Mild hypotonic conditions preserve axonal geometrical integrity and mitochondrial transport

**Fig. 2:**
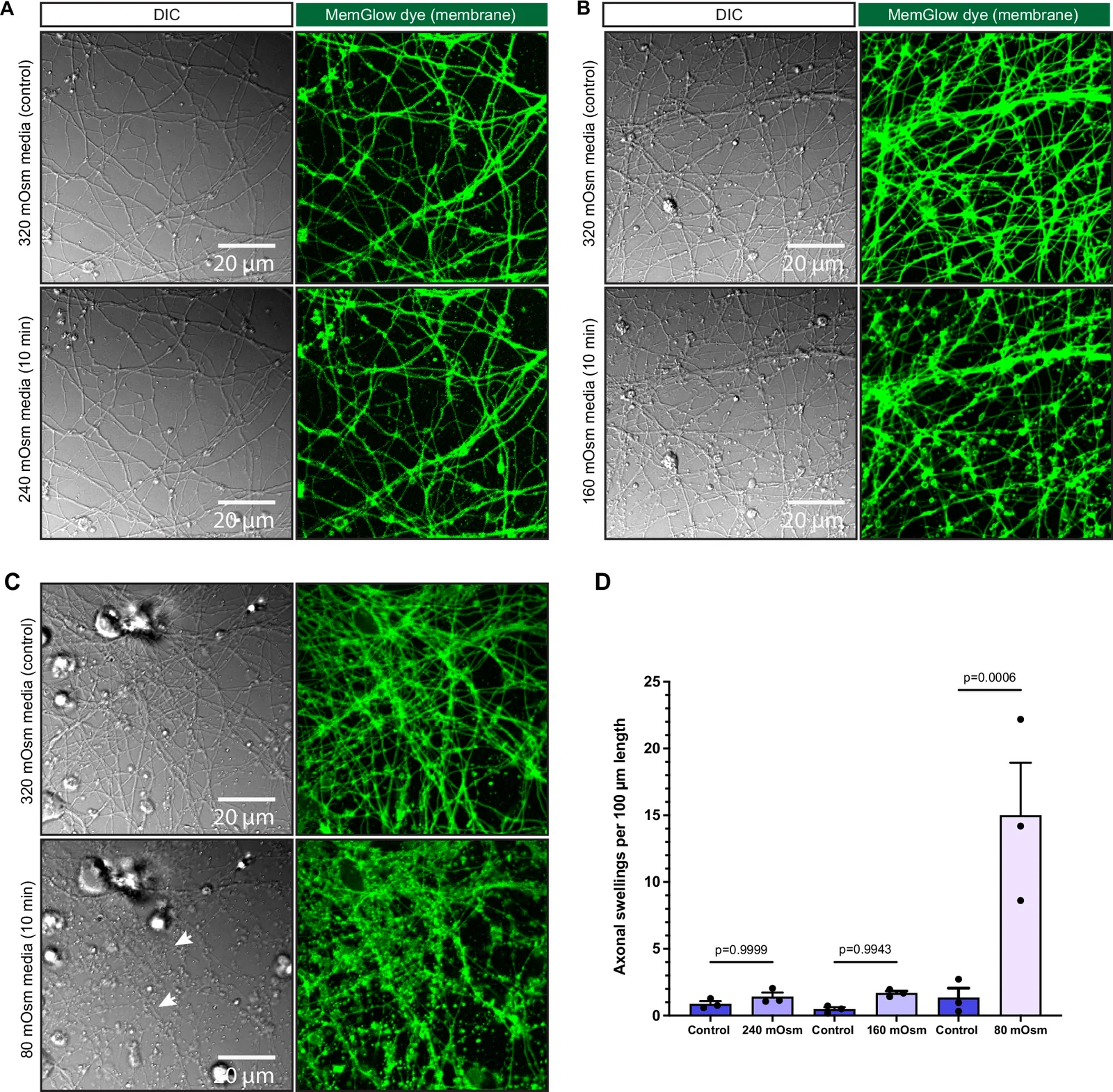
Effect of hypotonic treatment on the overall morphology of neurites. Live axons exposed to different hypotonic media were stained with MemGlow (green). **A**, **B** Neurons exposed to 240 mOsm and 160 mOsm media maintain overall morphology throughout the 10-min imaging period. **C** Neurons exposed to 80 mOsm media exhibit small membrane swellings. **D** Axonal swellings per 100 µm length are quantified and shown as bar graphs (mean ± SEM) overlaid with individual data points. Each data point represents the average axonal swellings per 100 µm in an individual experiment. *N* = 3 independent biological experiments. *p* values indicate comparisons to the Control group: 240 mOsm (*p* = 0.9999), 160 mOsm (*p* = 0.9943), and 80 mOsm (*p *= 0.0006). Significance was evaluated using one-way ANOVA with Holm-Šídák’s multiple comparisons test, degree of freedom is 12. Control conditions are at 320 mOsm.The corresponding video for this experiment is shown in Supplementary Movie 1. Source data are provided as a Source Data file.

**Fig. 3:**
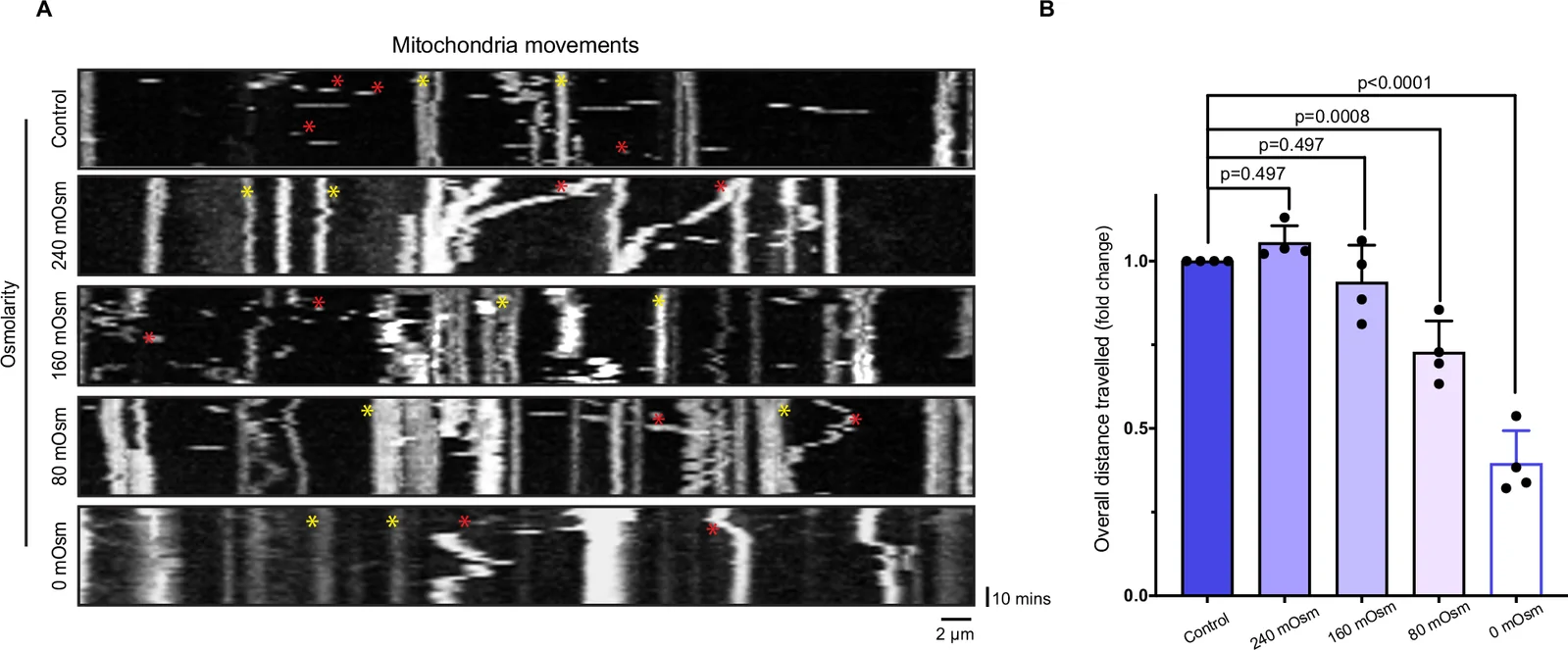
Mild hypotonic conditions preserve mitochondrial motility. **A** Representative kymographs are shown for different conditions. Examples of immotile mitochondria are indicated by yellow asterisks and the motile ones by red asterisks. Representative data are shown from three independent biological replicates. **B** The overall distance traveled by mitochondria over 5 min was quantified and normalized to the corresponding control condition (before osmolarity change). Data are shown as bar graphs (mean ± SD) overlaid with individual data points. Each data point represents the overall motility of mitochondria normalized to respective controls in an individual experiment. *N* = 3 independent biological experiments. *p*-values indicate comparisons to the Control group: 240 mOsm (*p* = 0.497), 160 mOsm (*p* = 0.497), 80 mOsm (*p* = 0.0008), and 0 mOsm (*p* < 0.0001). Significance was evaluated using one-way ANOVA with Holm-Šídák’s multiple comparisons test, degree of freedom is 15. The corresponding video for this experiment is shown in Supplementary Movie 2. Source data are provided as a Source Data file.

Incubation of neuronal cultures in hypotonic media was used to increase membrane tension, a step necessary to perform membrane rupture and high-contrast imaging of intracellular components by cryo-EM. We used thalamus neural explants, which produced long neurites extending from brain tissue, allowing unambiguous assignment to axonal origins at this developmental stage^25,33,34^. To minimize the effect of hypotonic stress on gross axon morphology, we optimized the conditions using light microscopy (Fig. 2). The osmolarity of normal neural explant media was approximately 320 mOsm at 8 days in vitro (DIV8). We exposed thalamic explants to media with osmolarities of 240 mOsm, 160 mOsm, and 80 mOsm, respectively (Fig. 2A, B). Consistent with previous studies showing the stability of neuronal morphology under osmotic stress^35,36^, we found that the overall morphology of axons remained unaffected at the light microscopy level during the first 10 min of exposure to hypotonic media down to 160 mOsm (Fig. 2A–D). However, an incubation in 80 mOsm hypotonic solution caused small swellings on axon membranes (Fig. 2C, D), indicative of membrane instability (Supplementary Movie 1). In all conditions, no axonal breakage was observed after 10 min of exposure, suggesting that short-term hypotonic exposure does not cause gross axonal morphological disruption under the conditions tested^35–37^. We further examined the impact of hypotonic stress on intracellular dynamics by measuring mitochondrial motility within axons using light microscopy. The motility of mitochondria within axons, a sensitive indicator of cellular health^38^, was tracked under various hypotonic conditions using MitoTracker (Fig. 3, Supplementary Movie 2). No significant change in mitochondrial motility was observed at 240 mOsm and 160 mOsm compared to the control at 320 mOsm, while a marked reduction in motility was noted at 80 mOsm and 0 mOsm (Fig. 3A-B and Fig. S1A-E), indicating impaired axonal transport under severe hypotonic stress. Neurons remained morphologically intact when exposed to 160 mOsm for an extended duration of up to 24 h (Fig. S1F and S1G), showing hypotonic tolerance of neurons at 160 mOsm.

### Controlled osmotic and mechanical disruption defines the *Ghost Neuron* state

**Fig. 4:**
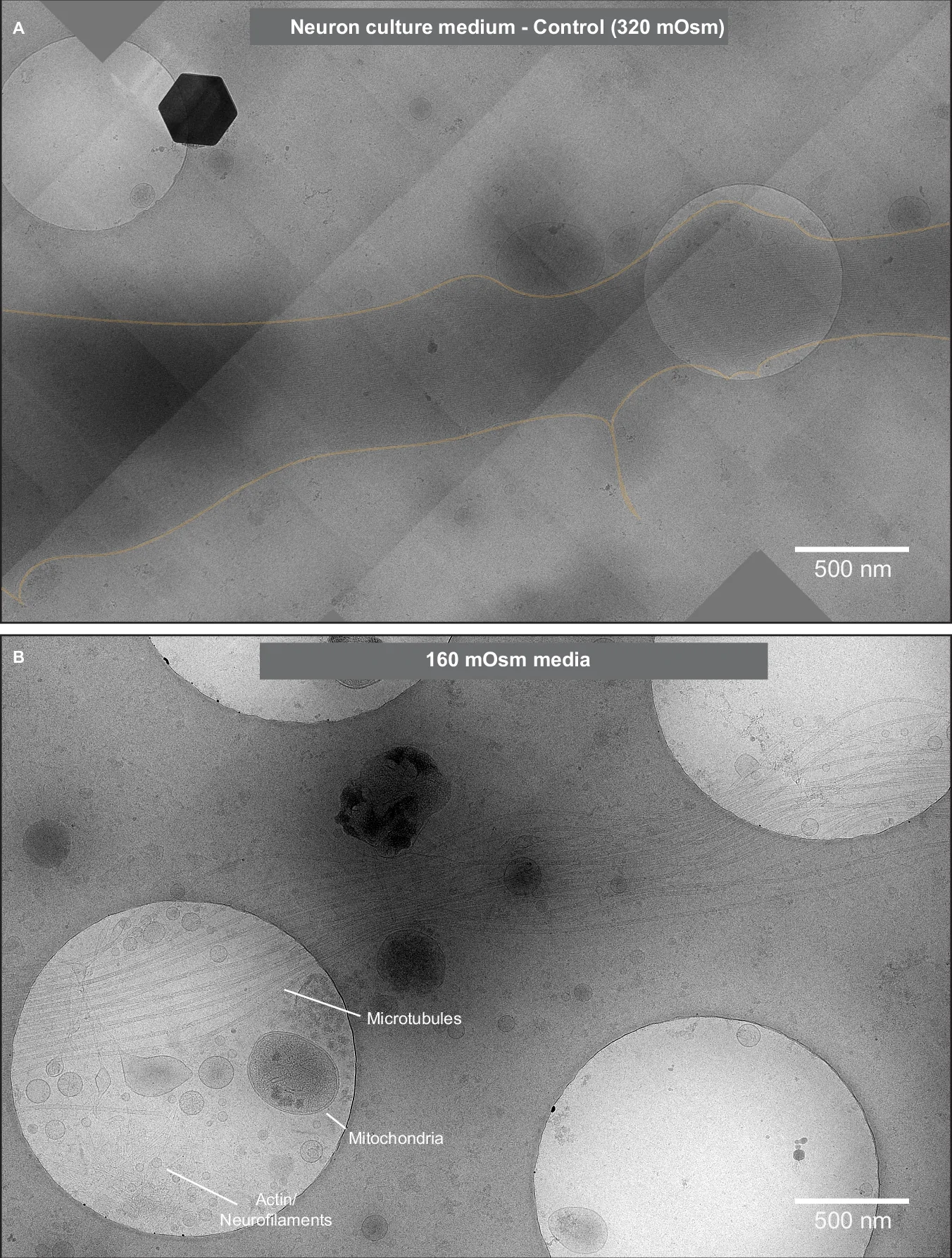
*Ghost Neuron* visualized by cryo-EM. **A** High-magnification montage (HMM) of a DIV8 thalamus explant axon in normal culture media, showing an intact plasma membrane after cryo-EM sample preparation. **B** High-magnification montage of a *Ghost Neuron* prepared by exposure to hypotonic media at 160 mOsm and subsequent blotting and vitrification. Note the high-contrast visualization of microtubules, mitochondria, actin, and neurofilaments. Representative images are shown from three independent biological replicates.

To generate *Ghost Neurons*, we incubated thalamus explants cultured on EM grids until DIV7-9 and exposed them to hypotonic media (240, 160, 80, and 0 mOsm) for 5 min, followed by blotting and vitrification. High-magnification montages (HMMs) revealed that membranes remained mostly intact at 320 mOsm (control, Fig. 4A) and 240 mOsm (Fig. S2A), whereas the treatment at 160 mOsm and 80 mOsm partially disrupted the plasma membrane (Fig. 4B and Fig. S2B). Notably, exposure to 0 mOsm caused pronounced fragmentation under cryo-EM, indicating severe osmotic stress (Fig. S2C). While the 80 mOsm condition avoided gross fragmentation, mitochondrial transport analysis showed impairment under this condition (Fig. 3). We therefore selected 160 mOsm as the optimal compromise, permitting controlled membrane removal while preserving neurite geometry and intracellular dynamics as assessed by mitochondrial motility.

**Fig. 5:**
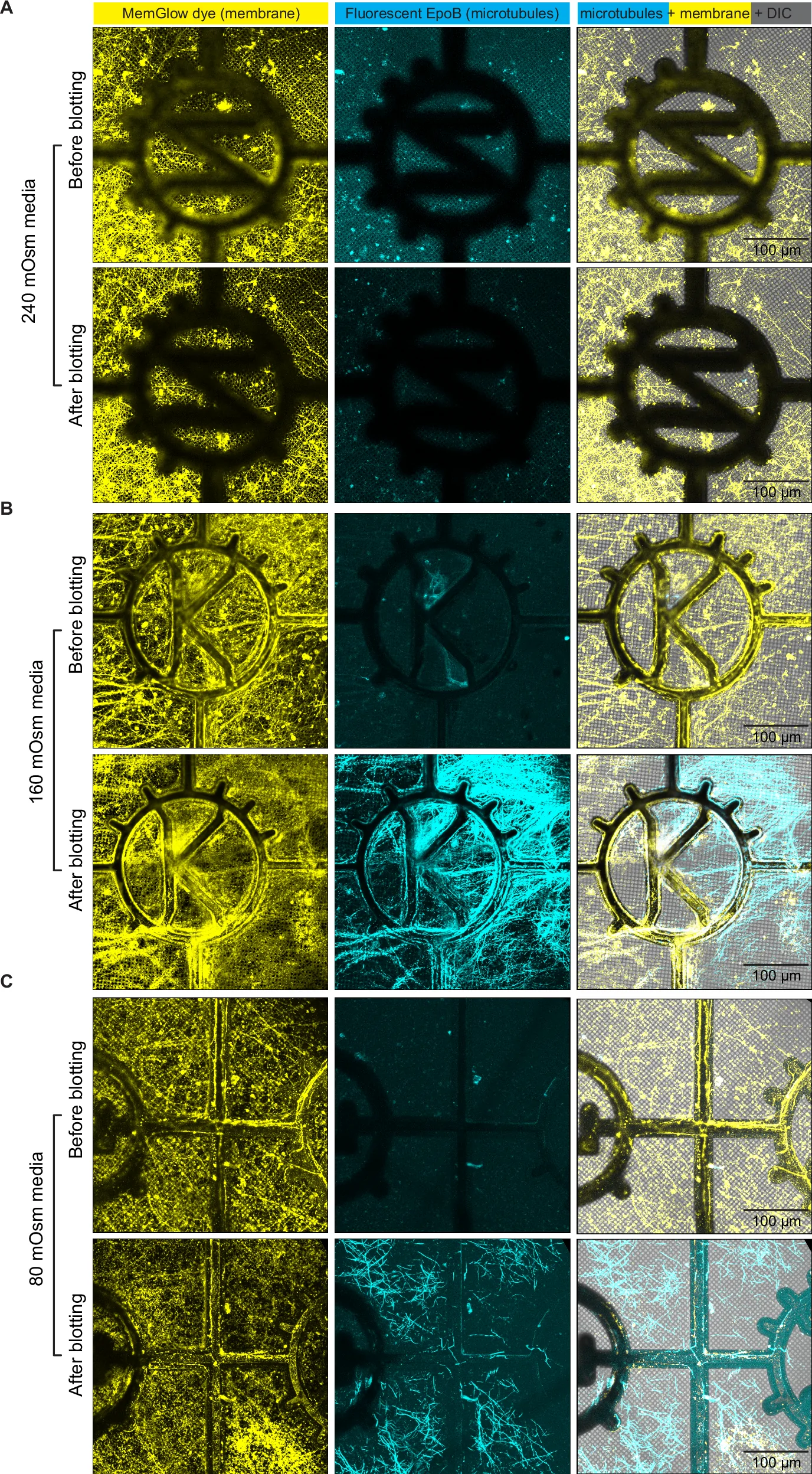
Combined hypotonic treatment and mechanical blotting leads to plasma membrane rupture, facilitating *Ghost Neuron* preparation. Thalamus explants stained with MemGlow (yellow) and fluorescently-conjugated EpoB (cyan) were exposed to varying hypotonic conditions at 240 mOsm (**A**), 160 mOsm (**B**), or 80 mOsm (**C**) and manually blotted with filter paper. The same regions before and after blotting are shown. Representative images are shown from three independent biological replicates. The corresponding video for this experiment is shown in Supplementary Movie 4.

To determine when membrane removal occurs during preparation, we assessed membrane integrity using fluorescently labeled Epothilone B (EpoB)^39^, a microtubule-bound compound, which is not cell-membrane permeable up to 2 µM (500 nM fluorescent-EpoB tested; Fig. S3, ‘Before blotting’). As a positive control, 0.05% (v/v) Triton X-100 permeabilization allowed intracellular labeling (Fig. S3 and Supplementary Movie 3). Neurons exposed only to hypotonic media showed no labeling, indicating hypotonic treatment for 10 min alone does not disrupt membranes (Fig. 5 ‘Before blotting’, Supplementary Movie 4). When hypotonic treatment was followed by filter paper blotting, but without vitrification, robust microtubule labeling was observed under 160 and 80 mOsm conditions, while samples treated with 240 mOsm media showed no labeling (Fig. 5, Supplementary Movie 4). These observations were consistent with our cryo-EM observation (Fig. 4 and Fig. S2) and demonstrated that membrane disruption occurs during the mechanical blotting following exposure to hypotonic media, establishing a controlled *Ghost Neuron* preparation.

### *The Ghost Neuron* preparation preserves axonal trajectories and major cytoskeletal architecture

The *Ghost Neuron* preparation largely preserves the linear trajectory and bundled organization of axonal microtubules following membrane removal. High-magnification montages (HMMs) show that microtubule bundles remain aligned along continuous neurite paths (Fig. 4B, Fig. S2B and S4), consistent with trajectories observed prior to vitrification (Fig. S4B). Mitochondria within these regions retain their short, oval morphology (Fig. 4B and Fig. S4), characteristic to axonal mitochondria, rather than the elongated forms commonly observed in dendrites^25,40^.

As expected for a membrane-removal procedure, some cytoplasmic material became redistributed during blotting, scattering small vesicles, membrane fragments, and soluble macromolecular components into adjacent grid holes (Fig. S4A). In contrast, larger organelles and the microtubule cytoskeleton followed the original trajectory of the neurite, allowing the visualization of its spatial organization and potential interactions with associated components (Fig. S5A and S5B). These montages therefore provide a topological cellular map that allows spatially resolved cryo-EM acquisition and structural back-mapping along defined neurite segments.

### *The Ghost Neuron* application: High-resolution structural analysis of F-actin and Cofilactin

**Fig. 6:**
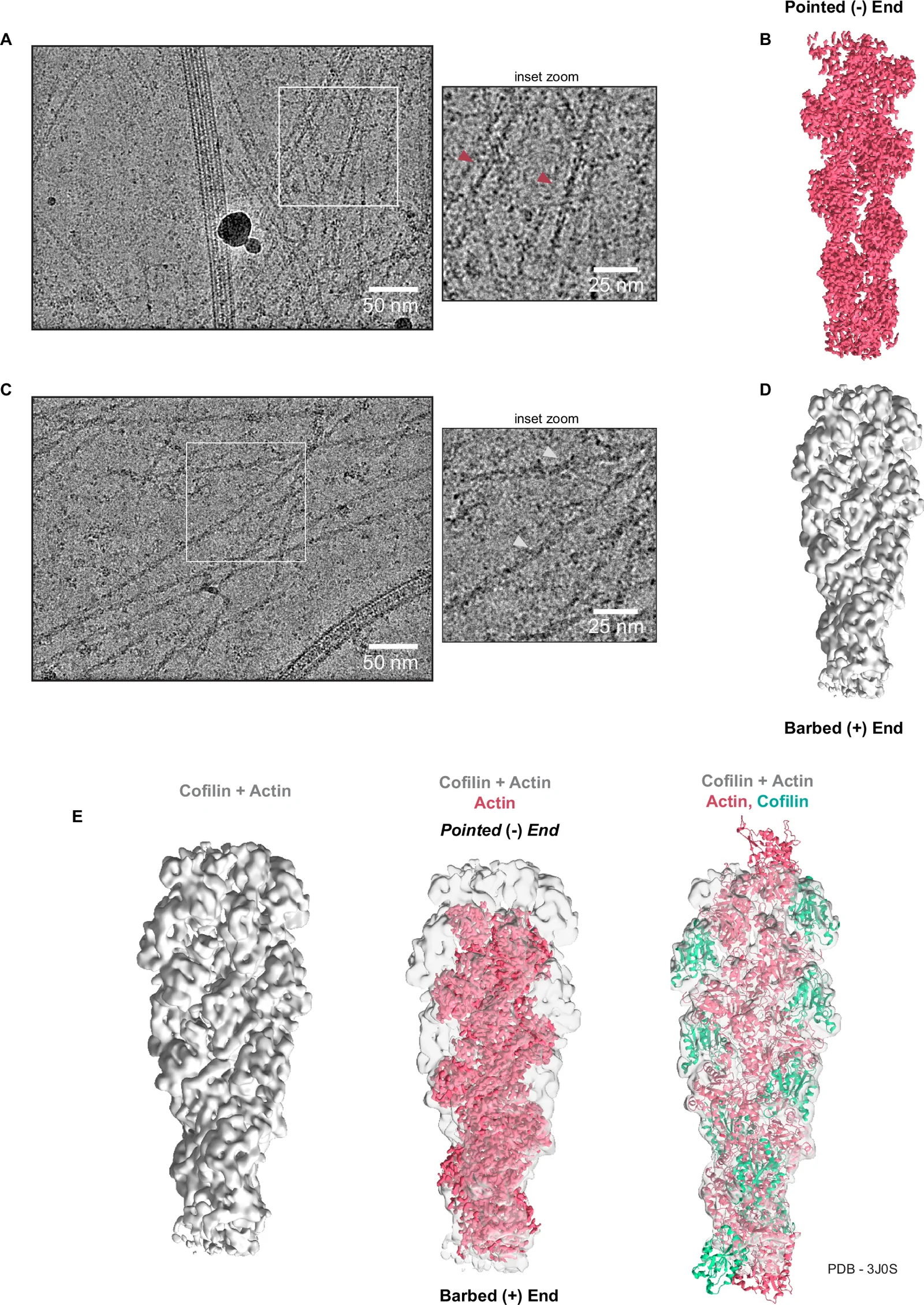
Single-particle analysis of actin filaments from *Ghost Neurons*. **A** Representative cryo-EM image of F-actin filaments. The inset shows a magnified view for better visualization with F-actin indicated by red arrowheads. **B** 3D reconstruction of F-actin up to 3.24 Å resolution. **C** Representative cryo-EM image of cofilactin filaments. The inset shows a magnified view with cofilactin filaments highlighted by white arrowheads. All actin filaments in this view are decorated with cofilin. Images in (**A**–**C**) are representative of three independent biological replicates. **D** 3D reconstruction of cofilin-decorated actin filaments at 5.32 Å resolution. **E** The reconstructed map of cofilactin is compared to the published in vitro structure of cofilactin (PDB 6vao), shown in cartoon representation with F-actin in red and cofilin in green.

The improved contrast due to the *Ghost Neuron* preparation facilitates high-resolution SPA of macromolecular assemblies. Using SPA on filament segments collected from *Ghost Neuron* preparations, we reconstructed neuronal F-actin at 3.24 Å resolution (Fig. 6A, B). Notably, we also identified a distinct subpopulation of F-actin that was fully decorated on its surface. These filaments were initially recognizable in the images by their characteristic increased diameter (Fig. 6C and Fig. S6), and this visual distinction was subsequently validated by 2D classification of the filament segments, which separated decorated from undecorated particles. Specifically, in our dataset, of the 1528 µm total length of analyzed F-actin from various areas, 21.7 µm showed continuous decoration. While we collected only 2328 asymmetric particles from this subset, we achieved a structure at 5.32 Å resolution (Fig. 6C, D). Secondary structure assignment and backbone tracing revealed cofilin as the decorating protein, consistent with the available structure of an in vitro prepared cofilin-actin complex (PDB 6vao) (Fig. 6E) with a fit correlation coefficient of 0.97. We also observed that the actin filaments decorated by cofilin adopt an expanded conformation (C-form), which has also been observed upon full binding of cofilin. Interestingly, in contrast to in vitro studies reporting discontinuous cofilin clustered association along actin filaments^41^, our observed F-actin tended to appear predominantly either fully decorated or largely undecorated, rather than showing partial or discontinuous cofilin binding. This extensive decoration of cofilin on actin filaments is consistent with in situ cryo-ET studies that identified fully decorated cofilin-actin filaments at the growth cone of axons^42^, within the lumen of microtubules in Drosophila S2 cells and platelets^43,44^. We have not observed actin filaments within the lumen of neuronal microtubules, suggesting this phenomenon may be a cell type-specific mechanism. Altogether, these observations highlight the strength of the *Ghost Neuron* approach in uncovering molecular architectures within neurons.

### *The Ghost Neuron* application: High-resolution conformational analysis of microtubules in a cellular context

**Fig. 7:**
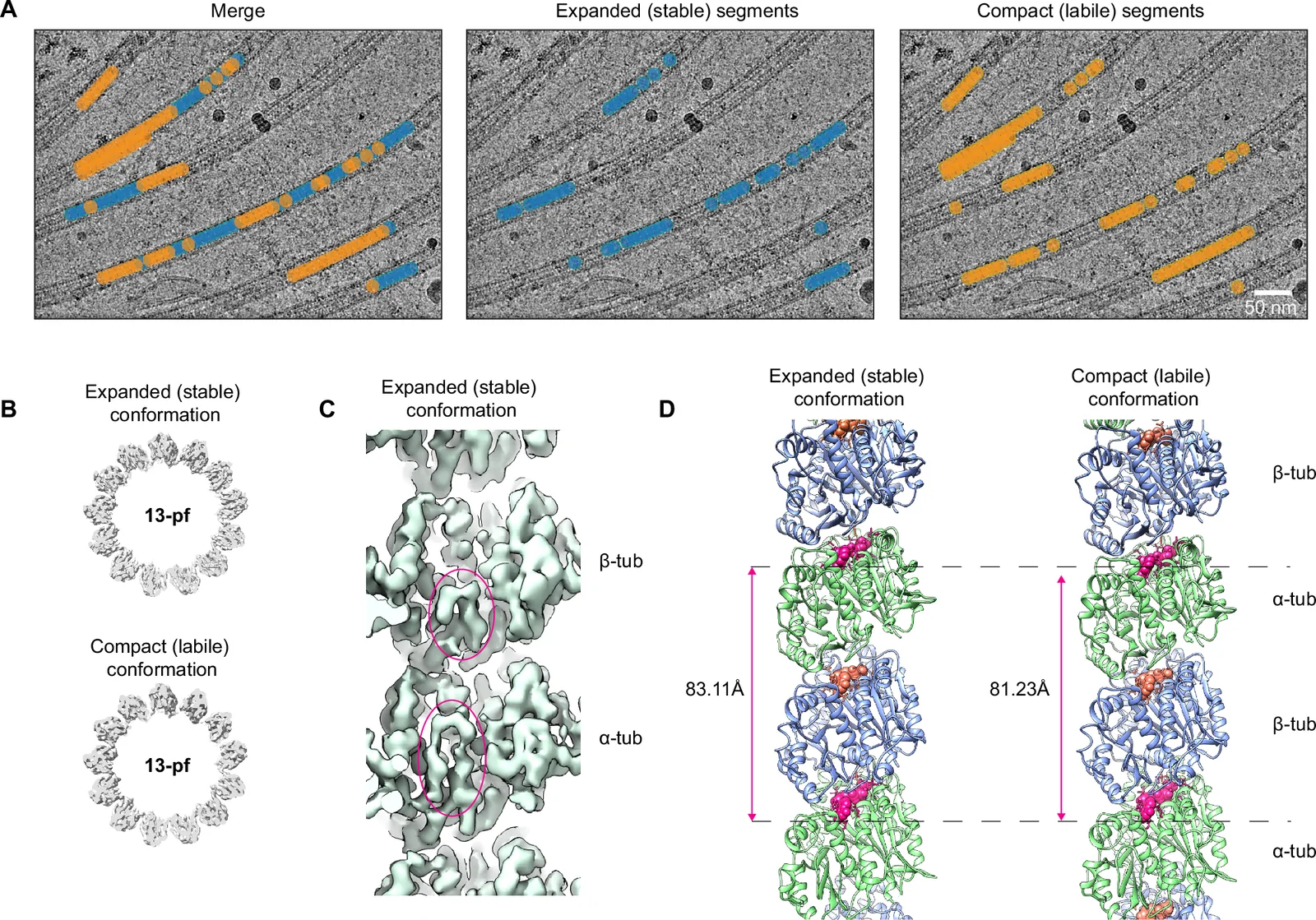
Single-particle analysis of microtubules from *Ghost Neurons*. **A** Representative micrographs of microtubules analyzed for their conformational states. Microtubule segments that are in the expanded conformation are shown in blue and those in the compact conformation in orange. Uncolored microtubule regions were not used in this analysis. Representative images are shown from three independent biological replicates. **B** Both conformations display a 13-protofilament arrangement. **C** Identification of α- and β-tubulin subunits within the heterodimer. The S9-S10 loop on the inner-lumen (magenta circles) exhibits distinct densities, serving as a marker for subunit identification and quality control. **D** Atomic model of the expanded and compact microtubule segments showing inter-dimer distances of 83.11 Å and 81.23 Å, respectively. Pink: nucleotide bound to α-tubulin, Salmon: nucleotide bound to β-tubulin.

Furthermore, the *Ghost Neuron* technique facilitates SPA analysis of microtubules along their neurite trajectories (Fig. S7). Classification revealed two major lattice conformations at resolutions of 3.59 Å and 3.9 Å, respectively, both displaying the canonical 13-protofilament architecture (Fig. 7). Approximately 61% of the classified microtubule segments adopted the expanded lattice conformation with an inter-dimer spacing of 83.1 Å, while 39% displayed a compact lattice with an 81.2 Å repeat (Fig. 7D). Independent classification runs yielded highly consistent class assignments ( > 90% consistency) (Fig. S8), indicating robust separation of the two states. The measured axial spacings match the well-established expanded (83 Å) and compact (81 Å) lattice spacing described for in vitro reconstituted microtubules^45–47^. Similar expanded lattice spacing was observed in our previous reconstruction of Epothilone B-stabilized microtubules at 3.19 Å resolution in regenerating axons^34^, where membrane rupture also occurred during vitrification due to expanded membranes at regeneration sites, indicating that membrane disruption itself does not distort lattice geometry. In addition, the expanded lattice states have been detected in intact mammalian U2OS cells by cryo-ET^48^, supporting the lattice heterogeneity within a cell. Notably, our SPA-based *Ghost Neuron* analysis resolves lattice conformations locally along individual microtubules. By segmenting filaments at ~82 Å intervals and classifying using C1 refinement with a focused mask spanning two protofilaments, each particle reports the local conformation of a lattice patch at approximately single-heterodimer resolution. Therefore, this approach directly reveals co-existence of expanded patches (majority), and compact patches (minority) along single neuronal microtubules at a single-molecular resolution, rather than averaging lattice states over extended regions.

### *The Ghost Neuron* application: Montages bridge cellular context and high-resolution structural information

**Fig. 8:**
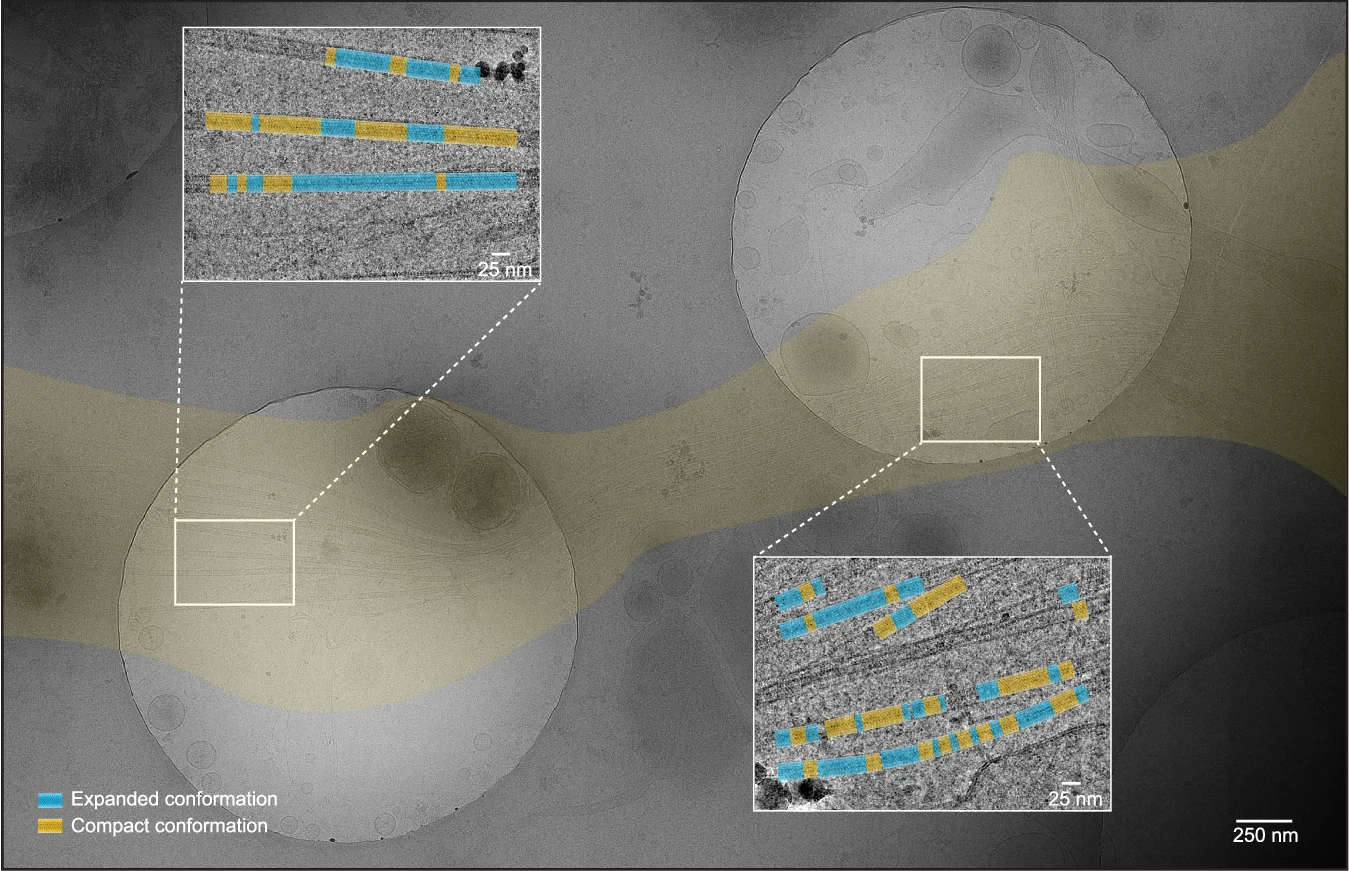
Spatial mapping of microtubule conformational states in *Ghost Neurons*. Expanded and compact microtubule segments are back-plotted onto high-magnification cryo-EM montages of *Ghost Neurons*, linking local structural states to neurite context. Note that segments without a colored assignment are not used for the analysis and do not represent another lattice state.

Back-plotting of high-resolution structural states onto neurite trajectories provides spatial context that is difficult to obtain by cryo-ET of intact cells or extraction-based methods. We validated axonal identity of the neurites by measuring the angular distribution of microtubules relative to neurite direction which follows a plus-end-out alignment with angles of less than 90˚ (Fig. S7B). Using back-plotting, we mapped the high-resolution microtubule states onto HMMs, thereby linking local molecular conformation to the neuronal context. This mapping revealed that expanded lattice segments were not confined to microtubule ends where GTP-tubulin is typically enriched (Fig. S7, asterisk)^49^, but also occurred as discrete patches within the mid-shaft of individual microtubules (Fig. 8 and Fig. S7). These observations indicate the presence of heterogeneous lattice conformations along individual microtubules, which might contribute to the regional specialization of axonal microtubules (Fig. 8 and Fig. S7C), although the molecular origin and functional consequence remain to be determined.

Microtubule lattice spacing has classically been linked to nucleotide state, with expanded lattices associated with GTP-like states and compact lattices associated with GDP-tubulin^45,50,51^. This classical view describes newly polymerized plus ends, where GTP-tubulin is incorporated and subsequently hydrolyzed as the microtubules mature^45^^,^^50,51^. However, within mature lattice interiors, this relationship between lattice spacing, nucleotide state, and microtubule stability is likely more complex, particularly in axons. Recent work reported that axonal microtubules in human iPSC-derived neurons are GDP-bound yet adopt an expanded, GTP-like lattice, indicating that lattice spacing is not determined solely by nucleotide state^52^. Our observation of the dominant expanded microtubule conformation is consistent with this view, while the detection of compact segments suggests that local lattice heterogeneity is detectable in our dataset. Differences in neuronal origin, developmental stage, sampled axonal region, or analysis strategy may influence the extent by which compact lattice segments are detected.

Two models have been proposed to explain microtubule stability in axons: one suggests that centrally located microtubules are generally more stable while peripheral ones are more dynamic^53–55^, and the other model suggests intra-microtubule heterogeneity, in which individual microtubules contain alternating domains with differing stability^56,57^. While these models are not mutually exclusive, our structural analysis is consistent with intra-microtubule heterogeneity, revealing local differences in lattice conformation within single axonal microtubules. Such regional variation is consistent with previous studies^58^and suggest that lattice conformation can be locally influenced by lattice-binding microtuble-associated proteins (MAPs)^59–61^, post-translational modifications, mechanical stress^50,51^, or repair processes^62–66^. Thus, we interpret the expanded and compact segments identified here as local structural states of axonal microtubules, though molecular determinants and functional consequences remain to be established.

## Discussion

Neurons exhibit extreme polarization, with specialized subcellular microdomains distributed along elongated neurites^67^, including growth cones^68^, sites of branch initiation^25,69^, synaptic terminals^70^, localized translation hotspots^71^, and transport- and signaling hubs^72^. Structural exploration of these confined regions requires the preservation of spatial organization together with sufficient image contrast for an analysis at molecular resolution. However, intact neurites face inherent difficulties for high resolution imaging by cryo-EM and cryo-ET, as the dense cytoplasmic environment inside neurites limits contrast and restricts achievable resolution. We established the *Ghost Neuron* approach to address this constraint by selectively removing the plasma membrane immediately prior to vitrification, resulting in reduced cytoplasmic density and locally thinning of the specimen, while maintaining the architectural continuity of neurite trajectories. The method facilitates structural analysis at defined positions along neurites and allows individual molecular states to be mapped back onto specific neuronal locations. High-magnification montages along extended neurite trajectories provide a spatial reference map for integrating distinct molecular states resolved by high-resolution SPA analysis. In this way, the *Ghost Neuron* method bridges neuronal architecture and structural analysis at molecular resolution within a single specimen.

The *Ghost Neuron* is unique in its direct coupling of tension-mediated membrane destabilization with immediate vitrification, thereby preserving macromolecular assemblies in situ in a frozen-hydrated state compatible with high-resolution cryo-EM. In contrast, classical unroofing or detergent-based extraction methods typically require chemical fixation or metal coating prior to imaging to preserve subcellular architecture and therefore, are incompatible with molecular-resolution cryo-EM of hydrated specimens. Among available strategies to modulate membrane tension in neurons, we found mild hypotonic perturbation to be minimally invasive. Our evaluation of intracellular transport demonstrates that, optimized hypotonic modulation preserves overall intracellular organization and dynamics including cytoskeletal elements and organelles, and motor-driven dynamics. Alternative perturbations such as mechanical stretching^73^, RhoA-ROCK activation^74–76^, or the modification of lipid composition^77^ can induce significant disruption ranging from membrane-cytoskeleton decoupling, contractility-induced retraction to altered lipid composition and gene expression. Short-term hypotonic treatment provides a practical strategy to transiently weaken membranes while maintaining intracellular organization for cryo-EM applications and keeping cells in a vital state.

In this study, we demonstrate the capabilities of the *Ghost Neuron* approach through high-resolution structural analysis of F-actin and microtubules. We resolved F-actin at molecular resolution and identified a sub-population of fully cofilin-decorated filaments. We also mapped compact and expanded microtubule lattice conformations along individual axons, which provides new insights into the conformational variability of microtubules. These examples illustrate how enhanced contrast combined with preserved neurite topology facilitates the interpretation of molecular states within their cellular geometry and context. A potential concern is whether osmotic modulation and membrane rupture during the *Ghost Neuron* preparation could themselves induce conformational changes, such as microtubule lattice transitions. Several observations make this scenario unlikely. The measured axial repeat distances correspond closely to canonical compact and expanded lattice parameters reported in vitro^45–47^ and in intact cells^48^. Lattice classifications were reproducible across independent runs (Fig. S8) and multiple neurites (Fig. S7). In addition, membrane rupture occurs immediately prior to vitrification, limiting the opportunity for coordinated lattice remodeling, such as nucleotide exchange, tubulin incorporation, or patterned MAP recruitment that would generate expanded lattice patches. Together, these considerations are consistent with the interpretation that the lattice heterogeneity observed in *Ghost Neuron* largely reflects pre-existing structural states rather than rupture-induced remodeling. The molecular origin and functional significance of conformational heterogeneity within neuronal microtubules remain open questions and will require further investigation.

Besides preserving neurite trajectories for spatial mapping, the *Ghost Neuron* preparation generates a dispersed intracellular material across EM grids. Despite the fact that these particles lose precise positional information, they can still be useful for structural analysis. Previous studies have shown that crude or partially extracted intracellular mixtures can yield high-resolution SPA reconstructions^78–80^, highlighting that such fractions can serve as important resources for structural discovery. Thus, the *Ghost Neuron* approach provides two complementary structural readouts within a single specimen: a continuous cytoskeletal scaffold for topological mapping and a dispersed pool of endogenous assemblies amenable to high-resolution SPA. While this study focuses on cytoskeleton as proof-of-principle targets, this feature may be useful of finding neuronal complexes of various functions.

The *Ghost Neuron* approach can be applied not only to primary neuronal tissues, but also to various neuronal cell types including iPSC-derived neurons, as well as those derived from genetically modified models such as knockout mice, disease models, and neurons expressing fluorescent markers. Cultured neurons prepared on EM grids can be imaged directly by light microscopy either during live imaging or after vitrification, and the resulting fluorescence data can be correlated to map the regions of interest for further imaging by cryo-EM. Future efforts in adapting the latest advances in deep learning^81–83^ will improve the identification of subcellular structures and enhance averaging techniques for cytoskeleton-associated proteins and other macromolecular complexes within neurons. By improving contrast and allowing access to otherwise difficult-to-visualize compartments, the *Ghost Neuron* platform expands the capabilities of cellular structural biology. These methodological advantages make the *Ghost Neuron* a highly versatile and powerful tool for integrative structural studies of neuronal architecture and function.

### Limitations

The *Ghost Neuron* preparation is designed to preserve the geometric organization of the axonal cytoskeleton while facilitating high-resolution cryo-EM analysis; however, it has inherent limitations. Since the plasma membrane is deliberately ruptured immediately prior to vitrification, processes associated with membrane cannot be analyzed in their native membrane environment. Examples include receptor signaling and ion channel organization. In addition, selective membrane removal disrupts submembranous structures that are mechanically coupled to the plasma membrane. A prominent example is the membrane-associated periodic skeleton (MPS)^84,85^, composed of actin rings interconnected by spectrin tetramers. After *Ghost Neuron* preparation, actin filaments remain axially localized along neurites, but the periodic pattern of the MPS is not preserved. This also reflects the difficulty in cryo-EM of neurons of resolving membrane-associated cytoskeleton structures. Finally, membrane rupture leads to partial redistribution of soluble and small membranous components, and it is not possible to fully determine which species are retained versus released in any given preparation. As a result, the *Ghost Neuron* imaging does not provide a complete inventory of all intracellular constituents at their original locations. Instead, the method is best suited for high-resolution structural analysis of selected or retained assemblies.

## Methods

### Grid Preparation for neural primary cell culture

Gold grids from Quantifoil (R2/4 200 mesh, R2/1 200 mesh or Multi A 300 mesh or R3.5/1 H15 finder, 135 mesh) were used for culturing neurons. Before coating, grids were glow discharged in residual air using a Pelco easiGlow for 60 s at 15 mA and 0.38 mbar. Immediately after glow discharge, the grids were transferred to a cell culture dish (Greiner bio-one #627170), immersed in 2 ml of 1 mg/ml Poly-L-lysine (Sigma-Aldrich #P2636) in 0.1 M borate buffer (di-Sodium tetraborate decahydrate, Carl Roth #1303-96-4) followed by incubattion of overnight at 37 °C.

The next day, grids were washed three times with either 1x PBS (Gibco #10010049) or H_2_O to remove excess Poly-L-lysine, and coated with laminin (Gibco #23017015) at 5 μg/ml for at least two hours at 37 °C. After laminin coating, the grids were kept submerged in Neurobasal medium (Gibco #21103049) at 37 °C until neuronal explants or cells were planted.

### Preparation of primary *Mus musculus* thalamus explants and dissociated neuron cultures

All animal procedures were approved by the Animal Care and Use Committee (ACUC) of the National Heart, Lung, and Blood Institute (NHLBI) animal protocol H-0331 in accordance with NIH research guidelines for the care and use of laboratory animals. Cultures were prepared from E15.5 embryos of CD-1 mice (*Mus musculus*, outbred CD-1/ICR genetic background, Charles River Laboratories). Thalamus tissue was dissected from embryonic brain and placed in cold Hanks’ Balanced Salt Solution (HBSS, Gibco #14170112) supplemented with 1x HEPES (Gibco #15630080). The thalamus tissue was then cut into smaller fragments and placed into fresh media. For explant cultures, the thalamus tissue with a size of approximately 200 μm was placed onto coated μ-Slide 8-well dishes (Ibidi, #80826) or coated EM grids (see grid preparation above) in plating medium (Neurobasal medium supplemented with 1x B27 (Gibco, #17504044) and 1x Glutamax (Gibco #35050061)). For dissociated cells, hippocampi were treated with 1x Trypsin-EDTA (0.5%, Gibco #15400054) and 1% (w/v) DNase I (Roche #10104159001) for 12-15 min at 37 °C. Afterwards, excess trypsin was removed by washing the hippocampi three times with plating medium. Cells were dissociated by trituration using a 1 ml pipet tip in 2 ml plating medium and then, passed through a 70 µm nylon cell strainer (Corning #431751) before plating on glass dishes or EM grids (see above). Every 3 days in vitro, half the culture medium was exchanged to fresh media.

### Live cell imaging and analysis

*Mus musculus* primary explant tissue was cultured on μ-Slide 8-well or EM-coated grids for 8–9 days in vitro at 37 °C and 5% CO_2_. To visualize the plasma membrane and mitochondria, we used 2 nM MemGlow 488 (Cytoskeleton #MG01) or 2 nM MitoTracker Red CMXRos (Thermo Fisher Scientific #M7512) for 1 min, respectively. To visualize microtubules in membrane-compromised axons, we used 500 nM fluorescently labeled Epothilone-B^39^.

For experiments analyzing mitochondrial transport in hypotonic solutions, neurons were first imaged in standard media labeled with MemGlow and MitoTracker dye for 5 min. While the dish remained under the microscope, the media was switched to hypotonic media with a specific osmolarity (240 mOsm, 160 mOsm, 80 mOsm, or 0 mOsm, respectively) to ensure imaging of the exact same location before and after hypotonic treatment.

To mimic cryo-EM specimen preparation, the grid medium was replaced with hypotonic media containing MemGlow 560 (1:5000 dilution) and 500 nM fluorescently labeled Epothilone-B for at least 10 min, and the grids were imaged by Differential Interference Contrast (DIC) and fluorescence methods. Subsequently, the grids were manually back-blotted for 3 or 4 sec using standard vitrification filter paper (Grade 1, Cytiva #1001-055 or Grade 595, Ted Pella #47000-100). After returning the grids to the imaging chamber, the pre-blotting regions of interest were relocated using the finder grid coordinates and re-imaged to assess the effects of the *Ghost Neuron* preparation. Due to the imperfect attachment of grids to the glass surface, achieving a uniform focus in the imaging area was challenging. To address this, we imaged the entire z-stack covering different planes of the signal. The movies were drift-corrected using the StackReg plugin from ImageJ. Corresponding movies are shown in Supplementary Movie 4. To correlate neurite trajectories before and after the *Ghost Neuron* preparation, thalamus explants cultured on EM grids were live-imaged using fluorescence microscopy in the presence of 100 nM SPY-tubulin (Cytoskeleton #CY-SC503). Following the *Ghost Neuron* preparation and subsequent vitrification, these initial fluorescence images were spatially aligned with high-magnification cryo-EM montages (HHMs) of the corresponding grid regions. To assess long-term hypotonic effects on neurite growth, media was exchanged with various osmolarities. Large-area montages were acquired at 0 h and, using photo-etched dish coordinates, at the identical locations after 24 h. Montages were randomly divided into blocks for analysis. DIC images were preprocessed using an FFT filter for flat-field correction, followed by neurite segmentation using edge detection in ImageJ. Thresholds were adjusted to calculate the neurite area fraction for the same regions at 0 and 24 h. Results are displayed as the fold change in area fraction ± SEM. All movies were recorded using a Nikon A1R microscope with a 60x oil-immersion objective and a Tokai Hit stage-top incubator set to 37 °C and 5% CO_2_. The microscope was controlled using NIS-ELEMENTS software (Nikon).

### Osmolarity measurements

Osmolarity data was collected using an Osmette III automated osmometer (Precision Systems Model 5010) measuring freezing point depression. Before testing samples, the machine was cooled down, and calibrated using ultrapure H_2_O, a 100 mOsm standard, and a 500 mOsm standard. A cleanette was used to thoroughly clean the sample well and remove any moisture. An Osmette pipette tip was used to draw up 10 µl of a conditioned media aliquot. The tip was placed in the sample well after wiping excess sample from the outside surface. The sample was cooled, and the freezing point depression was read to determine an osmolarity value in mOsm. The sample well was cleaned before reading the next sample.

### Analysis of mitochondria movements

Mitochondria movements were recorded using a Nikon A1R microscope at an interval of 20 s in normal culture media and hypotonic media, and analyzed in ImageJ equipped with the TrackMate plugin. The same field of view was recorded before and after changing the media osmolarity. Briefly, mitochondria signals were detected using the LoG detector with size parameters as 1.5–2 microns. Different quality thresholds were applied to filter out false positives as needed. Once the organelles were detected, the LAP tracker algorithm was applied to connect the tracks detected in the previous step. The data obtained was processed in Microsoft Excel. Overall distance traveled in the time period of 5 min was compared across conditions. Total distance traveled less than 2 µm (approximate size of mitochondria in the axons) was considered as immotile. The data was plotted as scatter dot plots for individual experiments using PRISM software and are shown in Fig. S1A or shown as bar graphs in Fig. 2, in which the average distance traveled was normalized to its corresponding controls (before exchanging the media to hypotonic conditions).

### Cryo-EM sample preparation

*Mus musculus* primary thalamus explant tissue or dissociated hippocampal neurons were cultured on coated cryo-EM grids as previously described^25,34,86^ for 7–9 days (see also grid preparation above). At DIV7-9, media was diluted with ultrapure water to reach the designated osmolarity 5 min prior to blotting. The grids were blotted using type 595 filter paper (Ted Pella #47000-100) with blot force 4 s, blot time 4 s, delay time 0 s and the humidity chamber set to 30 °C and 100% humidity and vitrified in liquid ethane using a Vitrobot Mark VI (Thermo Fisher Scientific) or for blotting from the backside of the grids, a Leica EM GP2 plunge freezer with a blotting time of 4 s. The grids were then screened using a Glacios Transmission Electron Microscope (TEM) operating at 200 kV and equipped with a Falcon 4 direct electron detector (Thermo Fisher Scientific) to determine ice thickness.

### High-magnification montages (HMMs) of thalamus explants

Polygon-montage images covering the region of interest drawn around axons were acquired on a Titan Krios G4 (Thermo Fisher Scientific) equipped with a Gatan BioContinuum energy filter and a K3 Summit direct detector at an acceleration voltage of 300 kV. The images were acquired at 26,000x corresponding to a pixel size of 6.62 Å at a defocus of −15 µm. The acquired maps were stitched and converted to images using a script written in SerialEM software. Montages were acquired for regions where spatial context or cellular mapping was required (e.g., for back-plotting microtubule lattice states). For SPA datasets collected solely for high-resolution structural determination, montage acquisition was not necessary. All structures for which spatial mapping is shown in this study were derived from regions with corresponding montages.

### Single-particle analysis (SPA) of microtubules in neurites

The SPA workflow of microtubules is shown in Fig. S8A. Movies (40 frames) of *Ghost Neurons* were acquired on a Titan Krios G4 (Thermo Fisher Scientific) operating at 300 kV and equipped with a Gatan Biocontinuum energy filter and a K3 Summit direct electron detector. Data was collected using the SerialEM software at a magnification of 105,000x, corresponding to a calibrated pixel size of 0.412 Å in super-resolution mode. The defocus range was set between −0.8 and −3 μm and the total accumulated electron dose was 52.2 e-/Å^2^. Movies were motion-corrected and downsampled in CryoSPARC resulting in a pixel size of 0.824 Å. 4172 micrographs containing 7787 microtubules were used for analysis, corresponding to a cumulative microtubule length of 1296 μm. Initial particle picking, 3D classification, and reconstructions were performed using CryoSPARC. Microtubules were traced and segmented (158,108 segments) using overlapping boxes with a step size of 82 Å, matching the length of one tubulin heterodimer. Microtubule particle extractions were carried out with a box size of 840 pixel. Following particle cleaning, alignment, and 2D classification in CryoSPARC, 78,882 segments were selected for further 3D classification. All these analyzed segments adopted a 13-protofilament arrangement. The discarded 79,226 segments were due to low-quality picks, poorly aligned segments, not due to a distinct lattice-state assignment. Of the 78,882 classified segments, 48,459 (61.4% of classified segments, 30.6% of initial segments) were assigned the expanded conformation, and 30,423 (38.6% of classified segments, 19.2% of initial segments) the compact conformation.

For seam detection, alignment parameters were transferred to FREALIGN v9.11 and processed following the established protocol^87^. The final C1 3D reconstruction without application of pseudo-helical symmetry revealed a clear distinction in the S9-S10 loop that differentiates between α and β tubulin subunits, indicating the success of the seam detection and alignment. The refined particle stack and associated alignment parameters were re-imported back to CryoSPARC to perform per-particle defocus refinement, structural refinement, and subsequent 3D classification to distinguish compact and expanded microtubule lattice conformations. The resulting C1 reconstructions achieved resolutions of 3.9 Å for the compact lattice, and 3.59 Å for the expanded lattice (Fig. S8D).

To evaluate the robustness of lattice-state assignment, we repeated the 3D classification procedure six times using different random seeds, which yielded consistent compact versus expanded classes. 94.8–96.6% of particles were assigned to the same class in each of the other five runs, indicating high reproducibility of classification (Fig. S8). Agreement between runs was determined by direct comparison of particle IDs assigned to each class. Per-particle posterior probabilities and Effective Sample Size values were extracted from CryoSPARC and analyzed to evaluate confidence and stability of classification (Fig. S8B and S8C), showing high confidence in the class separation. Classification was performed using C1 symmetry at 3.6–4.0 Å resolution, allowing the separation of the 81–83 Å axial dimer repeat as an explicit structural discriminator. The resulting refined axial spacings closely match previously reported compact and expanded lattice states of microtubules under defined biochemical conditions. Posterior probabilities clustered near 0 or 1 than populating intermediate values (Fig. S8B), supporting distinct separation of the two classified lattice states, though it does not exclude rare or transient intermediate states below our current detection threshold.

For model building, the initial model was prepared using PDB code 9pnd, fit into the cryo-EM maps using ChimeraX^88^ and further refined by rounds of manual fitting in COOT^89^ with subsequent real-space refinement in PHENIX^90^. The final model for the compact lattice had a MolProbity score of 1.75 with a correlation coefficient CC(mask) of 0.78 and an FSC_model_ (masked) of 0.5 at 4.30 Å and of 0.143 at 3.79 Å. The final model for the expanded lattice had a MolProbity score of 1.65 with a correlation coefficient CC(mask) of 0.77 and an FSC_model_ (masked) of 0.5 at 3.93 Å and of 0.143 at 3.53 Å. As a note, the peptide bonds of P274 in α-tubulin and P272 in β-tubulin showed cis configuration in both cases. Data collection, reconstruction and refinement statistics are given in Table S1. The inter-dimer spacing reported for each lattice state (Fig. 7D) is a relative distance measured from atomic models fit into two independently classified and refined reconstructions, each representing an average over individually aligned segments (48,459 for the expanded class, 30,423 for the compact class). Although the 1.9 Å difference between the two lattice repeats is smaller than the nominal resolution of the maps, this measurement does not rely on resolving two isolated densities separated by 1.9 Å but, it reflects a systematic shift in the axial repeat measured across equivalent tubulin heterodimers within classified reconstructions. Thus, the precision of the lattice repeat estimate can exceed the nominal resolution of an individual map feature, while remaining constrained by alignment accuracy, model fitting, and overall map quality.

To assess microtubule polarity relative to the axon direction, the in-plane rotation angle on each segment was extracted from the CryoSPARC output. Neurite trajectories were independently determined from high-magnification montages (HMMs), yielding the local neurite axis angle in image coordinates. For each microtubule segment, the rotation angle relative to the corresponding neurite axis was plotted, such that a value of 0° indicated alignment with the reference microtubule direction. A normalized angle of ~180° indicated anti-parallel orientation relative to the reference microtubule axis. Intermediate angles reflected deviations from the primary neurite trajectory. Distributions of normalized angles were plotted to evaluate polarity coherence and orientation variability (Fig. S7B).

### Single-particle analysis (SPA) of actin filaments in neurites

Actin and cofilactin filaments were processed from the same micrographs using CryoSPARC (Fig. S9). Initial filament picking was performed using Filament Tracer with an average filament diameter of 70 Å and a particle separation of 35 Å. A total of 238,038 particles were extracted and reduced to 91,179 following manual inspection. Of these, 80,804 particles (box size 600 pixels) were subjected to 2D classification, which resolved classes corresponding to actin filaments, microtubules, and decorated filaments. Extensive 2D classification yielded 6835 undecorated actin filaments and 147 decorated filaments. Representative 2D classes were used as templates for a second round of template-based filament picking. Actin filaments were picked using overlapping boxes with a step size of 70 Å and extracted with a box size of 600 pixels. Decorated filaments were picked using a step size of 105 Å and extracted with a box size of 360 pixels. This step resulted in 99,392 actin particles and 4142 decorated filament particles, which were reduced to 35,558 and 2328 particles, respectively, after further 2D classification. Decorated filaments were identified in 17 micrographs. Ab initio models were generated for each filament type prior to helical refinement in CryoSPARC (workflow in Fig. S9). For decorated filaments, the parameters “real space parametrization” and “resolution to begin searches for local symmetry” were set to 9.5 Å and 9 Å, respectively (default values: 8 Å and 6 Å). Initial helical refinement yielded reconstructions at 4.12 Å resolution for actin (rise = 27.51 Å, twist = 166.65°) and 6.58 Å for decorated filaments (rise = 27.38 Å, twist = 162.33°). Local refinement improved the resolutions to 3.88 Å and 5.88 Å, respectively. Particle datasets were subsequently subjected to local CTF refinement and helical symmetry expansion (order = 2) in CryoSPARC, resulting in 71,116 particles for actin and 4656 particles for cofilactin. Final local refinement yielded reconstructions at 3.24 Å for actin and 5.32 Å for decorated. Secondary structure assignment and backbone tracing of the decorated actin filaments revealed cofilin as the binding partner, consistent with the available structure a cofilin-actin complex (PDB 6vao) with a correlation coefficient of the fit of 0.97.

For model building of actin, the initial model was prepared using PDB code 8a2t, fit into the cryo-EM map using ChimeraX and further refined by rounds of manual fitting in COOT with subsequent real-space refinement in PHENIX. The protein sequence was adjusted to *Mus musculus* cytoplasmic β-actin (Uniprot P60710) during the refinement process, which is the major neuronal actin isoform together with γ-actin, and they only differ by four amino acids at the N-terminus. The final model had a MolProbity score of 1.32 with a correlation coefficient CC(mask) of 0.87 and an FSC(model, masked) of 0.5 at 3.48 Å and of 0.143 at 3.19 Å. Data collection, reconstruction and refinement statistics are given in Table S1.

### Statistics and reproducibility

All experiments and observations were conducted and analyzed using at least three biologically independent replicates. Raw data calculations were performed in Microsoft Excel, and the final data was plotted using GraphPad PRISM. The specific significance tests used for each comparison are detailed in the respective figure legends.

### Lead contact and data availability

Information and requests for reagents may be addressed to the Lead Contact, Naoko Mizuno (naoko.mizuno@nih.gov). The cryo-EM reconstructions of microtubules were deposited in the Electron Microscopy database (EMDB) under accession codes EMD-76716 [compact, https://www.ebi.ac.uk/pdbe/entry/emdb/EMD-76716] and EMD-76717 [expanded, https://www.ebi.ac.uk/pdbe/entry/emdb/EMD-76717]. The corresponding microtubule coordinates were deposited in the Protein Data Bank (PDB) under PDB 12rt [compact] and PDB 12ru [expanded]. The F-actin reconstruction was deposited under EMD-76718 with coordinates PDB 12rv [β-actin]. The cofilactin reconstruction (cofilin-decorated F-actin) was deposited under EMD-71056. Source data are provided with this paper.

### Reporting summary

Further information on research design is available in the Nature Portfolio Reporting Summary linked to this article.

## Supplementary information

Supplementary Information

Description of Additional Supplementary Files

Supplementary Movie 1

Supplementary Movie 2

Supplementary Movie 3

Supplementary Movie 4

Reporting Summary

Transparent Peer Review file

## Source data

Source Data
