# Supplementary material for: Ghost Neuron preparation unlocks high-resolution structural mapping of intracellular architecture in axons
Source: Nat Commun. 2026 Sep 23;17:11049. doi: 10.1038/s41467-026-77741-4 (PMC13690744; doi:10.1038/s41467-026-77741-4)
Supplement: Supplementary file 1 [file 41467_2026_77741_MOESM1_ESM.pdf]

Ghost Neuron preparation unlocks high-resolution structural mapping of intracellular architecture in axons

Satish Bodakuntla[1], Julia Marelli[1], Victor Vasquez-Montes[1], Simon Glauser[2] ,Christian Biertümpfel[1], and Naoko Mizuno[1,3*]

[1] Laboratory of Structural Cell Biology, National Heart, Lung, and Blood Institute, National Institutes of Health, 50 South Dr., Bethesda, MD, 20892, USA

[2] Eidgenössische Technische Hochschule Zürich, Department of Chemistry and Applied Biosciences, Institute of Pharmaceutical Sciences, Zürich, Switzerland

[3] National Institute of Arthritis and Musculoskeletal and Skin Diseases, National Institutes of Health, 50 South Dr., Bethesda, MD, 20892, USA

* correspondence

Naoko Mizuno

naoko.mizuno@nih.gov

Supplementary Figures

Bodakuntla *et al* Fig. S1

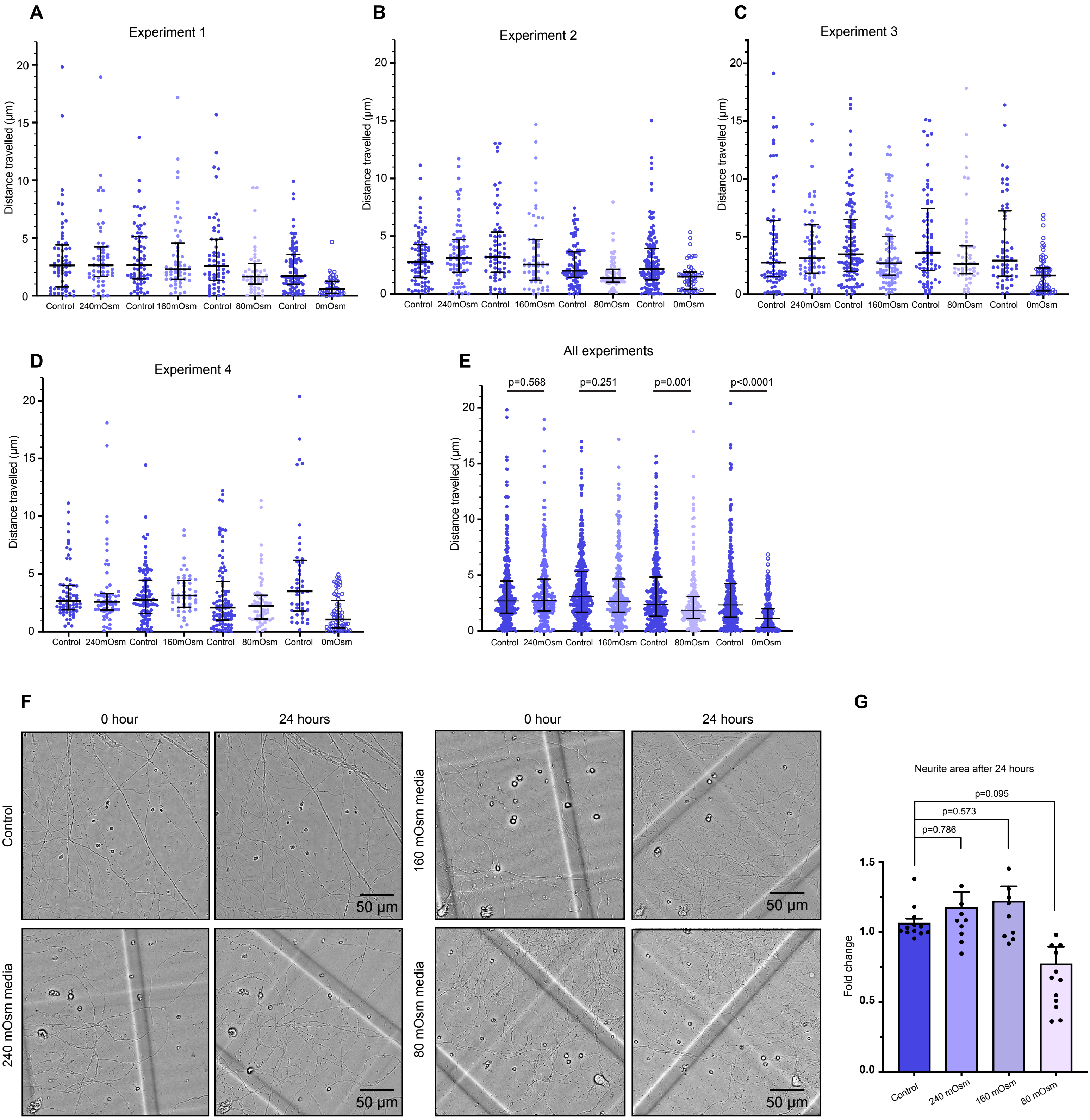

Figure S1: Assessment of neuronal vitality under hypotonic conditions. (A-D) Mitochondria motility in neurons exposed to different osmolarities. The overall distance travelled by mitochondria is shown as scatter dot plots, with horizontal lines representing the median of the population. (E) Data from all the experiments were pooled and shown as a single scatter plot. Significance was tested using two-tailed Student's t-test. (F) Representative DIC images of thalamus explants exposed to varying osmolarities, acquired at 0 and 24 hours post-treatment. (G) Quantification of the neurite area fraction at 0 and 24 hours under the indicated hypotonic conditions. Data are presented as bar graphs (mean ± SEM) along with their corresponding individual data points. Each dot represents the fold change relative to the respective 0-hour baseline. Significance was evaluated using one-way ANOVA with Holm-Šídák's multiple comparisons test.

Bodakuntla *et al* Fig. S2

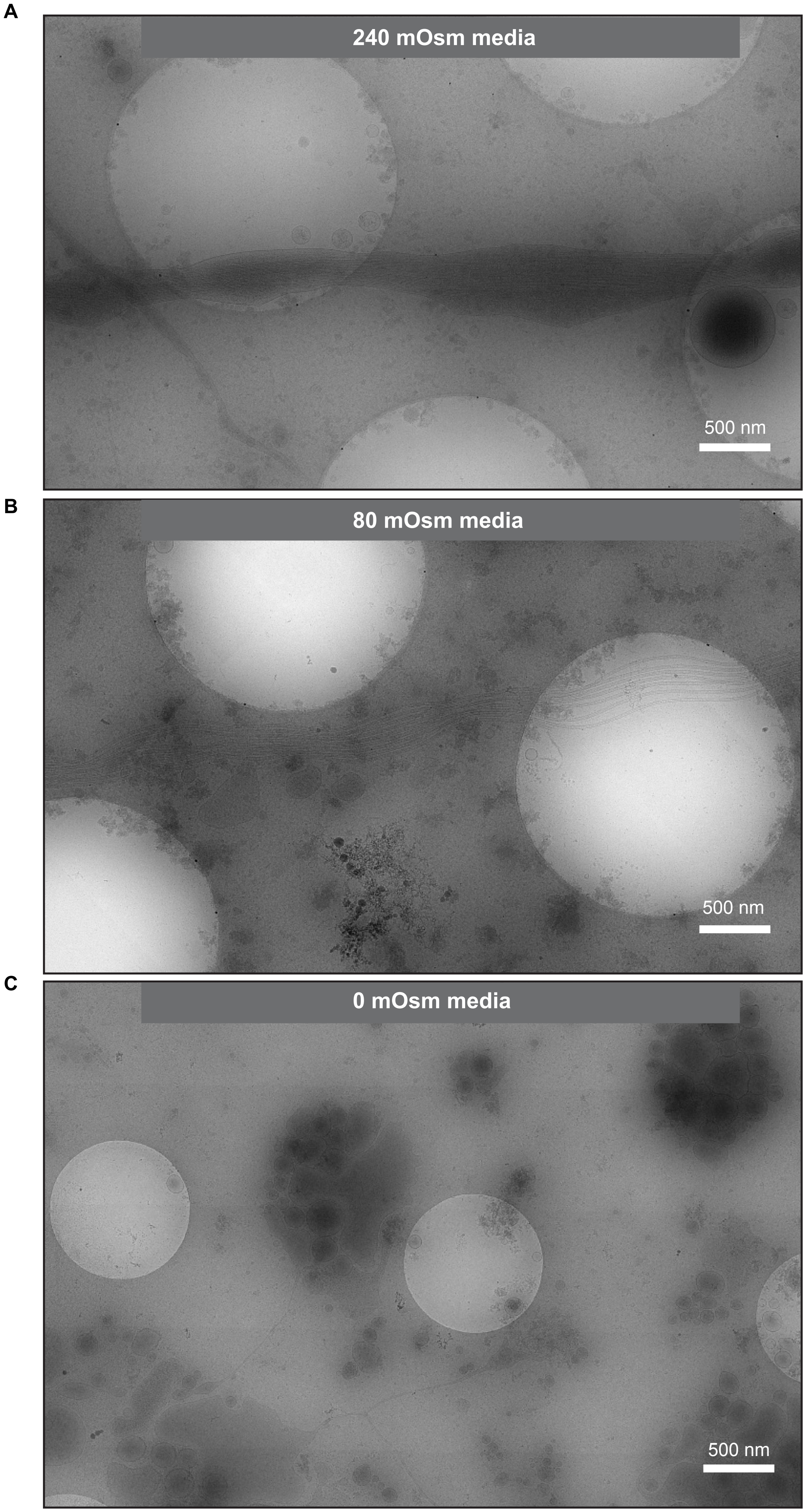

Figure S2: *Ghost Neurons* under varying osmotic conditions observed by cryo-EM. (A) High-magnification montage of a DIV8 thalamus explant axon at 240 mOsm, showing an intact plasma membrane after cryo-EM sample preparation. (B) Membranes are fully disrupted after incubation at 80 mOsm and blotting. (C) Exposure to 0 mOsm hypotonic media and blotting caused pronounced fragmentation under cryo-EM.

Bodakuntla *et al* Fig. S3

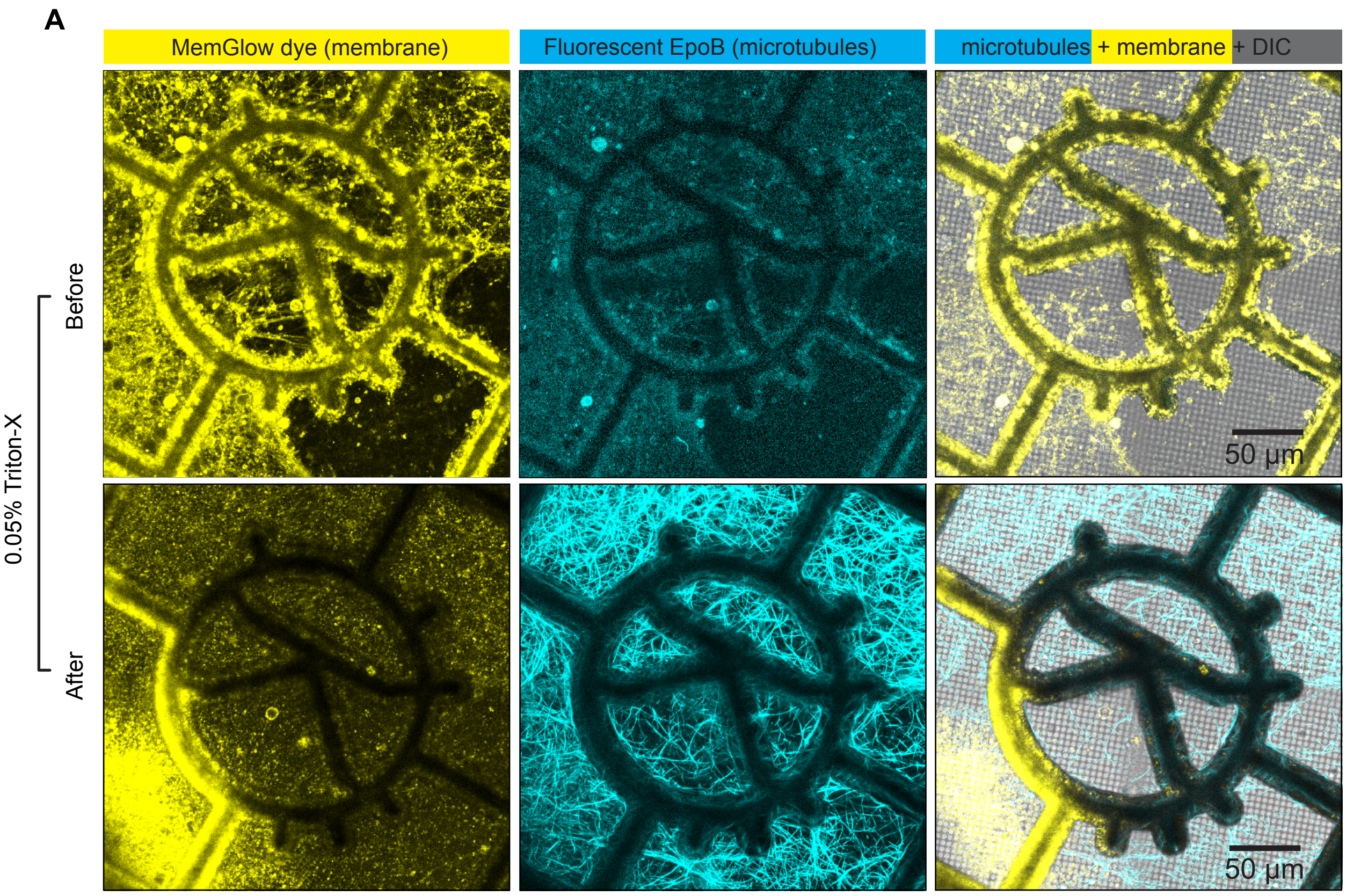

Figure S3: Control experiment assessing tubulin staining before and after detergent permeabilization. Thalamus explants stained with a membrane marker and fluorescently-tagged EpoB were recorded before and after addition of 0.05% (v/v) Triton X-100. Tubular staining was observed after permeabilization of the neuronal membranes by detergent treatment. The corresponding video for this experiment is shown in Movie 3.

Bodakuntla *et al* Fig. S4

A

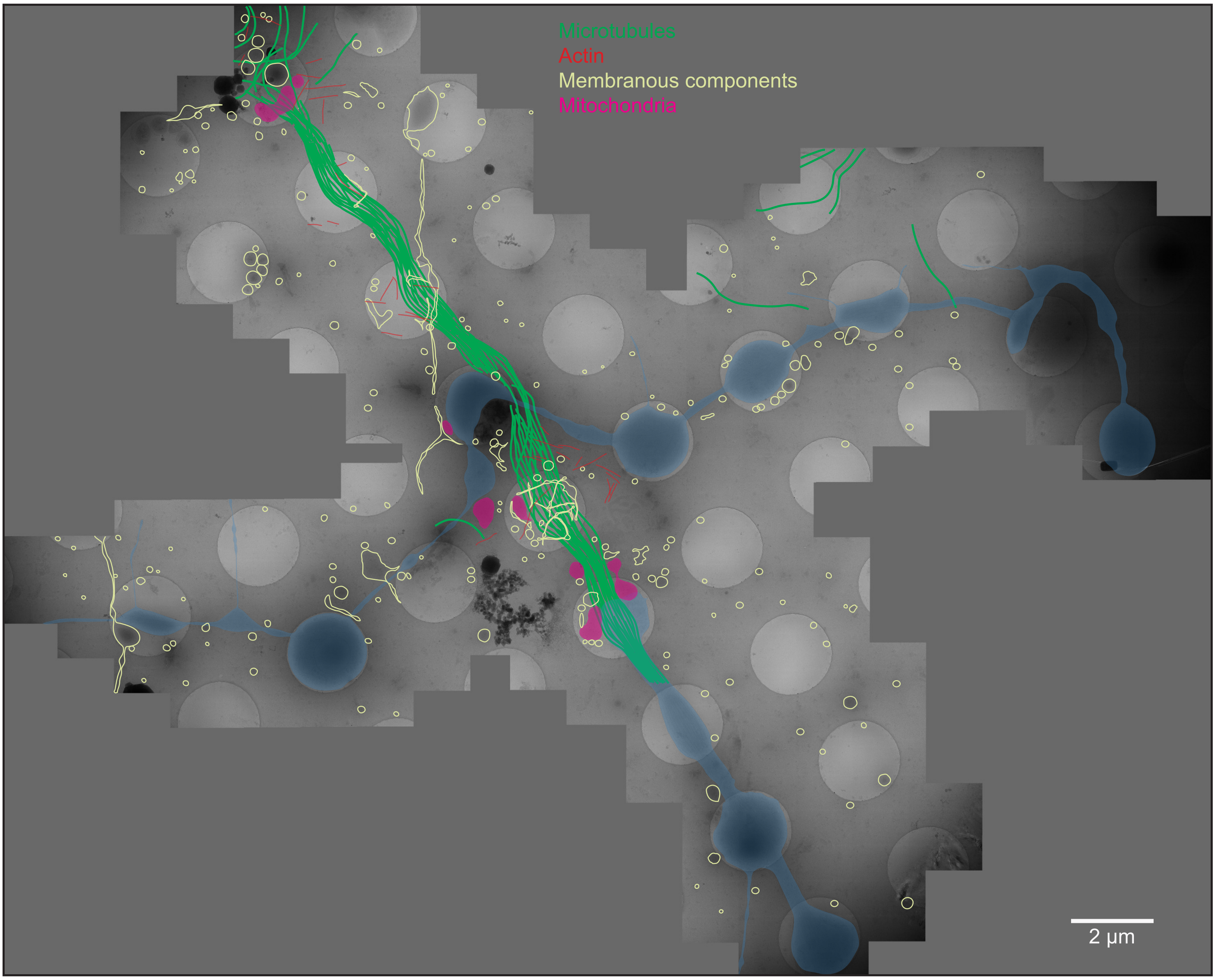

B

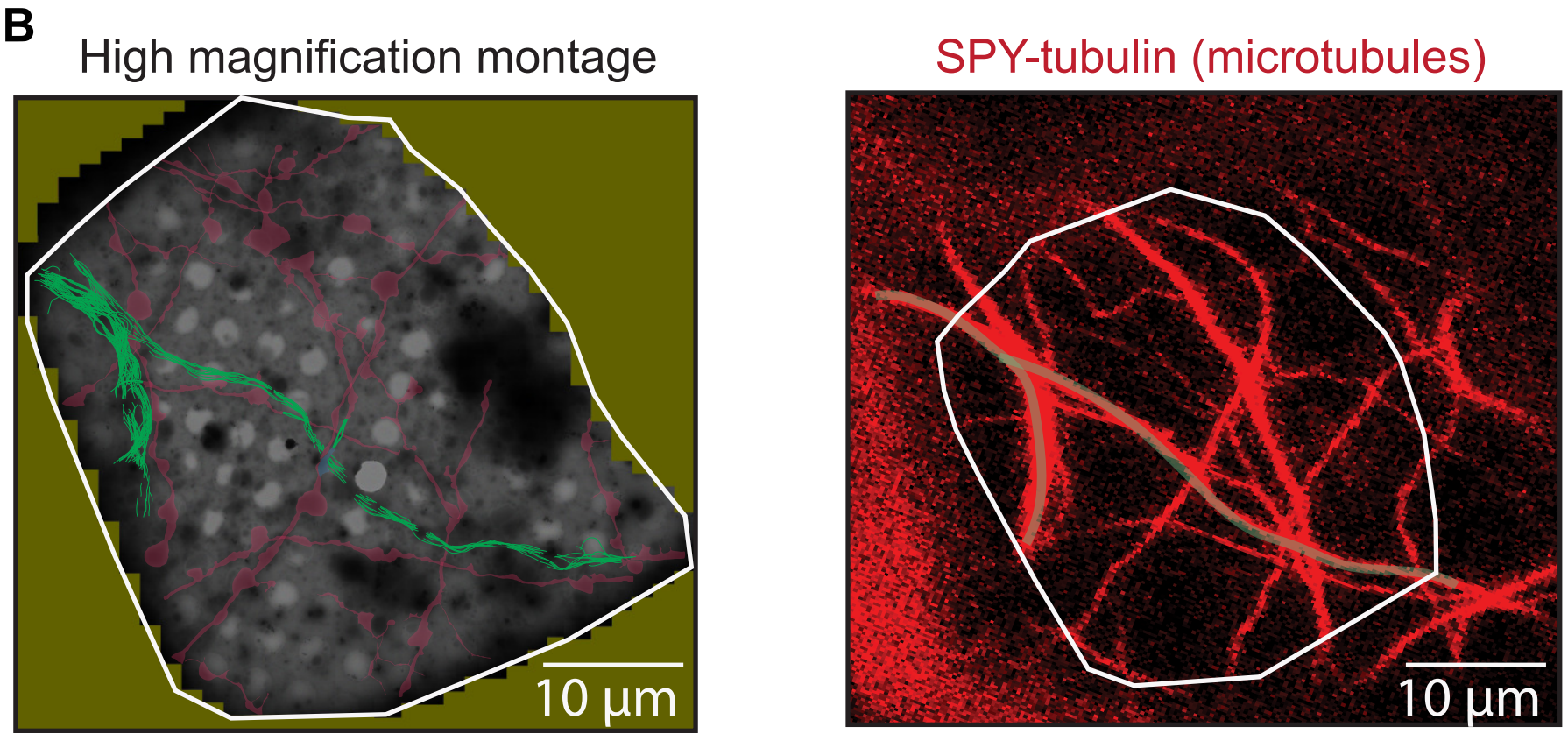

Figure S4: Cryo-EM segmentation of *Ghost Neurons* and trajectory correlation with live cells. (A) Representative image of a typical *Ghost Neuron*, segmented to highlight membranes, mitochondria and cytoskeletal components. Microtubule bundles continue smoothly in the *Ghost Neuron*. (B) Co-alignment of SPY-tubulin (red) labeled microtubules in live imaging (right) with the corresponding cryo-EM montage (left). The white border polygons approximate the corresponding regions in live imaging and cryo-EM high-magnification montage. The green lines trace microtubules that are without plasma membrane. Pink tracings show intact neurites. The overall path of the neurites remains unchanged during the *Ghost Neuron* preparation.

Bodakuntla *et al* Fig. S5

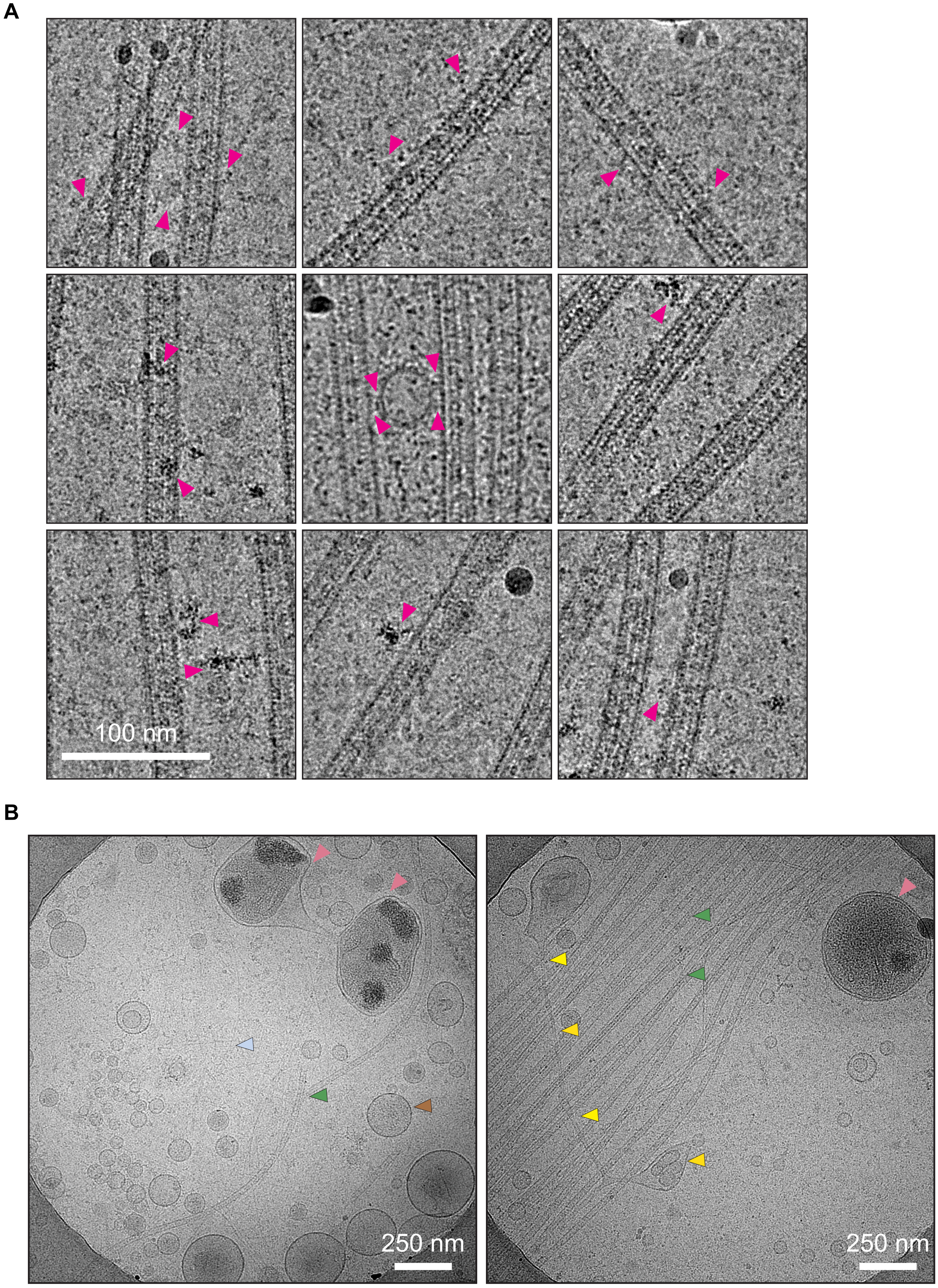

Figure S5: Cryo-EM micrographs show macromolecular assemblies and organelle morphologies. (A) Cryo-EM micrographs showing microtubules associated with various additional densities as highlighted by the magenta arrow heads. (B) Snapshots acquired from a *Ghost Neuron* showing the intact nature of various cellular activities including mitochondria fission (pink arrows, on the left), ER entanglement (yellow arrows) with microtubules (green arrows), vesicles (brown arrow) and actin mesh (light blue arrow).

Bodakuntla et al Fig. S6

A. Undecorated F-actins

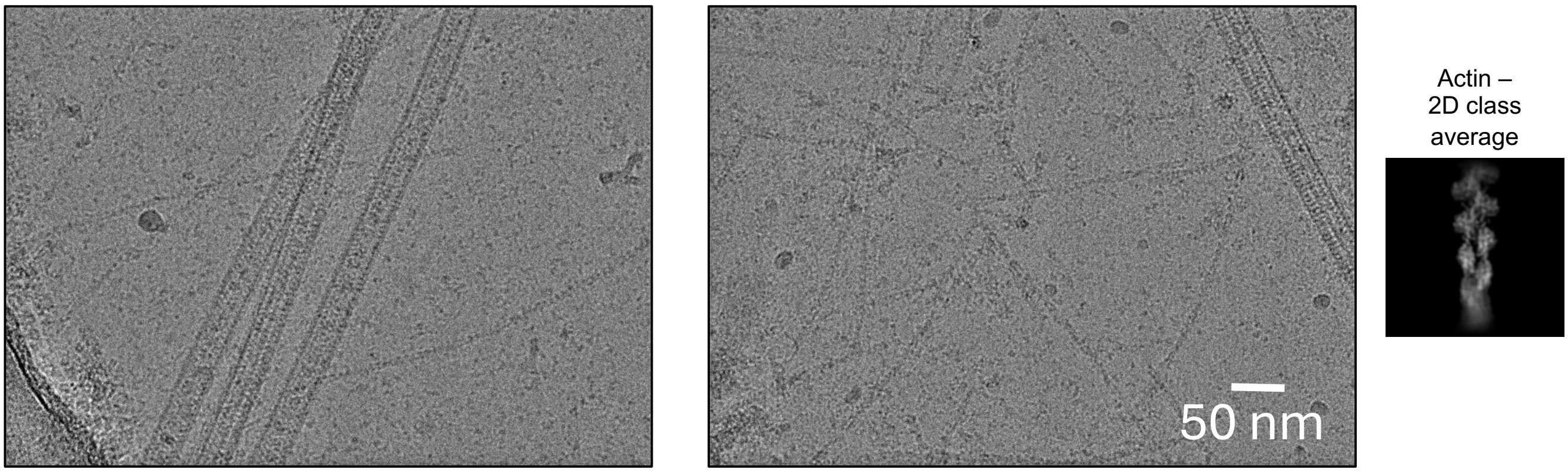

B. Cofilin decorated F-actins

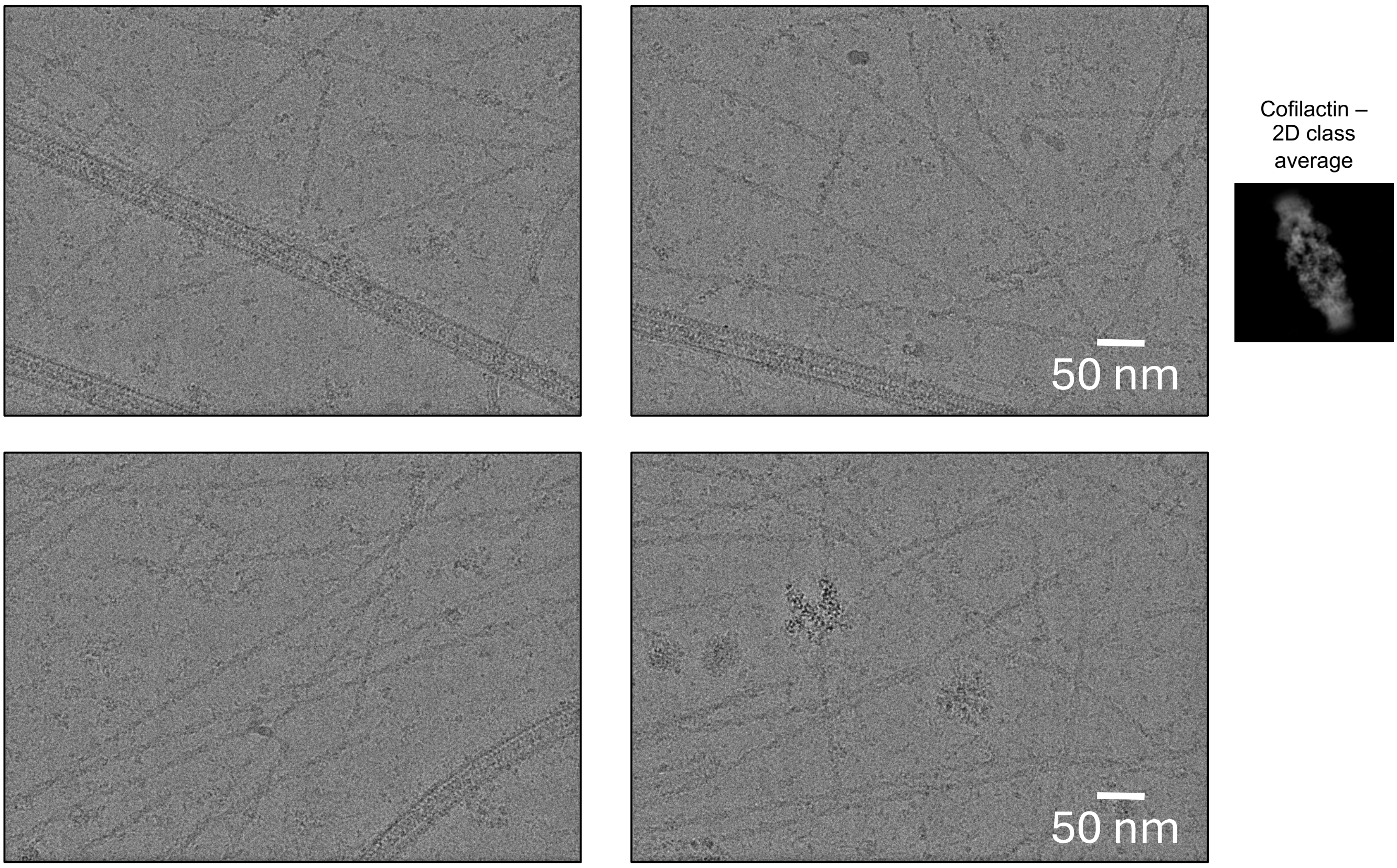

Figure S6: Representative micrographs showing F-actin and Cofilin-decorated F-actin. Respective 2D class averages are shown on the right.

Bodakuntla *et al* Fig. S7

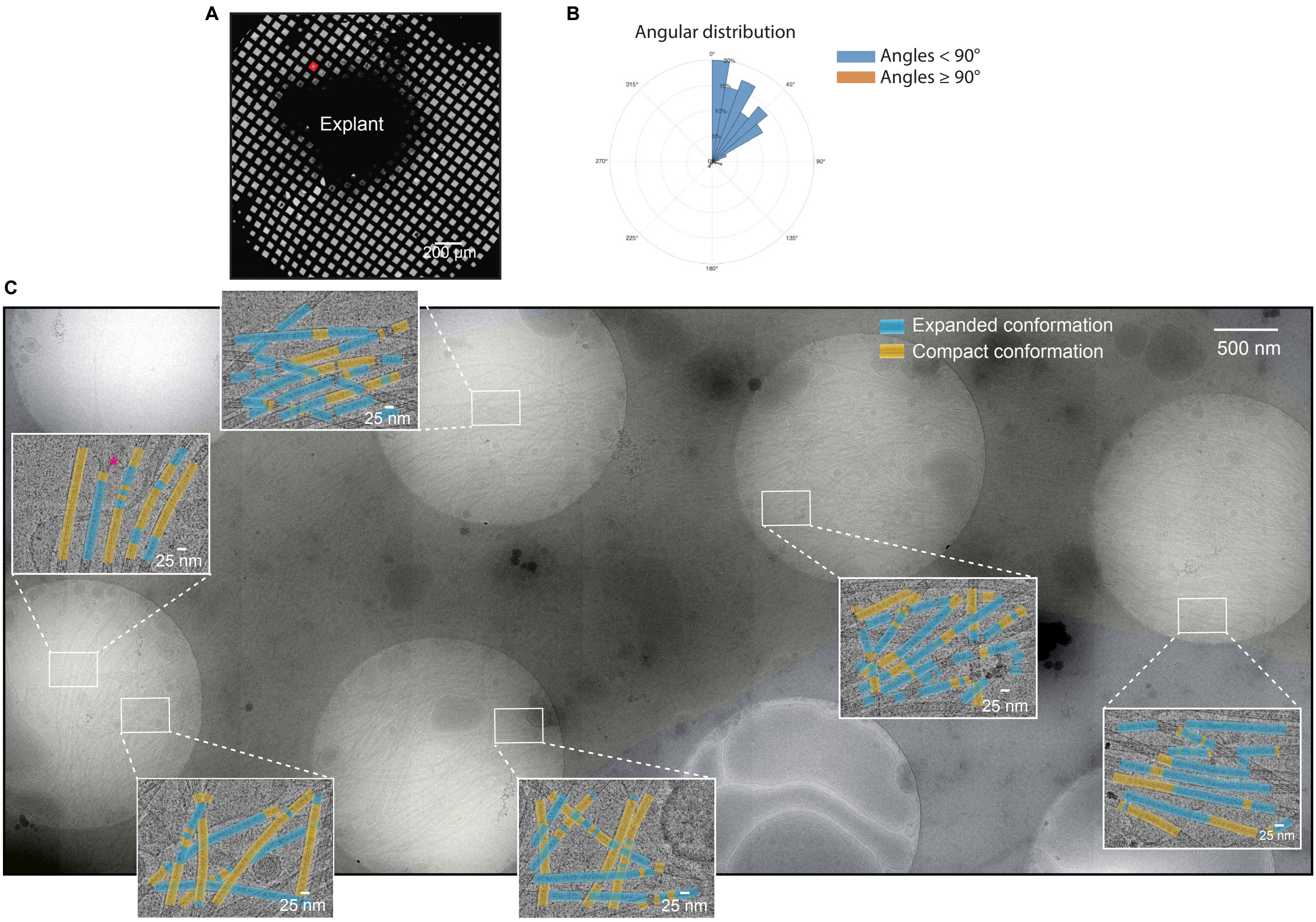

Figure S7: Mapping of microtubule lattice states within neurites. (A) Low-magnification overview of an thalamus explant with a red box highlighting the region where a high-magnification neurite montage was acquired. (B) Angular distribution of microtubules relative to neurite direction, showing that the majority of microtubules align parallel to each other. (C) Contextual mapping of expanded (blue) and compact (orange) microtubule conformations, back-plotted onto high-magnification cryo-EM montages of a *Ghost Neuron*. Segments without a colored assignment were not used for the final analysis do not represent another, or an intermediate lattice state. The leftmost inset highlights a representative microtubule plus end (asterisk), with an expanded lattice patch at the tip followed by compact segments toward the shaft.

Bodakuntla *et al* Fig. S8

A

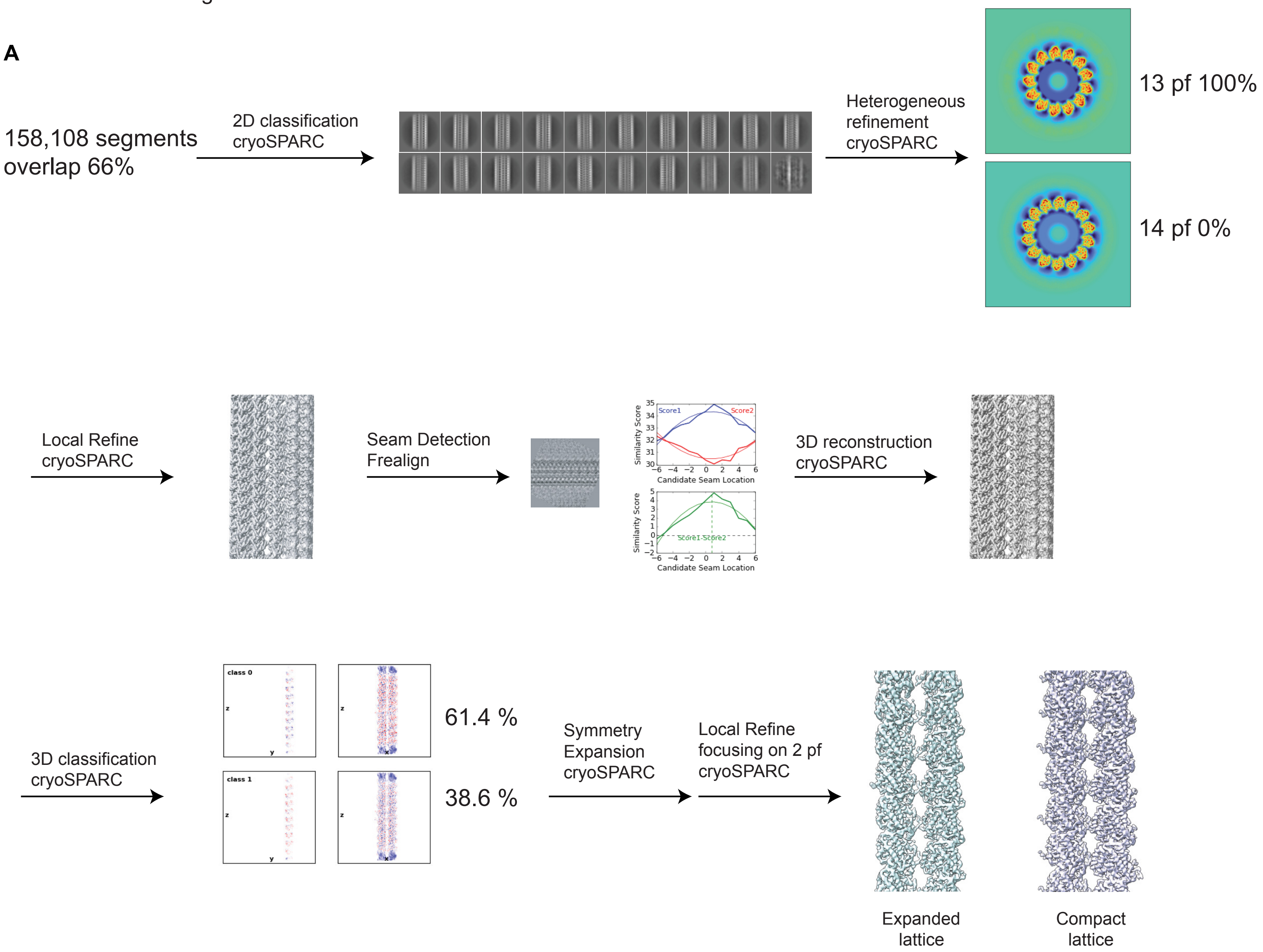

B

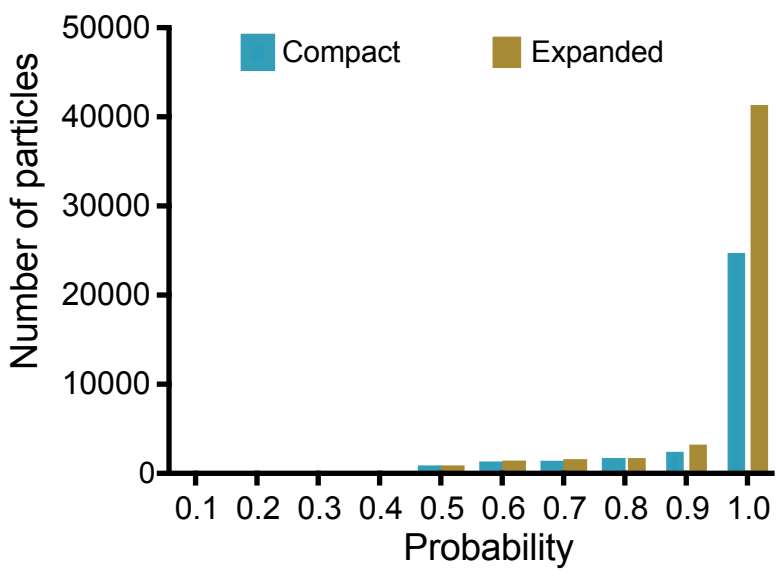

C

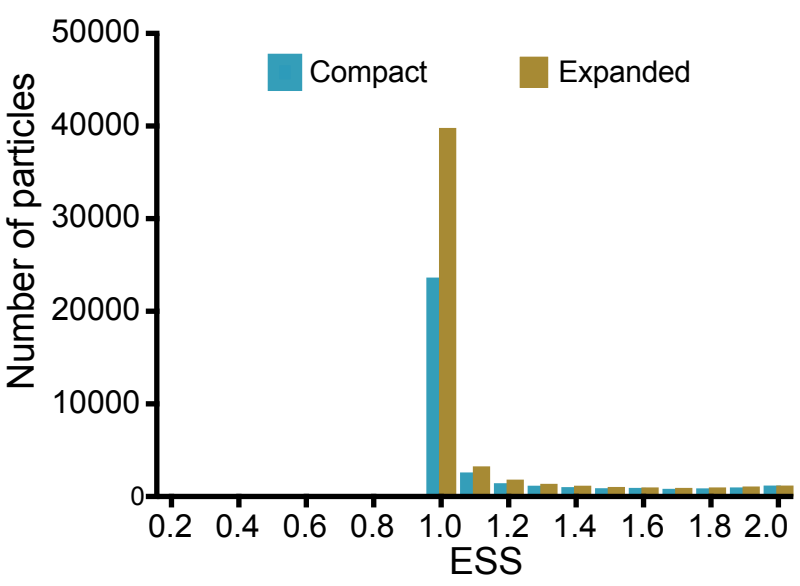

D

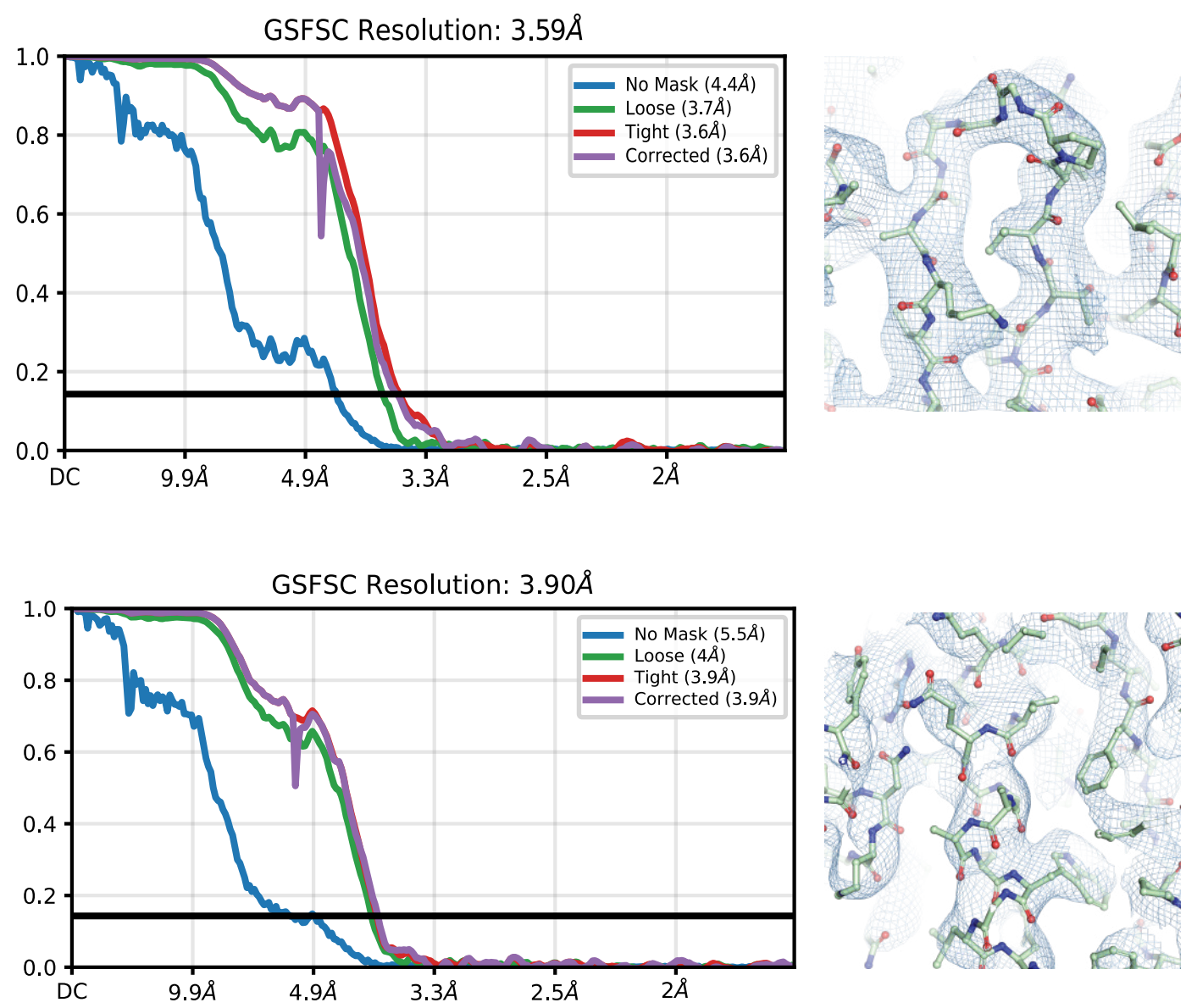

E

	Total	Expanded	Compact	Overlap (compact)	Percentage
Run 1	78,882	48,459	30,423		
Run 2	78,882	48,764	30,118	28,847	94.83
Run 3	78,882	48,085	30,797	29,146	95.81
Run 4	78,882	48,362	30,520	29,036	95.45
Run 5	78,882	47,916	30,966	29,377	96.57
Run 6	78,882	48,351	30,531	29,079	95.59

Figure S8: SPA workflow for the analysis of microtubules from the micrographs acquired from *Ghost Neurons*. (A) Workflow for microtubule reconstruction and classification. Segments were subjected to local refinement in CryoSPARC, followed by seam detection using FREALIGN and 3D reconstruction. Subsequent 3D classification separated particles into two major conformational classes, corresponding to expanded and compact lattice states. Focused refinement on two protofilaments further improved structural resolution. Representative atomic models for each lattice state are shown. (B) Distribution of per-particle classification probabilities for compact (blue) and expanded (brown) classes. The majority of particles are assigned with high confidence (probabilities approaching 1), indicating well-separated structural states. (C) Distribution of effective sample size (ESS) values for both classes. High ESS values across particles indicate stable and non-degenerate classification. (D) Gold-standard Fourier shell correlation (FSC) curves for the reconstructed microtubule lattices, yielding resolutions of 3.59 Å (top) and 3.9 Å (bottom) after masking and correction. (E) Reproducibility of classification across independent runs. Six independent 3D classification runs yield consistent particle distributions between expanded and compact classes, with high overlap (94.8–96.6%) in particle assignment, demonstrating robustness of lattice-state classification.

Bodakuntla *et al* Fig. S9

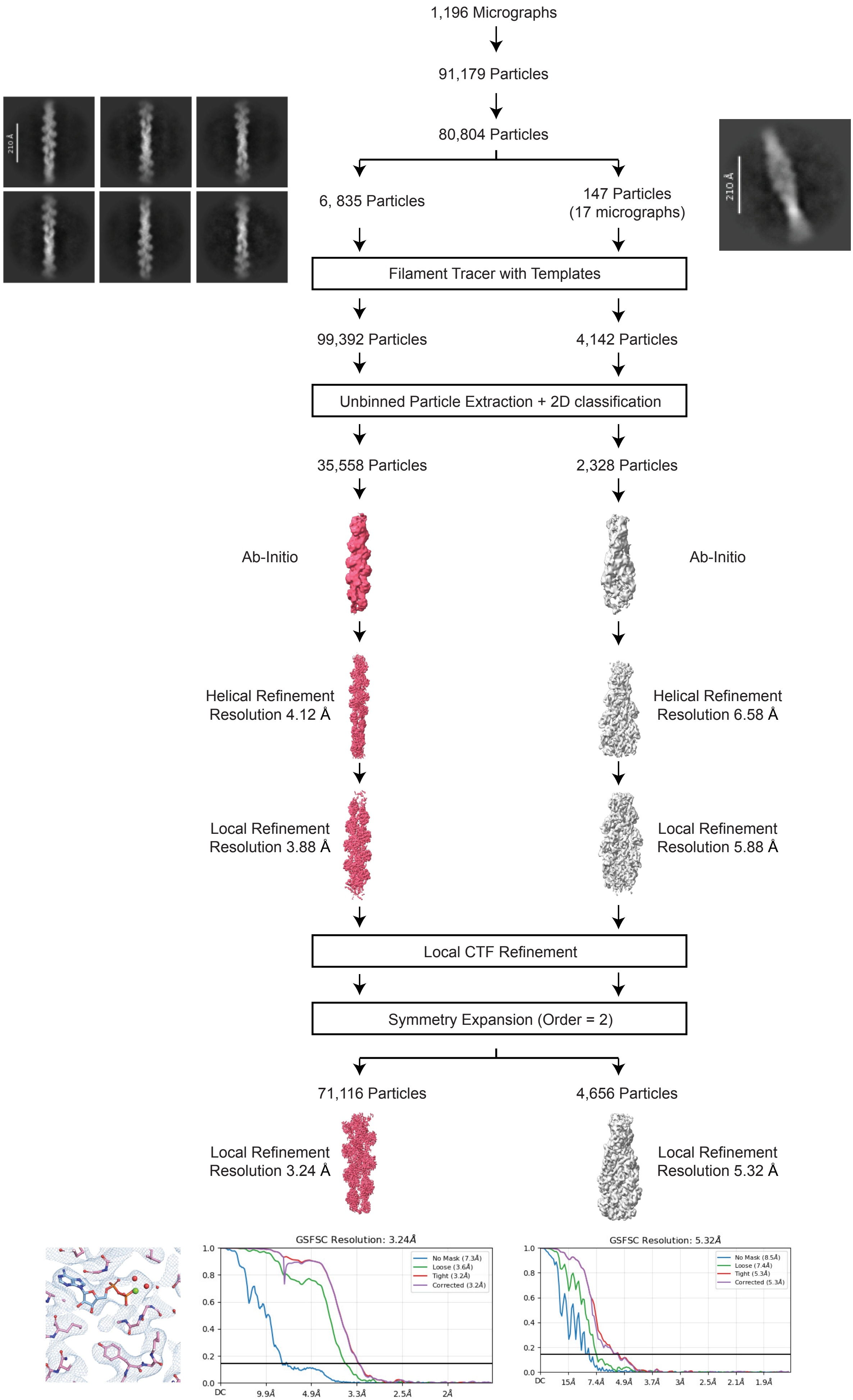

Figure S9: SPA workflow for the analysis of actin and cofilactin from the micrographs acquired from *Ghost Neurons*. Filament segments were extracted from cryo-EM micrographs and subjected to template-based tracing, followed by 2D classification and *ab initio* reconstruction. Two particle-populations were identified, corresponding to undecorated actin filaments and cofilin-decorated filaments. Helical refinement and subsequent local refinement improved map quality for both datasets. Local CTF refinement and symmetry expansion further enhanced resolution. Final reconstructions yielded structures of actin filaments and cofilin-decorated filaments. Gold-standard Fourier shell correlation (FSC) curves for the final reconstructions are shown, indicating resolutions of 3.24 Å for actin filaments and 5.32 Å for cofilin-decorated filaments.

Table S1: Data collection, reconstruction and refinement statistics

**Data Set**	**EMD-76716 PDB 12rt *C*ryo-EM structure of axonal microtubules from ghost neurons (compact lattice)**	**EMD-76717 PDB 12ru *C*ryo-EM structure of axonal microtubules from ghost neurons (expanded lattice)**	**EMD-76718 PDB 12rv *C*ryo-EM structure of axonal F-actin from ghost neurons**	**EMD-71056 *C*ryo-EM structure of axonal cofilactin filaments from ghost neurons**
**Data collection statistics**				
Microscope	TFS Titan Krios G4	TFS Titan Krios G4	TFS Titan Krios G4	TFS Titan Krios G4
Energy filter	BioContinuum GIF	BioContinuum GIF	BioContinuum GIF	BioContinuum GIF
Detector	Gatan K3	Gatan K3	Gatan K3	Gatan K3
Grid type	Quantifoil Au 200 R2/4	Quantifoil Au 200 R2/4	Quantifoil Au 200 R2/4	Quantifoil Au 200 R2/4
Magnification	105000	105000	105000	105000
Voltage (kV)	300	300	300	300
Electron dose (e^-/Å^2)	52.2	52.2	54.52	54.52
Defocus range (μm)	-0.8 to -3.0	-1.1 to -3.0	-0.8 to -3.0	-0.8 to -3.0
Number of movie frames	40	40	40	40
Pixel size (Å)	0.412	0.412	0.412	0.412
Number of micrographs	4172	4172	1196	17
Number of particles (total)	158108	158108	99392	4142
Number of particles (final)	30423	48459	35558	2328
Helical twist (˚)	-27.64	-27.64	166.647	162.334
Helical rise (Å)	9.695	9.695	27.514	27.383
FSC threshold	0.143	0.143	0.143	0.143
Global resolution (Å)	3.90	3.59	3.24	5.32
**Refinement statistics**				
Particles used for final map	30423	48459	35558	
Map sharpening B factor (Å^2)	-67.0	-57.2	-64.9	
Number of Residues				
Proteins	1734	1734	1865	
Nucleotides (ADP)			5	
Nucleotides (GDP/GTP)	2/2	2/2	n/a	
Number of Atoms				
Proteins	13617	13617	14550	
Nucleotides (GDP/GTP)	88	88	103	
Metal ions (Mg^{2+})	4	2	5	
Water	0	0	11	
B-factors				
Protein	134.14	132.88	99.88	
Nucleotides (ADP)			93.22	
Nucleotides (GDP/GTP)	122.05	117.98	n/a	
Metal ions (Mg^{2+})	137.62	140.44	102.66	
Water			90.06	
R.m.s deviations				
Bond lengths (Å)	0.003	0.003	0.002	
Bond angles (˚)	0.747	0.728	0.560	
Validation				
MolProbity score	1.75	1.65	1.32	
Clashscore	17.67	13.92	5.84	
Rotamer outliers (%)	0.68	0.81	0.70	
CaBLAM outliers (%)	0.64	0.87	0.43	
Cβ outliers (%)	0.00	0.00	0.00	
CC(mask)	0.78	0.77	0.87	
FSC_{model}(0/0.143/0.5)	3.35/3.79/4.30	3.24/3.53/3.93	3.08/3.19/3.48	
Ramachandran Plot				
Favored (%)	98.20	98.09	98.80	
Allowed (%)	1.80	1.91	1.20	
Outliers (%)	0.00	0.00	0.00	
