# Supplementary material for: Ghost Neuron preparation unlocks high-resolution structural mapping of intracellular architecture in axons
Source: Nat Commun. 2026 Sep 23;17:11049. doi: 10.1038/s41467-026-77741-4 (PMC13690744; doi:10.1038/s41467-026-77741-4)
Supplement: Supplementary file 2 [file 41467_2026_77741_MOESM2_ESM.pdf]

Description of Additional Supplementary Files

Supplementary Movie 1: Thalamus explants grown on glass dishes and exposed to different hypotonic conditions. The neurons were stained with MemGlow 488 (plasma membrane, green) and recorded before and after changing osmolarity as indicated.

Supplementary Movie 2: Mitochondria staining of thalamus explants exposed to hypotonic conditions. Neurons were stained with MemGlow 488 (plasma membrane, blue) and MitoTracker Red CMXRos (mitochondria, magenta) and recorded before and after changing to different hypotonic conditions as indicated. Scale bar: 20 μm.

Supplementary Movie 3: Microtubules can be stained in thalamus axons after mild detergent treatment. The neurons were stained with MemGlow 488 (plasma membrane, yellow) and fluorescently-conjugated EpoB (cyan) for ten minutes and recorded before and after the addition of 0.05% Triton X-100. A tubular staining was observed after permeabilization of the neuronal plasma membrane. Scale bar: 50 μm.

Supplementary Movie 4: Thalamus explants grown on EM grids and exposed to different hypotonic conditions. The neurons were stained with MemGlow 488 (plasma membrane, yellow) and fluorescently-labelled EpoB (cyan) and recorded in z-direction to reveal microtubule signals inside cells at osmolarity of 160 mOsm and 80 mOsm post blotting. There was no microtubule staining visible in neurons in 320 mOsm (control) or 240 mOsm media. Scale bar: 50 μm.
