# Supplementary material for: Ghost Neuron preparation unlocks high-resolution structural mapping of intracellular architecture in axons
Source: Nat Commun. 2026 Sep 23;17:11049. doi: 10.1038/s41467-026-77741-4 (PMC13690744; doi:10.1038/s41467-026-77741-4)
Supplement: Supplementary file 3 [file 41467_2026_77741_MOESM7_ESM.pdf]

Corresponding author(s): Naoko Mizuno
Last updated by author(s): Jun 30, 2026

Reporting Summary

Nature Portfolio wishes to improve the reproducibility of the work that we publish. This form provides structure for consistency and transparency in reporting. For further information on Nature Portfolio policies, see our Editorial Policies and the Editorial Policy Checklist.

Statistics

For all statistical analyses, confirm that the following items are present in the figure legend, table legend, main text, or Methods section.

n/a	Confirmed	
☐	☒	The exact sample size (n) for each experimental group/condition, given as a discrete number and unit of measurement
☐	☒	A statement on whether measurements were taken from distinct samples or whether the same sample was measured repeatedly
☐	☒	The statistical test(s) used AND whether they are one- or two-sided<br>*Only common tests should be described solely by name; describe more complex techniques in the Methods section.*
☒	☐	A description of all covariates tested
☐	☒	A description of any assumptions or corrections, such as tests of normality and adjustment for multiple comparisons
☐	☒	A full description of the statistical parameters including central tendency (e.g. means) or other basic estimates (e.g. regression coefficient) AND variation (e.g. standard deviation) or associated estimates of uncertainty (e.g. confidence intervals)
☐	☒	For null hypothesis testing, the test statistic (e.g. F, t, r) with confidence intervals, effect sizes, degrees of freedom and P value noted<br>*Give P values as exact values whenever suitable.*
☒	☐	For Bayesian analysis, information on the choice of priors and Markov chain Monte Carlo settings
☒	☐	For hierarchical and complex designs, identification of the appropriate level for tests and full reporting of outcomes
☒	☐	Estimates of effect sizes (e.g. Cohen's d, Pearson's r), indicating how they were calculated

Our web collection on statistics for biologists contains articles on many of the points above.

Software and code

Policy information about availability of computer code

Data collection	For EM-data collection of SPA and tomograms, Serial-EM application (https://bio3d.colorado.edu/SerialEM/) was used. For live imaging, Nikon software NIS-Elements Version 6 were used.
Data analysis	Analysis was performed using ImageJ or Fiji (https://imagej.net/software/fiji/downloads) with plugins described in the methods section.<br><br>For electron microscopy – RELION (https://www2.mrc-lmb.cam.ac.uk/relion/), Frealign (grigoriefflab.janelia.org/frealign), customized pipeline for microtubule analysis (PMID: 26424086), CryoSPARC (https://cryosparc.com/) was used.<br><br>Prism 7 and Microsoft Excel 15.35 were used for statistical analysis and graphical data presentation (https://www.graphpad.com/).

For manuscripts utilizing custom algorithms or software that are central to the research but not yet described in published literature, software must be made available to editors and reviewers. We strongly encourage code deposition in a community repository (e.g. GitHub). See the Nature Portfolio guidelines for submitting code & software for further information.

Data

Policy information about availability of data

All manuscripts must include a data availability statement. This statement should provide the following information, where applicable:

- Accession codes, unique identifiers, or web links for publicly available datasets
- A description of any restrictions on data availability
- For clinical datasets or third party data, please ensure that the statement adheres to our policy

The SPA reconstruction of microtubules analyzed from the Ghost Neuron is available under accession codes EMD-76716, PDB 12rt (axonal microtubules, compact lattice), EMD-76717, PDB 12ru (axonal microtubules, Expanded lattice), EMD-76718 PDB 12rv (axonal F-actin, undecorated) and EMD-71056 (axonal cofilactin filaments).

Research involving human participants, their data, or biological material

Policy information about studies with human participants or human data. See also policy information about sex, gender (identity/presentation), and sexual orientation and race, ethnicity and racism.

Reporting on sex and gender	N/A - Human participants, their data, or biological material was not used.
Reporting on race, ethnicity, or other socially relevant groupings	N/A - Human participants, their data, or biological material was not used.
Population characteristics	N/A - Human participants, their data, or biological material was not used.
Recruitment	N/A - Human participants, their data, or biological material was not used.
Ethics oversight	N/A - Human participants, their data, or biological material was not used.

Note that full information on the approval of the study protocol must also be provided in the manuscript.

Field-specific reporting

Please select the one below that is the best fit for your research. If you are not sure, read the appropriate sections before making your selection.

[x] Life sciences [ ] Behavioural & social sciences [ ] Ecological, evolutionary & environmental sciences

For a reference copy of the document with all sections, see nature.com/documents/nr-reporting-summary-flat.pdf

Life sciences study design

All studies must disclose on these points even when the disclosure is negative.

Sample size	All experiments for the purpose of testing statistical significance and to draw conclusion have been performed at least in three biological independent replicates. Multiple neurites, fields of view, grid regions, or particles were analyzed from these samples as described in the figure legends and Methods. This was considered sufficient for method-development and validation experiments focused on reproducibility of preparation, imaging quality, and structural analysis.
Data exclusions	None
Replication	ll attempts of replication of the biological experiments were successful. For quantification triplicates datasets (biologically and technical replicates) are collected.
Randomization	Randomization was not performed
Blinding	Not performed. Blinding was not generally feasible because the experimental conditions were visually apparent from the samples.

Reporting for specific materials, systems and methods

We require information from authors about some types of materials, experimental systems and methods used in many studies. Here, indicate whether each material, system or method listed is relevant to your study. If you are not sure if a list item applies to your research, read the appropriate section before selecting a response.

Materials & experimental systems

n/a	Involved in the study
☒	☐ Antibodies
☒	☐ Eukaryotic cell lines
☒	☐ Palaeontology and archaeology
☐	☒ Animals and other organisms
☒	☐ Clinical data
☒	☐ Dual use research of concern
☒	☐ Plants

Methods

n/a	Involved in the study
☒	☐ ChIP-seq
☒	☐ Flow cytometry
☒	☐ MRI-based neuroimaging

Animals and other research organisms

Policy information about studies involving animals; ARRIVE guidelines recommended for reporting animal research, and Sex and Gender in Research

Laboratory animals	Cultures were performed from embryos (at E15.5) of CD-1 mice (Mus musculus, outbred CD-1/ICR genetic background , Charles River Laboratories). Timed-pregnant mice were purchased and used for embryo collection immediately upon arrival. Animals were not bred, maintained, or subjected to experimental housing conditions in our facility before dissection
Wild animals	Did not use any wild animals
Reporting on sex	Cultures were performed from embryos (at E15.5) of CD-1 mice (Charles River Laboratories).
Field-collected samples	None
Ethics oversight	All animal procedures were approved by the Animal Care and Use Committee (ACUC) of the National Heart, Lung, and Blood Institute (NHLBI) animal protocol H-0331 in accordance with NIH research guidelines for the care and use of laboratory animals.

Note that full information on the approval of the study protocol must also be provided in the manuscript.

Plants

Seed stocks	Not applicable
Novel plant genotypes	Not applicable
Authentication	Not applicable
