# Supplementary material for: Ghost Neuron preparation unlocks high-resolution structural mapping of intracellular architecture in axons
Source: Nat Commun. 2026 Sep 23;17:11049. doi: 10.1038/s41467-026-77741-4 (PMC13690744; doi:10.1038/s41467-026-77741-4)
Supplement: Supplementary file 4 [file 41467_2026_77741_MOESM8_ESM.pdf]

nature portfolio

Peer Review File

Ghost Neuron preparation unlocks high-resolution structural mapping of intracellular architecture in axons

Corresponding Author: Dr Naoko Mizuno

This file contains all reviewer reports in order by version, followed by all author rebuttals in order by version.

Version 1:

Reviewer comments:

Reviewer #1

(Remarks to the Author)
This manuscript by Bodadkuntla et al. introduces a procedure for creating "ghost" neurons by employing a low osmolarity treatment combined with mechanical shearing via paper blotting. This method effectively removes the plasma membrane along neuronal processes, primarily axons, which enhances the contrast for subsequent cryo-electron tomography (cryo-ET) examination. The authors apply this technique to study actin and microtubules within axons, utilizing single-particle averaging to reveal the presence of cofilin-covered actin filaments and distinct segments of expanded and compacted lattices along individual axonal microtubules. While the manuscript is of high quality and successfully demonstrates the utility of this new procedure, several concerns must be addressed before it can be recommended for publication.

Incomplete Context and Discussion of Results
A central argument of the manuscript is the novelty of the procedure and the applications it enables. However, it omits key studies that are essential for providing a comprehensive view of the current state of the art. These should be cited in the Introduction and discussed in subsequent sections.
- The Introduction suggests that cryo-EM along neuronal processes lacks sufficient contrast, thereby justifying the development of the ghost neuron procedure. However, a significant body of existing work has successfully performed cryo-EM along neurites, including cryo-ET, single-particle averaging, and structural assessment of cytoskeletal assemblies (Petrovic et al., Curr Op Neurobiol 2025). This includes research from the Baumeister/Lučić labs, the Carter lab (Foster et al., JCB 2022), the Kukulski lab (Hoffman et al., eLife 2021), the Moores lab (Atherton et al., Acta Crystallograph Sect D 2018), Zamponi et al. (Dev Cell 2022), and even the authors' own lab (Nedozralova et al., JCB 2022, which is only cited in the Methods section). These prior studies should be properly acknowledged, and their limitations must be clearly explained to justify the need for the new method.
- In the discussion of plasma membrane removal techniques, the authors mention unroofing (citing Vassilopoulos et al., Nat Comm 2019) and the preparation of red blood cell ghosts. The manuscript fails to include surfactant-induced membrane removal prior to fixation, a method used extensively by the Svitkina lab to visualize cytoskeletal organization, including in neuronal processes (Korobova et al., MBC 2010; Jones et al., JCB 2014; Efimova et al., J Neuro 2017).
- In the Results section, the authors state, “While these lattice states have been characterized in vitro using cryo-EM with defined nucleotide conditions, our data provide the first direct in situ evidence for the presence of these distinct lattice conformations within the native neuronal environment” (lines 182-183). This overlooks the work of de Jager et al. (JCB 2024), which identified expanded and compacted lattices in cells using cryo-ET (even if not in neurons). This study is highly relevant to the assessment of tubulin lattice states - one of the two main applications of the ghost neuron procedure - and should be cited and discussed.
- The Discussion lists the advantages of the ghost neuron procedure (lines 247-258), but it remains unclear how these benefits are unique compared to unroofing or detergent-induced membrane removal in similar culture models.

Manuscript Structure
- The purpose of the "Ghost neuron specification" section (lines 75-89), positioned between the Introduction and Results, is unclear as it appears to summarize the Results. Furthermore, the final paragraph of the Introduction (lines 62-73) repeats arguments from previous paragraphs. It is suggested that both be reworked into a single, concise final paragraph for the Introduction.
- The "Material availability" section (lines 289-291) does not state how the materials can be accessed.

Limitations of the Study
The primary limitation of this procedure, which is only partially addressed, is that it scrambles the organization of submembrane scaffolds. This is particularly significant in neurons, where a periodic skeleton of actin rings connected by spectrin tetramers - known as the membrane-associated skeleton (MPS) - is a fundamental structural component (Xu et al., Science 2013). The MPS has been observed in all neurons and species studied, primarily through optical super-resolution techniques and platinum replica EM after unroofing (reviewed in Leterrier, Curr Op Neurobiol 2021). Notably, no existing cryo-EM/cryo-ET study has successfully visualized the MPS. While a new procedure for improved visualization of axonal architecture is welcome, this method does not address this main shortcoming of existing techniques. The disordering of the submembrane scaffold is acknowledged in the Results when discussing actin organization (lines 148-150) , although the meaning of "approximate localization was maintained" is unclear. To clarify the consequences of this, a brief description of the MPS and key references should be included. The consequence of this disruption, namely the inability to visualize the MPS, should be explicitly stated as a key limitation of the technique.

Experimental Questions
- Actin-cofilin filaments: The authors report the unexpected finding of axonal regions containing only cofilin-covered actin filaments. To strengthen the claim that the ghost neuron method allows for relating structural findings to the overall organization of neurons, could the authors be more precise about the location of these segments relative to the cell body? Are there other differences in the architecture or nearby structures that might provide insight into the function of these modifications?
- Microtubules/tubulin "stability": The authors describe microtubule segments as "stable" or "dynamic" based on whether the tubulin conformation is "expanded" or "compacted," respectively. While this is structurally sound, in cell biology, microtubule stability is typically defined by its lifetime rather than the intrinsic stability of the lattice. Similarly, the equivalence of expanded to GTP-bound and compacted to GDP-bound tubulin is not absolute within a living cell, where other factors like microtubule-associated proteins (MAPs) and post-translational modifications (PTMs) are at play. The authors should exercise more caution with this terminology, as the manuscript aims to connect structural descriptions with cellular architecture and behavior.
- Microtubule segments: What is the minimum length of an expanded or compacted segment that the authors can resolve? Can this be determined down to a single monomer along a microtubule? This information would be valuable for understanding the "resolution" of the patches depicted in Figures 7 and 8.
- Potential artifact: It is possible that the ghost neuron preparation itself induces changes in microtubule conformation, and that the observed patches of expanded and compacted lattices are a consequence of the procedure. How might the authors test for this possibility? If such controls are not feasible, this should be acknowledged as a potential limitation.

Reviewer #2

(Remarks to the Author)
The Bodakuntla et al present an interesting method to interrogate the structure of cytoskeletal elements from neurons that have been burst by hypotonic shock and mechanical disruption (blotting) before plunge freezing, cryoEM and structure determination of f-actin and microtubules. The authors suggest that the samples retain spatial information corresponding to the locations of neurites on the EM grid and map back structures onto a few areas of MMM montage EM images.

I have raised a number of questions/comments related to specific sentences in the text and figures. I have also raised 4 major comments. Overall, I am not yet convinced that the method gives rise to samples in which the native subcellular environment is preserved and would suggest this manuscript requires significant revision.

Abstract:

“By combining hypotonic treatment with gentle mechanical disruption, the
technique selectively removes the plasma membrane while preserving internal organization”
Not sure what ‘gentle’ means here. Seems subjective. I would prefer ‘partial’.
Internal organization of what? Need to specify.

“We also obtained a near-atomic structure of axonal F-actin
and identified a subpopulation fully decorated with cofilin (cofilactin)”
Saying ‘near-atomic’ is non-specific and a controversial phrase. Please state the resolution. Or could say ‘high resolution’.

Introduction:

L33: Not sure “phenomena” is the correct word. Do you mean ‘macromolecular structures’ or ‘structural basis of complex cellular mechanisms’?

L37: “Cryo-FIB prepares…”. This sentence needs amending. It is currently unclear.

L47: “Although neurites typically…”. This sentence is a little too simplistic. What is the ‘permissible thickness range’? More importantly, what evidence is there that it is the membrane in particular that limits the resolution in cryoET/cryoEM? Do membranes inelastically scatter more than the solvent or proteins within the cytoplasm of neurites?

L67: “Due to their extreme elongation
spanning up to a meter in higher organisms, neurons exhibit compartmentalized, localized

functions”. Not sure what you mean by ‘localised functions’. Do you mean specialised subcellular compartments? Please provide example and citations.
While neurons can be very long and do have specialized subcellular compartments, I don’t think there is any evidence of a teleiological relationship (one arising because of the other). If there is, please provide a reference.

L79: “This process removes the outer membrane barrier while preserving the
internal architecture”

Results:
L99: “…we found that the
gross morphology of the axons remained unaffected across these conditions after initial exposure
to hypotonic media”. Please re-write this. What do you mean by ‘initial’. Please state a range of times. Please also provide a figure citation for this result.

L106: “We further examined the impact of hypotonic stress on organelle motility using light microscopy
as a functional test.” Please re-write. Light microscopy is not a functional test. Rather, ‘organelle motility’ is the functional test and light microscopy provides the assay readout.

L119: “In contrast, both 160 mOsm and 80 mOsm treatment successfully removed the plasma membrane
while preserving the intracellular organization of axons (Fig. 4B and Fig. S2B).”
What do you mean by “intracellular organization of axons”? Need to specify what has been measured?

Presumably upon removal of the membrane, some intracellular macromolecular constituents some cytoplasmic proteins can escape? Figures showing medium magnification (MMM) cryoEM micrographs of the unroofed cells show this with organelles distributed in neighbouring holes of the cryoEM grid. It is important to describe this in the text.

A single MMM figure of a ‘ghost neuron preparation’ (with 150 mOsM prep) containing parallel arrays of microtubules (Fig. 4B) not enclosed by a plasma membrane that are reminiscent of a neurite. It would be good to show a few more examples of this.

Major comment 1:

Given that microtubules are aligned within neurites, is it possible to use prior live imaging fluorescence of the cells (MemGlow fluorescent label used in Figs. 2 &5) grown on EM grids and align this with the MMM images to confirm that the trajectory of microtubules in the MMM image is aligned and follows the trajectory of the neurites? This cryoCLEM-like approach would provide evidence that these cryoEM data represent the arrangement of cytoskeletal elements as they were in the neuron when it was alive. This may be important to rule out the possibility that the preparation (hypotonic shock + blotting) is simply homogenising the cell on the EM grid and destroying the cellular context of what remains.

The authors established a protocol using fluorescent anti-microtubule antibodies to test that the ghost neuron preparation allows access to the intracellular antigens. Pleasingly, the authors used triton-X100 treatment blunge freezing as a positive control (Fig. S3), which showed the fluorescent antibody marking microtubules as long rod-shaped structures aligned with neurites, as expected.

L131: “Under 160 and 80 mOsm, clear microtubule labeling was observed,
confirming membrane disruption, while 240 mOsm-treated samples showed no staining (Fig. 5,
Movie 4)”.

Major comment 2:*

The results section does not accurately describe the fluorescence labelling of microtubules in Fig. 5. For example, Fig. 5C corresponding to the conditions that the authors suggest is optimal for their ‘ghost neuron’ preparation does not show the distribution of microtubules as one would expect (following the route of neurites) as shown in the control figure (Fig. S3). Instead, the distribution of microtubules look like randomly distributed aggregates/clumps. Could this suggest that the ghost neuron preparation does not accurately represent a “cellular map” of microtubules and that these cytoskeletal elements have been moved, damaged/homogenized by the ghost neuron preparation?

L143: “These montages capture spatial information such as the localization of major cellular components including mitochondria and F-actin, as well as the density and arrangement of axonal microtubules”. How can the authors distinguish axonal versus dendritic microtubules?

L145: “From the montages, we found that the microtubule cytoskeleton remains largely preserved in ghost neurons, allowing visualization of its spatial organization and potential interactions with associated components (Fig. S4A and S4B)”. This statement appears to contradict the data in Fig. 5 (see ‘major comment 2’ above).

L152: “Serving as cellular maps, these montages guide targeted data collection by cryo-ET or single-particle analysis (SPA) to acquire high-resolution structural information from regions of interest. Using this approach, we successfully reconstructed a structure of undecorated axonal F-actin at a resolution of 3.24 Å (Fig. 6A and 6B)”. Need to be more specific - is this structure obtained from STA or SPA? How do you know it is “axonal”?

Also, please state if montages were collected for all regions of the grids from which high-resolution micrographs acquired for SPA? If not, how many?

L153: “Notably, we identified a distinct subpopulation of F-actin that was fully decorated on their surface”. How? Classification of particles? This is unclear.

L160: “Interestingly, these cofilin-bound actin filaments were clustered in discrete regions along axons, where most individual filaments were fully decorated with cofilin.” This is interesting, but alas no evidence or figure citation is provided to support this claim.

Major comment 3:

The authors also determine the structure of microtubules by SPA, identifying compact (more labile) and stretched (more stable) forms:
L183: “our data provide the first direct in situ evidence for the presence of these distinct lattice conformations within the native neuronal environment”. This statement that these structures aareew representative of these proteins in their “native neuronal environment” seems overblown and misleading since the samples to not contain a membrane.

The authors then map back into MMM images the location of coordinates giving rise to the stretched versus compact microtubule structure, shown in Fig. 8.
L195: “This back-plotting approach allowed the visualization of microtubule conformational states in situ across axonal compartments”. How do the authors know these are microtubules within axons versus dendrites? I wonder if the authors can deduce the polarity orientation of the microtubules mapped back into the MMM? If they are axonal, I suppose one would find most microtubules are uniformly oriented (‘+’ end pointing away from the cell body).

This MMM mapping analysis is presented in Fig. 8 showing the distribution of stretched versus compact tubulins in only 10 microtubules in a single ghost neurite. The concern in ‘major comment 2’ above that the ghost neuron preparation has moved or damaged these cytoskeletal elements might call into question the validity of mapping back structures into the MMM images. Specifically, how can the authors rule out that the ghost-method preparation of removing the membrane such that the cytoplasm mixes with the media surrounding the cells during blotting would not have altered microtubule stability and contributed to the pattern of stretched/compact regions in each microtubule?

Additionally, a more extensive quantification, including more examples of this mapping to other MMM of ghost neurites in their dataset may be necessary to convince the reader of the interpretation of the data that:
L197: “our back-plotting analysis revealed that stretched lattices, associated with increased microtubule stability, also appeared in mid-sections of axonal microtubules (Fig. 8).”

Discussion:

Major comment 4:

L217: “approach preserves the native subcellular architecture”.
L224: “This method preserves intracellular structures with minimal perturbation and significantly enhances the signal-to-noise ratio, a critical factor for accurate alignment during cryo-EM image processing and 3D reconstruction “
This method preserves intracellular structures with minimal perturbation”
These statements are inaccurate/overblown. At best microtubule subcellular architecture is partially preserved (not withstanding Fig. 5, see ‘major comment 2’), but most other constituents of these subcellular neuronal structures are clearly not preserved.

I am also not convinced that the increased contrast observed in the cryoEM data is simply consequence of removing the membrane. Is it not also likely that removing the membrane could also enable thinner specimens to be prepared? This would be the major contribution to the increased contrast (SNR) evident in the data. Surely removing the membrane and blotting, would permit intracellular macromoleucular constituents to diffuse outward/flatten out as blotting reduces the thickness of the thin film before plunge freezing?

L268: “Additionally, while the method allows visualization of retained vesicles and membrane-associated components”. This statement could be misunderstood. Since the plasma membrane is removed during the ghost neuron preparation, these ‘membrane-associated components’ can’t possibly be visualised can they?

Methods:

L473: “In experiments mimicking cryo-blotting, grids with thalamus explant tissue were initially imaged with MemGlow and DIC for 5 minutes. Subsequently, the grids were treated with hypotonic media and manually back-blotted with the same filter paper used for vitrification for 3-4 seconds before being placed back into the imaging chamber”
When mimicking cryo-blotting (related to data in Fig. 5), why were the cells back-blotted with a Leica and not front-and-back blotted with a vitrobot as described for cryoEM grid preparation (as described L508)?

L587, Fig. 5: Some panel insets lack scale bars and the scale bar in other panels have not been defined in the legend).

Reviewer #3

(Remarks to the Author)
Bodakuntla et al. present the ghost neuron technique, a preparation method for cryo-electron microscopy imaging of neurons based on removing the plasma membrane. Hypotonic treatment followed by mechanical disruption of the cell improves image contrast and signal-to-noise ratio. Using this approach, the authors obtained structures of axonal F-actin and cofilin-decorated filaments as well as two distinct conformations of microtubule lattices corresponding to stable and dynamic states. Mapping these structural variants onto neuronal geometry revealed coexistence of stable and labile microtubule segments within single axons.

The authors claim that their method removes the plasma membrane while preserving internal organization. In relatively thick specimens, multiple inelastic scattering events dominate image formation, resulting in high background noise and reduced contrast. Because the thin plasma membrane contributes negligibly to total scattering, its removal would not produce any visible difference in contrast. Therefore, the observed increase in contrast must reflect extraction and rearrangement of intracellular material, leading to reduced thickness and macromolecular crowding. Figure 4B shows spread out microtubules in ghost neuron while in the intact neurite shown in Figure 4A, the microtubules seem much more densely packed.

The application of ghost neuron is mostly restricted to the study of cytoskeleton. And even if it is closer to native state than in vitro reconstitution, the ghost neuron system is far from an intact cell. Although it preserves the intracellular organization better than in vitro reconstitutions, it still represents a system that is far from an intact, living cell.

More specific points are listed below.
l. 82. The term in situ is inappropriate for the reasons explained above

l. 100. The authors mention that the morphology of axons remained unaffected after initial exposure to hypotonic media (Figure 2). First, we cannot distinguish dendrites from axons in figure 2. Second, even if the morphology of the plasma membrane at the light microscopy level seems intact, the figure does not prove that the morphology of the plasma membrane, and even more so of the interior of the cell is preserved at a higher resolution than than the images shown in Figure 2.

l. 107. The authors observed that reducing osmolarity up to 50% did not significantly influence mitochondrial motility. Nonetheless, the physiology of the neurons is likely strongly affected by a drop of 50% in osmolarity, which is used later to prepare ghost neurons. This might result in very rapid structural changes at the molecular level.

l. 115. What does gentle blotting mean?

Mitochondria in axons and dendrites are quite elongated. Yet, in the ghost neurons, mitochondria look mostly round. This indicates that they have undergone significant morphological changes.

The authors claim resolutions ranging from 3.26 to 5.32 Å for their single particle analysis reconstructions. The cryo-EM maps were not provided and a visual inpection cannot confirm these values. The PDB EM validation reports do not at all support the resolutions claimed by the authors. Indeed, instead of 3.26 Å, the report gives a resolution of 7.26 Å. For the map claimed at 3.66 Å, the report finds 4.45 Å. For the one claimed at 3.96 Å, the report says 6.18 Å. And for the map claimed at 5.32 Å, a resolution of 9.04 Å is calculated by the validation report. It seems that the masks used by the authors for resolution estimate are too tight and lead to significant resolution overestimate.

l. 197. The distribution of the two types of microtubule lattice is reported. Yet, it is not clear how robust the detection of the type is. This should be quantified. The distribution of likelihood of each particular microtubule segment to be of one or the other type should be shown.
More over, the claim that the found distribution reflects the situation in situ should be backed by distribution obtained in cryo-ET data of intact neurons.

l.220. Neurites, which are the target cell regions of the ghost neuron technique, are actually accessible by cryo-ET without the need of cryo-FIB milling. Thus the advantage of the technique is not obvious.

It is not indicated how long the cells are exposed to low osmolarity before plunge freezing.

Version 2:

Reviewer comments:

Reviewer #1

(Remarks to the Author)
In their revised manuscript, Bodakuntla et al. have addressed some of the key concerns raised in the reviews. In particular, my points about representation of previous works, better delineation fo the specific advantages and limitations of their ghost neurons procedure, and discussion of the procedure effect on the conformational state of microtubules have been thoroughly addressed. I have a couple of remaining points, mostly on the microtubule conformation results and interpretations, that can be addressed as text revisions before recommending acceptance for publication.

1. It would be better to unify the nomenclature of the tubulin conformation and use "expanded" throughout (as used in most of the literature), rather than "stretched".

2. The link between expanded/GTP/stable and compacted/GDP/labile (or dynamic) should still be toned down as it reflects a vision of microtubule structure rooted in classic articles on plus ends polymerization, rather than the more nuanced view of the states within the lattice away from plus ends, which are what the authors focus on.

3. The revised explanation and data regarding the "resolution" of the conformation assignment down to a single monomer is convincing. Still for a non-specialist it is a bit surprising that the difference in length between expanded and compacted conformations (1.9 Å) is lower than the resolution of the EM data – it might be useful to explain how that's possible.

4. Regarding the conformation classification, it's not entirely clear how the authors validate that there are only two states (expanded or compacted with no intermediates). What about the unlabeled monomers in Fig. 7, 8 and S7? Do they represent segments where assignment fails, or states that are not one or the other? Overall in the dataset, what is the % of imaged microtubule length that is 1) assigned to expanded conformation 2) assigned to compacted conformation 3) not assigned to one or the other?

5. Regarding the validation of the conformation classification, it would be useful to show a plus end from the existing data, as plus ends are known to have an expanded conformation cap followed by a compacted lattice.

6. A recent article from the Roll-Mecak lab (Zehr et al. Nat Struct Mol Biol 2026) has examined the tubulin compaction state along the axons of neurons, proposing that all monomers are found in the expanded state. It would be good if the authors could mention this and explain why they obtain a different result (mix of compacted and expanded states).

Reviewer #2

(Remarks to the Author)
This manuscript is much improved. The authors have answered my comments with major revisions of the text and figures, and included new cryoCLEM data. Together, these address the major concerns I had. I congratulate the authors on an excellent study and recommend this manuscript for publication with minor changes (not requiring further peer-review).

Minor changes:

Title: "Ghost neuron unlocks in situ high-resolution structural mapping of intracellular architecture in neurons". Since the ghost neuron preparation is, as the authors acknowledge, not exactly 'in situ' and this terminology has been taken out of the maintext in the revised manuscript, it might be necessary to change the title also. The current title also has the 'neuron' noun twice, which seems a bit clunky. Could go with 'Ghost neuron preparation unlocks high-resolution structures with cellular context'.

L160: “suggesting that neurons can withstand short-term hypotonic”. This needs to be slightly more cautiously written because though neuronal swellings may not arise and axonal transport was unperturbed, electrophysiological functions are almost certainly impaired (even within a few seconds).

L328: “by conventional cryo-ET or extraction-based methods”. Using the term ‘conventional cryo-ET’ here and elsewhere in the manuscript is ambiguous and annoy the cryo-ET community. I strongly recommend being more specific eg. rephrase to “by cryoET of intact cells”.

There may be the odd minor grammatical error – I haven’t combed the text this closely – please check.

Reviewer #3

(Remarks to the Author)
The authors have addressed satisfactorily all my concerns. The manuscript has been significantly improved and should be published. I noticed two minor points: the newly added author, S. Glauser, is not listed in the author contribution section; in table S1, two maps have the same code (EMD-76718).

Open Access This Peer Review File is licensed under a Creative Commons Attribution 4.0 International License, which permits use, sharing, adaptation, distribution and reproduction in any medium or format, as long as you give appropriate credit to the original author(s) and the source, provide a link to the Creative Commons license, and indicate if changes were made.
In cases where reviewers are anonymous, credit should be given to 'Anonymous Referee' and the source.
The images or other third party material in this Peer Review File are included in the article's Creative Commons license, unless indicated otherwise in a credit line to the material. If material is not included in the article's Creative Commons license and your intended use is not permitted by statutory regulation or exceeds the permitted use, you will need to obtain permission directly from the copyright holder.
To view a copy of this license, visit https://creativecommons.org/licenses/by/4.0/

Reviewer #1

This manuscript by Bodadkuntla et al. introduces a procedure for creating "ghost" neurons by employing a low osmolarity treatment combined with mechanical shearing via paper blotting. This method effectively removes the plasma membrane along neuronal processes, primarily axons, which enhances the contrast for subsequent cryo-electron tomography (cryo-ET) examination. The authors apply this technique to study actin and microtubules within axons, utilizing single-particle averaging to reveal the presence of cofilin-covered actin filaments and distinct segments of expanded and compacted lattices along individual axonal microtubules. While the manuscript is of high quality and successfully demonstrates the utility of this new procedure, several concerns must be addressed before it can be recommended for publication.

Incomplete Context and Discussion of Results

A central argument of the manuscript is the novelty of the procedure and the applications it enables. However, it omits key studies that are essential for providing a comprehensive view of the current state of the art. These should be cited in the Introduction and discussed in subsequent sections.

- The Introduction suggests that cryo-EM along neuronal processes lacks sufficient contrast, thereby justifying the development of the ghost neuron procedure. However, a significant body of existing work has successfully performed cryo-EM along neurites, including cryo-ET, single-particle averaging, and structural assessment of cytoskeletal assemblies (Petrovic et al., Curr Op Neurobiol 2025). This includes research from the Baumeister/Lučić labs, the Carter lab (Foster et al., JCB 2022), the Kukulski lab (Hoffman et al., eLife 2021), the Moores lab (Atherton et al., Acta Crystallograph Sect D 2018), Zamponi et al. (Dev Cell 2022), and even the authors' own lab (Nedozralova et al., JCB 2022, which is only cited in the Methods section). These prior studies should be properly acknowledged, and their limitations must be clearly explained to justify the need for the new method.

We agree and thank the reviewer for pointing out this important aspect. We have added the pioneering efforts of the field and also explain the unique aspect of our method compared to conventional cryo-ET. The corresponding statements are found in the Introduction as follows:

"Despite these recent progress, obtaining high-resolution information from certain cellular regions remains challenging, specifically from thin, elongated neurites. Although neurites fall within the geometric thickness limits of cryo-EM and cryo-ET, their dense cytoplasm and the abundance of membrane-bound organelles substantially reduce image contrast, limiting alignment accuracy, and ultimately constraining achievable resolution. Several studies have successfully imaged neuronal processes by cryo-EM and cryo-ET (Hoffmann et al., 2021; Atherton et al., 2018; 2022; Foster et al., 2022; Nedozralova et al., 2022; Petrovic et al., 2025; Zamponi et al., 2022), showing that neurites are somewhat accessible to cryogenic imaging, while also revealing persistent challenges obtaining information at a molecular resolution due to poor contrast and cytoplasmic densities."

- In the discussion of plasma membrane removal techniques, the authors mention unroofing (citing Vassilopoulos et al., Nat Comm 2019) and the preparation of red blood cell ghosts. The manuscript fails to include surfactant-induced membrane removal prior to fixation, a method used extensively by the Svitkina lab to visualize cytoskeletal organization, including in neuronal processes (Korobova et al., MBC 2010; Jones et al., JCB 2014; Efimova et al., J Neuro 2017).

We thank the reviewer for the helpful suggestion to incorporate the pioneering work by the Svitkina laboratory (Korobova et al., 2010; Jones et al., 2014; Efimova et al., 2017) as well. We did not include these articles originally because the approach does not yield high-resolution information due to the platinum-replica method. However, we also agree that these studies are highly informative and have provided important insights into cytoskeletal organization in neurons. We have added a paragraph

to the Introduction to acknowledge these earlier studies while clarifying the distinct capabilities and scope of the ghost neuron preparation.
“Related surfactant-based extraction methods have provided informative access to cytoskeletal organization including neuronal processes (Efimova et al., 2017; Jones et al., 2014; Korobova and Svitkina, 2010), however, these methods are largely limited by the usage of chemical fixation and metal coating and therefore incompatible with high-resolution cryo-EM in a frozen-hydrated state.”

- In the Results section, the authors state, “While these lattice states have been characterized in vitro using cryo-EM with defined nucleotide conditions, our data provide the first direct in situ evidence for the presence of these distinct lattice conformations within the native neuronal environment” (lines 182-183). This overlooks the work of de Jager et al. (JCB 2024), which identified expanded and compacted lattices in cells using cryo-ET (even if not in neurons). This study is highly relevant to the assessment of tubulin lattice states - one of the two main applications of the ghost neuron procedure - and should be cited and discussed.
We have added this observation to reinforce the idea that cells can contain microtubules with stretched and compact lattice conformations. It now reads: “In addition, the expanded lattice states have been detected in intact mammalian U2OS cells by cryo-ET (de Jager et al.,), supporting the lattice heterogeneity within a cell.”

- The Discussion lists the advantages of the ghost neuron procedure (lines 247-258), but it remains unclear how these benefits are unique compared to unroofing or detergent-induced membrane removal in similar culture models.
We agree that various membrane-removal strategies have long been used. The distinction of the Ghost Neuron approach does not lie in membrane removal, but in the timing, preservation state, and compatibility with high-resolution cryo-EM. In contrast to classical unroofing or detergent-based extraction, membrane rupture in the Ghost Neuron procedure occurs directly on EM grids and is followed immediately by vitrification. This minimizes the time window for cytoskeletal rearrangement, depolymerization, or dehydration. Traditional unroofing approaches require chemical fixation or metal coating prior to imaging and are therefore not compatible with frozen-hydrated high-resolution cryo-EM or single-particle analysis of released intracellular complexes theirefor not compatible with high-resolution analyses. Furthermore, the Ghost Neuron method preserves sufficient cytoskeletal continuity to allow spatial back-mapping of high-resolution structures onto neurite trajectories, while simultaneously generating a dispersed particle fraction suitable for near-atomic resolution SPA. This combination of rapid mechanical membrane rupture, vitrification in a native hydrated state, and molecular-resolution structural readout within neuronal processes distinguishes the Ghost Neuron approach from existing membrane-removal techniques. We have clarified these distinctions in the revised Discussion.

Manuscript Structure
- The purpose of the "Ghost neuron specification" section (lines 75-89), positioned between the Introduction and Results, is unclear as it appears to summarize the Results. Furthermore, the final paragraph of the Introduction (lines 62-73) repeats arguments from previous paragraphs. It is suggested that both be reworked into a single, concise final paragraph for the Introduction.
We have removed the section and integrated the essential details into the final paragraph of the Introduction and the opening of the Results section.

- The "Material availability" section (lines 289-291) does not state how the materials can be accessed.

We have added the statement: Information and requests for reagents may be addressed to the Lead Contact, Naoko Mizuno (Naoko.mizuno@nih.gov). The cryo-EM reconstructions of stable (stretched) and labile (compact) microtubules were deposited under EMD-76717 and EMD-76716, respectively. The corresponding microtubules coordinates were deposited under PDB 12ru (stable/stretched) and PDB 12rt (labile/compact). The F-actin reconstruction was deposited under EMD-76718 with coordinates PDB 12rv (β-actin). The colfilactin (cofilin-decorated F-actin) reconstruction was deposited under EMD-71056.

Limitations of the Study
The primary limitation of this procedure, which is only partially addressed, is that it scrambles the organization of submembrane scaffolds. This is particularly significant in neurons, where a periodic skeleton of actin rings connected by spectrin tetramers - known as the membrane-associated skeleton (MPS) - is a fundamental structural component (Xu et al., Science 2013). The MPS has been observed in all neurons and species studied, primarily through optical super-resolution techniques and platinum replica EM after unroofing (reviewed in Leterrier, Curr Op Neurobiol 2021). Notably, no existing cryo-EM/cryo-ET study has successfully visualized the MPS. While a new procedure for improved visualization of axonal architecture is welcome, this method does not address this main shortcoming of existing techniques. The disordering of the submembrane scaffold is acknowledged in the Results when discussing actin organization (lines 148-150) , although the meaning of "approximate localization was maintained" is unclear. To clarify the consequences of this, a brief description of the MPS and key references should be included. The consequence of this disruption, namely the inability to visualize the MPS, should be explicitly stated as a key limitation of the technique.
We agree that disruption of submembrane scaffolds is a key limitation of the Ghost Neuron preparation. In the revised manuscript, we have expanded the Limitations section to explicitly discuss the membrane-associated periodic skeleton (MPS) and clarify that, because the plasma membrane is ruptured prior to vitrification, submembranous structures mechanically coupled to the membrane—including the periodic actin–spectrin architecture of the MPS—are not preserved.

Experimental Questions
- Actin-cofilin filaments: The authors report the unexpected finding of axonal regions containing only cofilin-covered actin filaments. To strengthen the claim that the ghost neuron method allows for relating structural findings to the overall organization of neurons, could the authors be more precise about the location of these segments relative to the cell body? Are there other differences in the architecture or nearby structures that might provide insight into the function of these modifications?
Cofilin-mediated actin remodeling has been reported in growth cones and regions of active cytoskeletal turnover. In our dataset, the cofilin-decorated actin filaments were identified in axon shafts more than 250 µm away from cell bodies as we focused on axon architexture. For these micrographs however, we did not perform systematic spatial mapping relative to the cell body or growth cone. Based on the available contextual information, these filaments were not clearly associated with obvious growth cone morphology, branch points, or other distinctive architectural landmarks. Rather, they appeared within otherwise typical axonal segments. We have clarified in the revised manuscript that our observation of cofilin-decorated filaments as a structural identification, without making specific claims btout their positional enrichment along neurites. Determining whether such assemblies correlate with growth cones or other actin-remodeling zones will require future studies combining spatial mapping and higher-throughput analysis with a larger set of data.

- Microtubules/tubulin "stability": The authors describe microtubule segments as "stable" or "dynamic" based on whether the tubulin conformation is "expanded" or "compacted," respectively. While this is structurally sound, in cell biology, microtubule stability is typically defined by its lifetime rather than the intrinsic stability of the lattice. Similarly, the equivalence of expanded to GTP-bound and compacted to GDP-bound tubulin is not absolute within a living cell, where other factors like microtubule-associated proteins (MAPs) and post-translational modifications (PTMs) are at play. The authors should exercise more caution with this terminology, as the manuscript aims to connect structural descriptions with cellular architecture and behavior.

We agree that, in cell biology, microtubule stability is often defined operationally by filament lifetime and turnover rather than by intrinsic lattice conformation alone. Our original wording may have unintentionally conflated these distinct concepts. In the revised manuscript, we have adjusted the terminology to distinguish between structural lattice state and functional microtubule lifetime, and add the explanation of the relationship between the lattice state and functional state. When necessary, we now use the word 'labile' microtubules rather than dynamic microtubules. In the revision, we also explicitly clarified that, in cells lattice state is influenced by multiple factors—including MAP binding, post-translational modifications, and nucleotide states. We believe this clarification makes the connection between structural observations and cellular context clearer without overstating functional implications.

- Microtubule segments: What is the minimum length of an expanded or compacted segment that the authors can resolve? Can this be determined down to a single monomer along a microtubule? This information would be valuable for understanding the "resolution" of the patches depicted in Figures 7 and 8.

That question is an important technical insight to address the axial resolving power of lattice classification. In our analysis, microtubules were segmented at ~82 Å intervals (corresponding to one tubulin heterodimer repeat), and C1 classification was performed using a focused mask spanning ~120 Å length, spanning to cover two protofilaments. Therefore, each classified segment reports the conformation of a local lattice patch approximately at the single-heterodimer scale, as with some smoothing imposed by the mask width. Accordingly, the minimal resolvable length of an expanded or compacted segment is approximately one heterodimer repeat (~8 nm), though transitions between states may appear spatially broadened due to the classification window. We have added this point in the Results and Methods section to better define the axial resolvable unit of the mapped patches.

- Potential artifact: It is possible that the ghost neuron preparation itself induces changes in microtubule conformation, and that the observed patches of expanded and compacted lattices are a consequence of the procedure. How might the authors test for this possibility? If such controls are not feasible, this should be acknowledged as a potential limitation.

We agree that preparation-induced artifacts must be considered. In the revised manuscript, we have expanded our discussion of this possibility. The measured axial repeat distances (~81 or 83 Å) closely match the canonical compact and expanded lattice parameters previously reported *in vitro*, indicating that we are reporting established lattice states rather than distorted intermediates. In addition, hypotonic exposure and mechanical rupture occur immediately prior to blotting and vitrification, providing limited opportunity for coordinated longitudinal lattice transitions along pre-assembled microtubules, which would be required for the observable lattice compactation.

To further examine whether lattice heterogeneity could arise during the Ghost Neuron preparation, we also inspected power spectra from cryo-ET data of intact neurites in which the plasma membrane remains present (similar way to (de Jager et al., PMID: 39387699)). The strong ~40–42 Å layer line (the

second harmonic of the tubulin dimer repeat) appeared broadened or partially split, consistent with the presence of nearby lattice periodicities within individual microtubules. Representative examples are shown below. However, because the signal-to-noise ratio of tomographic data limits robust classification of lattice states, we consider this line of observation rather illustrative than quantitative at this point. A rigorous quantitative evaluation of lattice heterogeneity directly in intact neurons would require substantially larger tomographic datasets and systematic analyses that extend beyond the scope of the present study.
Together, the agreement with established lattice parameters, the limited timescale of the preparation, and the presence of heterogeneous lattice signatures in intact neurites suggest that the compact and expanded segments observed in the Ghost Neuron dataset reflect pre-existing structural states rather than preparation-induced remodeling. We have clarified this point in the revised Discussion.

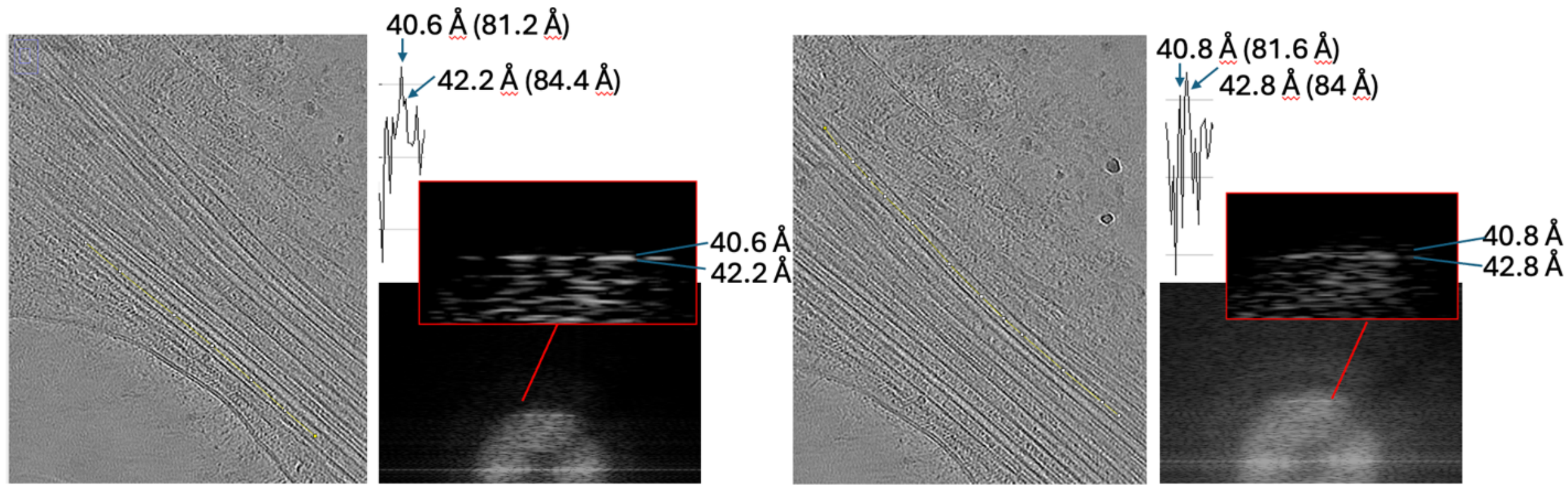

Example 1 Example 2

Reviewer #2

The Bodakuntla et al present an interesting method to interrogate the structure of cytoskeletal elements from neurons that have been burst by hypotonic shock and mechanical disruption (blotting) before plunge freezing, cryoEM and structure determination of f-actin and microtubules. The authors suggest that the samples retain spatial information corresponding to the locations of neurites on the EM grid and map back structures onto a few areas of MMM montage EM images. I have raised a number of questions/comments related to specific sentences in the text and figures. I have also raised 4 major comments. Overall, I am not yet convinced that the method gives rise to samples in which the native subcellular environment is preserved and would suggest this manuscript requires significant revision.

Abstract:
"By combining hypotonic treatment with gentle mechanical disruption, the technique selectively removes the plasma membrane while preserving internal organization"
Not sure what 'gentle' means here. Seems subjective. I would prefer 'partial'.
Internal organization of what? Need to specify.
We agree that the term "gentle" is subjective and may be unclear. We have revised the sentence in the Abstract to remove this wording and the revised sentence now reads:
"By combining hypotonic treatment with mechanically induced membrane rupture immediately before plunge freezing, the method selectively removes the plasma membrane while preserving neurite geometry."

"We also obtained a near-atomic structure of axonal F-actin

and identified a subpopulation fully decorated with cofilin (cofilactin)”
Saying ‘near-atomic’ is non-specific and a controversial phrase. Please state the resolution. Or could say ‘high resolution’.
We agree with the reviewer that usage of ‘near-atomic’ is not objective. We removed all ‘near-atomic’ phrases from the manuscript, and replaced either with the obtained resolution or ‘high resolution’.

Introduction:
L33: Not sure “phenomena” is the correct word. Do you mean ‘macromolecular structures’ or ‘structural basis of complex cellular mechanisms’?
Our original use of the word “phenomena” was intended to convey that *in situ* cryo-EM/cryo-ET has revealed not only individual macromolecular structures, but also unexpected cellular behaviors, spatial organizations, and higher-order assemblies that emerge from molecular interactions in their native context. To avoid ambiguity, we have revised the sentence to: “This powerful imaging capability has led to unexpected discoveries of biological macromolecular assemblies underlying complex cellular functions, revealing molecular organization and emergent cellular behaviors directly within their native environment.”

L37: “Cryo-FIB prepares...”. This sentence needs amending. It is currently unclear.
We corrected the error and now it reads ‘Cryo-FIB produces thin lamellae suitable for cryo-EM, allowing visualization of structures otherwise inaccessible in intact cells.’

L47: “Although neurites typically...”. This sentence is a little too simplistic. What is the ‘permissible thickness range’? More importantly, what evidence is there that it is the membrane in particular that limits the resolution in cryoET/cryoEM? Do membranes inelastically scatter more than the solvent or proteins within the cytoplasm of neurites?
The reviewer is correct that the plasma membrane itself is not the primary contributor to contrast loss in neurites. Rather, the combination of cytoplasmic density through the enclosure of the plasma membrane, organelles, and membrane-bounded compartments increases overall mass thickness and reduces image contrast. We have revised the text to reflect this more accurate explanation.

L67: “Due to their extreme elongation spanning up to a meter in higher organisms, neurons exhibit compartmentalized, localized functions”. Not sure what you mean by ‘localised functions’. Do you mean specialised subcellular compartments? Please provide example and citations. While neurons can be very long and do have specialized subcellular compartments, I don’t think there is any evidence of a teleiological relationship (one arising because of the other). If there is, please provide a reference.
We thank the reviewer for pointing out the ambiguity and the unintended implication of a teleological relationship between axonal length and functional compartmentalization. Our intent was not to suggest that elongation causes localized functions, but rather to highlight that axons contain specialized subcellular microdomains—such as branch initiation sites, presynaptic boutons, localized translation zones, and signaling hotspots—that support spatially restricted regulation of neuronal processes. We have revised the sentence accordingly to avoid any causal inference and to incorporate explicit examples with references (now at the beginning of Discussion section: Neurons exhibit extreme polarization, with specialized subcellular microdomains distributed along elongated neurites (Kapitein and Hoogenraad, 2015), including sites of growth cone (Lowery and Van Vactor, 2009), branch initiation (Kalil and Dent, 2014; Nedozralova et al., 2022), synaptic terminals (Murthy and De Camilli, 2003), localized translation hotspots (Holt and Schuman, 2013) , and transport and signaling hubs (Terenzio et al., 2017).).

L79: "This process removes the outer membrane barrier while preserving the internal architecture"

We have revised this sentence to "This combined procedure selectively ruptures the plasma membrane while leaving the underlying cytoskeletal architecture and overall neurite geometry largely intact." We have also discussed that a fraction of macromolecular material may become dispersed during preparation, which can be advantageous for single-particle analysis (SPA), albeit without preservation of neurite trajectory context. In addition, the paragraph containing the original statement has been reorganized for better clarity.

Results:

L99: "...we found that the gross morphology of the axons remained unaffected across these conditions after initial exposure to hypotonic media". Please re-write this. What do you mean by 'initial'. Please state a range of times. Please also provide a figure citation for this result.

By "initial exposure" we meant the early period during which axons were monitored immediately after switching to hypotonic media. We now specify the time window explicitly: "the gross morphology of axons remained unaffected during the first 10 min of exposure to hypotonic media down to 160 mM Osm (Fig. 2A and 2B)". "the gross morphology of the axons remained unaffected across these conditions during the first 10 min of exposure to hypotonic media down to 160 mOsm (Fig. 2A and 2B). However, incubation in 80 mOsm hypotonic solution caused small swellings to develop on axon membranes (Fig. 2C, middle panel), indicative of membrane instability."

L106: "We further examined the impact of hypotonic stress on organelle motility using light microscopy as a functional test." Please re-write. Light microscopy is not a functional test. Rather, 'organelle motility' is the functional test and light microscopy provides the assay readout.

We have revised the sentence to: "We further examined the impact of hypotonic stress on intracellular dynamics by measuring mitochondrial motility within axons using light microscopy."

L119: "In contrast, both 160 mOsm and 80 mOsm treatment successfully removed the plasma membrane while preserving the intracellular organization of axons (Fig. 4B and Fig. S2B)." What do you mean by "intracellular organization of axons"? Need to specify what has been measured?

We have reorganized the manuscript, and now the portion that contains 'intracellular organization of axons' is removed.

Presumably upon removal of the membrane, some intracellular macromolecular constituents some cytoplasmic proteins can escape? Figures showing medium magnification (MMM) cryoEM micrographs of the unroofed cells show this with organelles distributed in neighbouring holes of the cryoEM grid. It is important to describe this in the text.

The reviewer is correct that a fraction of cytoplasmic material can become dispersed during membrane rupture and blotting. In cryo-EM montages, we frequently observe small vesicles, membrane fragments, and soluble macromolecular material redistributed into neighboring grid holes. In contrast, the majority of larger organelles and the microtubule cytoskeleton remain aligned with the original neurite trajectory. We have now explicitly described this redistribution in the Results section: "As expected for a membrane-removal procedure, some cytoplasmic material became redistributed during blotting, scattering small vesicles, membrane fragments, and soluble macromolecular components into adjacent grid holes (Fig. S3)."

Furthermore, we prepared an example figure with tracing of cellular materials on a montage image (Fig. S3). We also added a paragraph in the discussion explaining that dispersed material can be

used as a particle-rich fraction suitable for high-resolution SPA cryo-EM analysis as a complementary approach while it no longer retains the spatial context.

A single MMM figure of a 'ghost neuron preparation' (with 150 mOsM prep) containing parallel arrays of microtubules (Fig. 4B) not enclosed by a plasma membrane that are reminiscent of a neurite. It would be good to show a few more examples of this.

In the revised manuscript, we have included multiple representative medium-magnification cryo-EM examples of ghost neurons lacking plasma membranes. Specifically, in addition to Fig. 4B, we show Fig. 8, Fig. S2 (comparable examples with back-plotting analysis), Fig. S4A (a high-magnification montage with extensive tracing of microtubules, vesicles, membrane fragments, and mitochondria), Fig. S4B (before-and-after correlative example of neurites, including tracing of microtubule bundles and fluorescent image of neurites), and Fig. S7 (another example of ghosted neurite). Together, these additions show that the observed microtubule organization is reproducible and not limited to a single example.

****Major comment 1:****

Given that microtubules are aligned within neurites, is it possible to use prior live imaging fluorescence of the cells (MemGlow fluorescent label used in Figs. 2 &5) grown on EM grids and align this with the MMM images to confirm that the trajectory of microtubules in the MMM image is aligned and follows the trajectory of the neurites? This cryoCLEM-like approach would provide evidence that these cryoEM data represent the arrangement of cytoskeletal elements as they were in the neuron when it was alive. This may be important to rule out the possibility that the preparation (hypotonic shock + blotting) is simply homogenising the cell on the EM grid and destroying the cellular context of what remains.

We thank the reviewer for the suggestion. Correlating live-cell trajectories on EM grids with subsequent cryo-EM montages is an effective way to test whether the *Ghost Neuron* preparation preserves neurite geometry rather than homogenizing material on the grid. In the revision, we implemented a cryoCLEM-like correlative workflow on the same EM grids: we recorded fluorescence images of neurites prior to vitrification (we used SPY-tubulin to directly label the cytoskeleton trajectory within neuron) and then acquired cryo-EM montages from the corresponding grid regions after ghost-neuron preparation. Side-by-side comparison shows that the membrane-free cytoskeletal bundles imaged by cryo-EM follow the same neurite trajectories observed in the living specimen before preparation (Fig. S4B). We can also visualize that microtubule bundles continue smoothly across the transition at the membrane rupturing point (Fig. S4A). Together, these correlative and segmentation analyses provide evidence that the preparation does not homogenize neurites on the grid but instead preserves the overall axonal scaffold while removing the plasma membrane. Shown in Fig. S4A, we performed a thorough segmentation of cytosolic components in neurites that had undergone ghost preparation. This analysis shows that cytoskeletal elements and larger organelles (e.g., mitochondria) remain aligned along the original neurite trajectory. Local redistribution events can happen to other components and the usage of them are separately discussed in the revised manuscript; these do not alter the overall neurite geometry or bundled microtubule organization.

The authors established a protocol using fluorescent anti-microtubule antibodies to test that the ghost neuron preparation allows access to the intracellular antigens. Pleasingly, the authors used triton-X100 treatment blunge freezing as a positive control (Fig. S3), which showed the fluorescent antibody marking microtubules as long rod-shaped structures aligned with neurites, as expected. L131: "Under 160 and 80 mOsm, clear microtubule labeling was observed,

confirming membrane disruption, while 240 mOsm-treated samples showed no staining (Fig. 5, Movie 4)".
We agree that the original wording did not fully reflect the appearance of the fluorescent signal. Under 160 mOsm conditions, microtubule labeling is observed following membrane rupture; however, the signal appears less continuous than in the Triton-X100 control. This difference is due to the imaging conditions on EM grids (Please refere below with explanation and the combined responses).

****Major comment 2:*****
The results section does not accurately describe the fluorescence labelling of microtubules in Fig. 5. For example, Fig. 5C corresponding to the conditions that the authors suggest is optimal for their 'ghost neuron' preparation does not show the distribution of microtubules as one would expect (following the route of neurites) as shown in the control figure (Fig. S3). Instead, the distribution of microtubules look like randomly distributed aggregates/clumps. Could this suggest that the ghost neuron preparation does not accurately represent a "cellular map" of microtubules and that these cytoskeletal elements have been moved, damaged/homogenized by the ghost neuron preparation?
To directly address the concern of the reviewer whether the ghost-neuron preparation preserves the spatial organization of axonal microtubules, we performed correlative analyses comparing neurite trajectories prior to 'ghosting' with the distribution of microtubules observed after ghost preparation and vitrification (please also refer ***Major comment 1***). Specifically, we traced neurites by fluorescence microscopy before 'ghosting' and compared these trajectories with high-magnification cryo-EM montages acquired from the same grid regions after ghost preparation (Fig. S4B). Microtubule bundles remained aligned with the original neurite paths, indicating that the cytoskeletal scaffold is largely preserved. Although occasional local redistribution of microtubules was observed (example shown in Fig. S4A), the overall trajectory of axonal microtubules remained consistent rather than homogenized or randomized.
We agree that the appearance of Fig. 5C could be misinterpreted as cytoskeletal disruption rather than membrane permeabilization. The original Cy3-based labeling was performed on EM grids, where uneven and multi-planar surfaces reduce optical sectioning quality and increase out-of-focus signal compared to imaging on flat glass substrates (as in the Triton-X control). We also performed the control Triton-X treatment on EM grids, to demonstrate similar kind of outcome (see below). It appears that the used antibody may also not be specific enough to show clear signals.

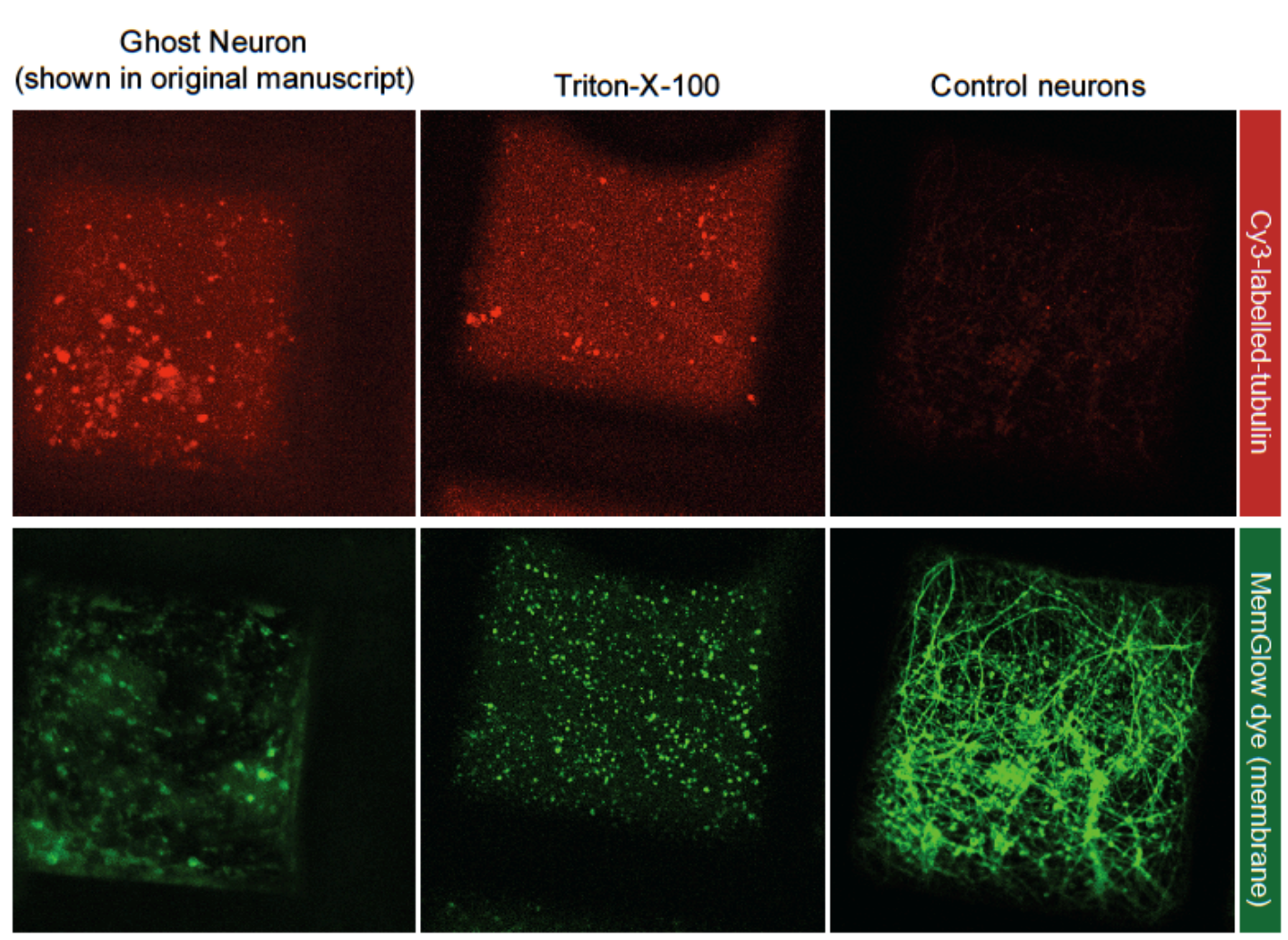

To avoid any misinterpretation of the Cy3-based staining, we changed the labelling method to fluorescent Epothilone B, a tubulin dye that does not permeate intact membranes but clearly labels microtubules upon membrane disruption. Under these conditions, labeling was observed only after blotting-induced membrane rupture. We replaced the Cy3-antibody data with the fluorescent EpoB assay to demonstrate membrane permeabilization more clearly. A reference to the synthesis of the fluorescent EpoB and the contributor have been added to the manuscript accordingly.

L143: "These montages capture spatial information such as the localization of major cellular components including mitochondria and F-actin, as well as the density and arrangement of axonal microtubules". How can the authors distinguish axonal versus dendritic microtubules?

Our dataset was acquired from thalamic explant cultures, in which the long, thin neurites extending onto EM grids correspond to axons (≥200 μm away from cell bodies). We have added a clarifying sentence to the manuscript together with references: 'We used thalamus neural explants, in which long neurites extending from thalamic tissue are axons at this developmental stage, allowing unambiguous assignment of the observed neurites as axonal origins (PMID: 41224993, 35262630, 10328943).'

L145: "From the montages, we found that the microtubule cytoskeleton remains largely preserved in ghost neurons, allowing visualization of its spatial organization and potential interactions with associated components (Fig. S4A and S4B)". This statement appears to contradict the data in Fig. 5 (see 'major comment 2' above).

The conclusion that the microtubule cytoskeleton remains largely preserved is based on direct cryo-EM montage data, in which microtubule bundles can be traced continuously along neurite trajectories and across membrane-intact and membrane-free regions. To strengthen this point, we traced the materials that were left after rupturing (ghosted), and compared to the original neurite pattern without the ghost treatment, which we have now added as Fig. S3. As clarified above, old Fig. 5 reflects fluorescence imaging on uneven EM grids and is intended to demonstrate membrane disruption rather than cytoskeletal organization and we replaced the labeling dye to fluorescent EpoB.

L152: "Serving as cellular maps, these montages guide targeted data collection by cryo-ET or single-particle analysis (SPA) to acquire high-resolution structural information from regions of interest. Using this approach, we successfully reconstructed a structure of undecorated axonal F-actin at a resolution of 3.24 Å (Fig. 6A and 6B)". Need to be more specific - is this structure obtained from STA or SPA? How do you know it is "axonal"?

The F-actin structure was obtained by single-particle analysis (SPA), not subtomogram averaging. We have now revised the text to specify the reconstruction method. Regarding the axon/dendrite assignments, our dataset was acquired from thalamic explant cultures in which the long, thin neurites extending onto EM grids correspond to axons (≥200 μm away from cell bodies, confirmed by analyzing the polarity of microtubules, as plus end of microtubules are aligned to point to the distal direction of neurite in axons). We have added a clarifying sentence to the manuscript.

Also, please state if montages were collected for all regions of the grids from which high-resolution micrographs acquired for SPA? If not, how many?

Montages were collected whenever spatial context was required, such as for mapping filament trajectories or microtubule lattice states along neurites. In cases where the goal was solely to obtain high-resolution SPA reconstructions of individual filaments or to obtain structures of

macromolecules, montage acquisition was not necessary and therefore was not performed for every SPA region. We have added this information in the methods: "Montages were acquired for regions where spatial context or cellular mapping was required (e.g., for back-plotting microtubule lattice states). For SPA datasets collected for high-resolution structure determination, montage acquisition would not be necessary."

L153: "Notably, we identified a distinct subpopulation of F-actin that was fully decorated on their surface". How? Classification of particles? This is unclear.

The decorated subpopulation was identified through a combination of visual inspection and 2D classification. Fully decorated filaments displayed a noticeably larger apparent diameter and altered helical appearance in the micrographs, allowing them to be recognized before particle extraction. After segmentation, 2D classification in cryoSPARC robustly separated these decorated particles from undecorated F-actin, confirming the presence of a distinct cofilin-bound class. We have revised the text to elaborate this point, alongside the supplementary figure S9 showing the analysis workflow for undecorated and decorated, and detailed Methods.

L160: "Interestingly, these cofilin-bound actin filaments were clustered in discrete regions along axons, where most individual filaments were fully decorated with cofilin." This is interesting, but alas no evidence or figure citation is provided to support this claim.

Our observation was based on individual high-magnification micrographs in which most actin filaments within a given imaged segment appeared fully decorated with cofilin. We have revised the wording to clarify that cofilin-decorated filaments were observed in localized imaged segments, without implying defined discrete axonal domains, and added more examples of micrographs that contain fully-decorated F-actins (new Fig. S6) along the original example figures (Fig. 6).

****Major comment 3:****

The authors also determine the structure of microtubules by SPA, identifying compact (more labile) and stretched (more stable) forms:

L183: "our data provide the first direct in situ evidence for the presence of these distinct lattice conformations within the native neuronal environment". This statement that these structures aareew representative of these proteins in their "native neuronal environment" seems overblown and misleading since the samples to not contain a membrane. The authors then map back into MMM images the location of coordinates giving rise to the stretched versus compact microtubule structure, shown in Fig. 8.

The reviewer is correct, that the absence of the plasma membrane means the preparation is not fully intact, and we have adjusted our wording accordingly. Our intended meaning was that the microtubules analyzed were assembled and maintained in a native cellular environment prior to the ghost preparation, and they kept the same axon trajectory when frozen. They were not reconstituted or polymerized *in vitro*.

L195: "This back-plotting approach allowed the visualization of microtubule conformational states in situ across axonal compartments". How do the authors know these are microtubules within axons versus dendrites? I wonder if the authors can deduce the polarity orientation of the microtubules mapped back into the MMM? If they are axonal, I suppose one would find most microtubules are uniformly oriented ('+' end pointing away from the cell body).

The neurons used in this study were derived from thalamic explants, which project long axons onto EM grids, whereas dendrites remain short, branched, and restricted near the explant body, which is too thick to observe under cryo-EM. We analyzed neurites ≥200 μm away from cell bodies, which

exclusively axons at this distance from explants. We clarified this by adding an explicit explanation in the Result section. Microtubules were traced along these continuous axonal bundles, and continuity of MT bundles from membrane-intact to membrane-free regions provides an additional fiducial confirmation of their axonal identity.

To independently assess neurite identity, we analyzed microtubule polarity orientation as well. Particle orientations derived from our image analysis (frealign) were extracted, and normalized relative to the local neurite axis determined from HMM. Histogram analysis showed most of analyzed microtubule segments aligned towards uniform orientation, as opposed to the 180°, meaning the opposite orientation, consistent with the parallel polarity expected for axonal microtubules. These data are now added in Fig. S7.

This MMM mapping analysis is presented in Fig. 8 showing the distribution of stretched versus compact tubulins in only 10 microtubules in a single ghost neurite. The concern in 'major comment 2' above that the ghost neuron preparation has moved or damaged these cytoskeletal elements might call into question the validity of mapping back structures into the MMM images. Specifically, how can the authors rule out that the ghost-method preparation of removing the membrane such that the cytoplasm mixes with the media surrounding the cells during blotting would not have altered microtubule stability and contributed to the pattern of stretched/compact regions in each microtubule?

We appreciate the reviewer's concern regarding both sampling and potential preparation-induced effects.

Regarding sampling: although Fig. 8 presents representative mapping from a single neurite for clarity, mixed lattice states were observed across multiple neurites, microtubules (7787 microtubules evaluated in total) and independent preparations. In the revised manuscript, we now include additional examples from 45 microtubules (Fig. S7), showing the coexistence of stretched and compact segments is not restricted to a single example.

Regarding potential preparation-induced effects: the measured axial spacings (~81 Å and ~83 Å) correspond closely to the canonical compact and expanded lattice parameters previously reported for microtubules, and we do not observe intermediate states or structural signs of cytoskeletal destabilization such as fragmentation, protofilament peeling, or bundle disorganization. In addition, hypotonic exposure and mechanical rupture occur immediately prior to blotting and plunge freezing, limiting the opportunity for coordinated longitudinal lattice remodeling along pre-assembled microtubules. Generating alternating compact and expanded segments would require organized conformational transitions within the polymer lattice, typically associated with polymerization state, tubulin exchange, or regulated protein interactions.

To further evaluate whether lattice heterogeneity could arise during the Ghost Neuron preparation, we examined power spectra from cryo-ET data of intact neurites in which the plasma membrane remains present. In several cases the strong ~40–42 Å layer line appeared broadened or partially split, consistent with the presence of nearby lattice periodicities within individual microtubules. Representative examples are shown below. Because the signal-to-noise ratio of tomographic data limits robust classification of lattice states, these observations are illustrative rather than quantitative.

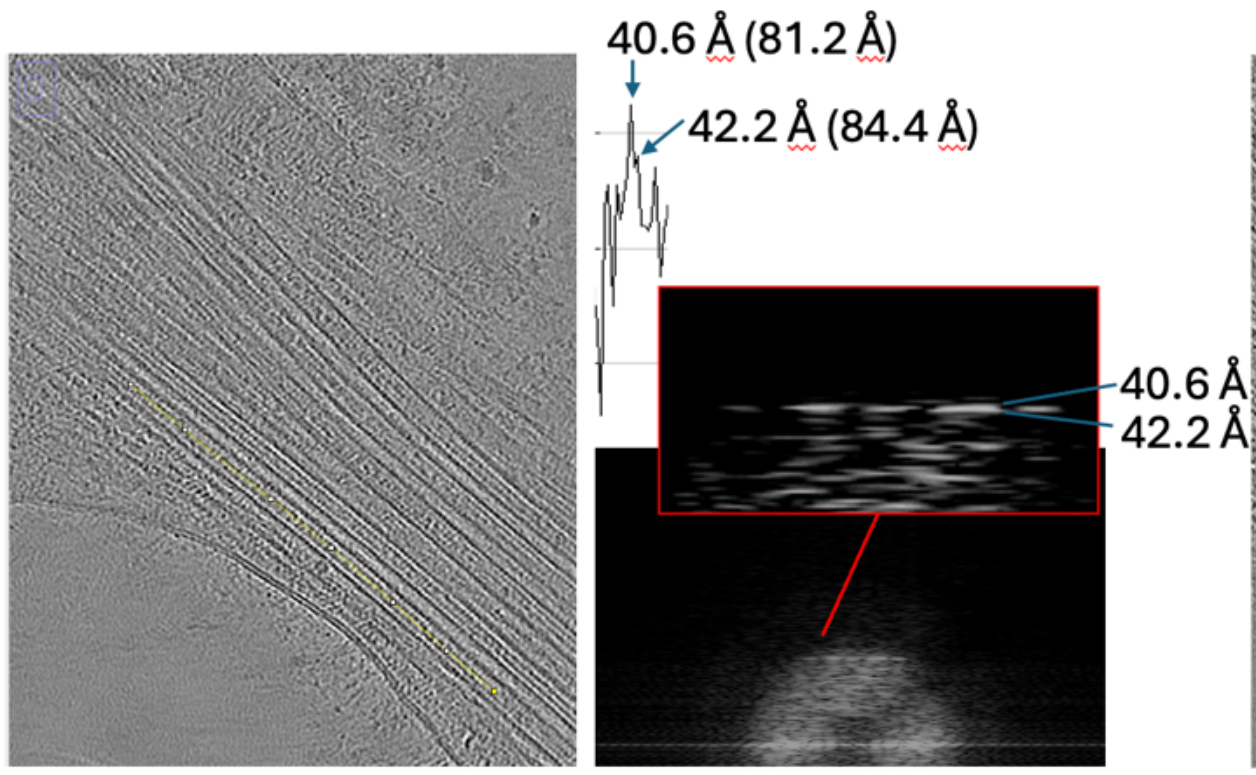

Example 1

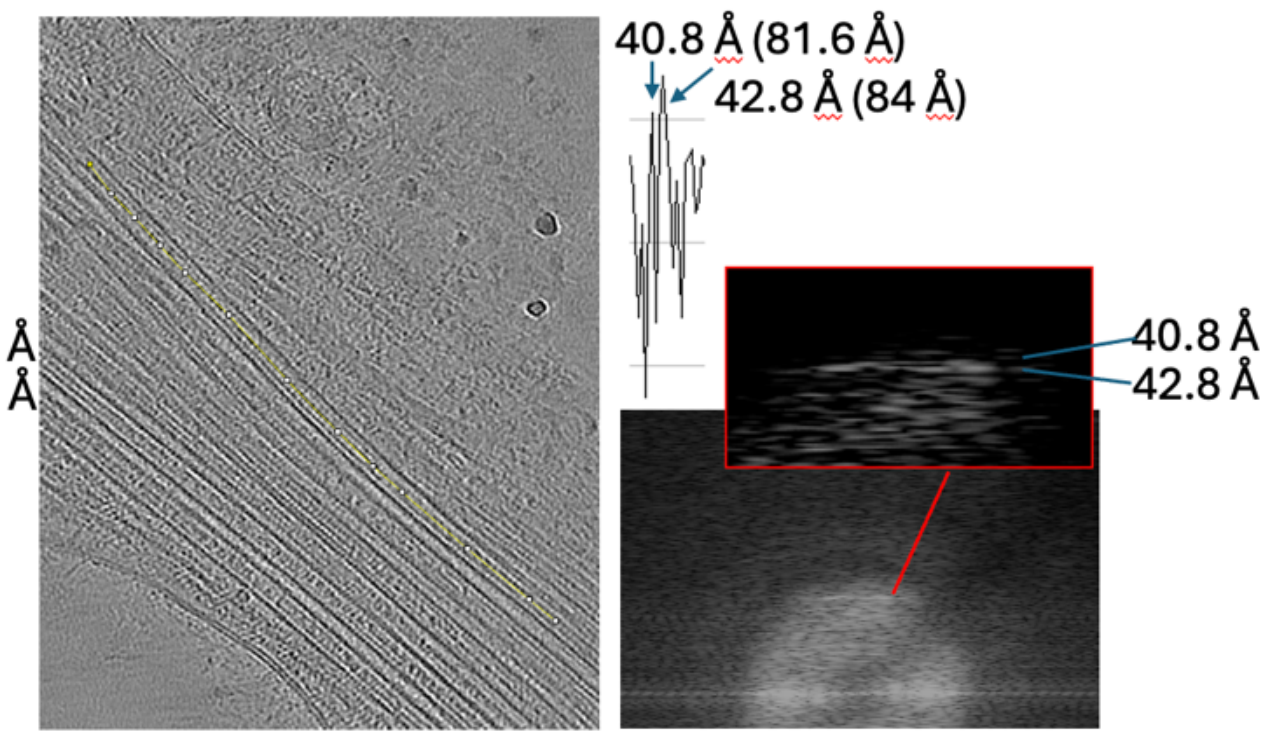

Example 2

A rigorous quantitative evaluation of lattice heterogeneity directly in intact neurons, reaching to publication quality, would require substantially larger tomographic datasets and systematic analyses that extend beyond the scope of the present study, and is a subject of future studies. Together, the reproducibility across neurites, the agreement with established lattice parameters, and the observation of heterogeneous lattice signatures in intact neurites suggest that the mapped stretched and compact segments reflect pre-existing structural states rather than preparation-induced remodeling. We have clarified this interpretation in the revised Discussion.

Additionally, a more extensive quantification, including more examples of this mapping to other MMM of ghost neurites in their dataset may be necessary to convince the reader of the interpretation of the data that: L197: "our back-plotting analysis revealed that stretched lattices, associated with increased microtubule stability, also appeared in mid-sections of axonal microtubules (Fig. 8)."

We added another example of HMM (Fig. S7), showing examples of 45 microtubules with a similar pattern of mixed expanded and compacted lattices coexisting within individual microtubules.

Discussion:

Major comment 4:

L217: "approach preserves the native subcellular architecture". L224: "This method preserves intracellular structures with minimal perturbation and significantly enhances the signal-to-noise ratio, a critical factor for accurate alignment during cryo-EM image processing and 3D reconstruction "This method preserves intracellular structures with minimal perturbation" These statements are inaccurate/overblown. At best microtubule subcellular architecture is partially preserved (not withstanding Fig. 5, see 'major comment 2'), but most other constituents of these subcellular neuronal structures are clearly not preserved.
I am also not convinced that the increased contrast observed in the cryoEM data is simply consequence of removing the membrane. Is it not also likely that removing the membrane could also enable thinner specimens to be prepared? This would be the major contribution to the increased contrast (SNR) evident in the data. Surely removing the membrane and blotting, would permit intracellular macromoleuclar constituents to diffuse outward/flatten out as blotting reduces the thickness of the thin film before plunge freezing?

We agree that the original phrasing overstated the extent of preservation and have revised the manuscript accordingly. We now explicitly state that the method preserves the overall neurite geometry and the alignment of major cytoskeletal elements, rather than claiming preservation of the complete native subcellular architecture. We also clarified in the Limitations section that membrane-associated structures (for example, the membrane periodic skeleton, MPS) are not

retained and that partial redistribution of soluble and small membranous components occurs during preparation. Regarding the origin of increased contrast, we also agree that multiple physical factors contribute. In addition to reduced electron scattering from the removed plasma membrane, local thinning of the specimen and partial redistribution of soluble material during blotting would enhance signal-to-noise. We have revised the source of enhanced contrasts throughout the manuscript.

L268: "Additionally, while the method allows visualization of retained vesicles and membrane-associated components". This statement could be misunderstood. Since the plasma membrane is removed during the ghost neuron preparation, these 'membrane-associated components' can't possibly be visualised can they?

Our original phrasing unintentionally suggested plasma-membrane–associated elements. We have revised the manuscript and removed the corresponding sentence. In addition, throughout the manuscript, we clarified and stressed the limitation of the methods as stated above.

Methods:

L473: "In experiments mimicking cryo-blotting, grids with thalamus explant tissue were initially imaged with MemGlow and DIC for 5 minutes. Subsequently, the grids were treated with hypotonic media and manually back-blotted with the same filter paper used for vitrification for 3-4 seconds before being placed back into the imaging chamber"When mimicking cryo-blotting (related to data in Fig. 5), why were the cells back-blotted with a Leica and not front-and-back blotted with a vitrobot as described for cryoEM grid preparation (as described L508)?

The purpose of the mimic experiment was to test whether membrane rupture occurs upon mechanical blotting following hypotonic treatment, rather than to fully reproduce the vitrification workflow. For this reason, manual backside blotting was used to allow imaging of the same grid region before and after mechanical perturbation. Both Vitrobot preparation (front-and-back blotting) and Leica backside blotting produced comparable membrane disruption under cryogenic conditions. The membrane disruption observed in the mimic experiment also compare well with the cryo-EM observation after Vitrobot preparation.

L587, Fig. 5: Some panel insets lack scale bars and the scale bar in other panels have not been defined in the legend).

The errors are fixed.

Reviewer #3

Bodakuntla et al. present the ghost neuron technique, a preparation method for cryo-electron microscopy imaging of neurons based on removing the plasma membrane. Hypotonic treatment followed by mechanical disruption of the cell improves image contrast and signal-to-noise ratio. Using this approach, the authors obtained structures of axonal F-actin and cofilin-decorated filaments as well as two distinct conformations of microtubule lattices corresponding to stable and dynamic states. Mapping these structural variants onto neuronal geometry revealed coexistence of stable and labile microtubule segments within single axons.

The authors claim that their method removes the plasma membrane while preserving internal organization. In relatively thick specimens, multiple inelastic scattering events dominate image formation, resulting in high background noise and reduced contrast. Because the thin plasma membrane contributes negligibly to total scattering, its removal would not produce any visible difference in contrast. Therefore, the observed increase in contrast must reflect extraction and rearrangement of intracellular material, leading to reduced thickness and macromolecular crowding.

Figure 4B shows spread out microtubules in ghost neuron while in the intact neurite shown in Figure 4A, the microtubules seem much more densely packed.
The application of ghost neuron is mostly restricted to the study of cytoskeleton. And even if it is closer to native state than in vitro reconstitution, the ghost neuron system is far from an intact cell. Although it preserves the intracellular organization better than in vitro reconstitutions, it still represents a system that is far from an intact, living cell.
We agree that ghost neurons do not represent an intact, living cell, and we have revised the manuscript to avoid overstating preservation of native architecture, and our original writing was misleading, or over-claiming. Our method is not intended to recapitulate all aspects of cellular physiology, particularly membrane-dependent processes, it rather provides a structural platform for high resolution cryo-EM, that preserves neurite geometry and major intracellular scaffolds sufficiently to allow molecular states to be mapped back to defined neuronal trajectories. In this study, we showed examples of cytoskeleton at high resolution within axonal geometry. Using these examples, we mean to show that molecular conformations, which is only accessible at the resolution range achievable by SPA, can be integrated within neuronal topology within the same specimen. We therefore view ghost neurons as complementary to both intact-cell methods and *in vitro* reconstitution approaches. Intact-cell methods preserve membrane-dependent processes but are often limited in achievable resolution within densely packed neurites, whereas *in vitro* systems provide atomic detail without spatial context and only contain explicitly added components. The Ghost Neuron preparation therefore bridges molecular resolution and cellular topology within the same specimen. We have revised the manuscript to clarify this position.

More specific points are listed below.
l. 82. The term in situ is inappropriate for the reasons explained above
We have removed the term 'in situ' throughout the manuscript.

l. 100. The authors mention that the morphology of axons remained unaffected after initial exposure to hypotonic media (Figure 2). First, we cannot distinguish dendrites from axons in figure 2. Second, even if the morphology of the plasma membrane at the light microscopy level seems intact, the figure does not prove that the morphology of the plasma membrane, and even more so of the interior of the cell is preserved at a higher resolution than than the images shown in Figure 2.
Regarding axon identification, when we want a clear distinction of axon, we use thalamic explants, in which the long projecting neurites (≥200 μm away from cell bodies) are known to be axons (PMID: 41224993, 35262630, 10328943), and we clarify this in the revised manuscript. For the example in newly added Figure S7, we additionally performed image analysis to determine the polarity of the microtubules relative to the neurite axis. Consistent with axonal organization, microtubules were aligned in a parallel orientation with plus-ends pointing away from cell bodies, providing an independent confirmation of axon identity.
About light microscopy: we agree that light microscopy cannot demonstrate sufficiently whether the ultrastructure is preserved at molecular resolution. The purpose of Figure 2 was meant to assess gross morphological stability during short hypotonic exposure. To assess the effect quantitatively, in the revised manuscript, we added the measurements of the axon swelling per unit length under all displayed conditions. To evaluate physiological tolerance, we additionally assessed axonal growth and neurite density under 160 mOsm conditions for up to 24 h and observed no detectable impairment and the result is added in the revised manuscript. Together with preserved mitochondrial motility, our results indicate that the performed hypotonic treatment does not grossly compromise axonal viability or organization prior to membrane rupture (also see below regarding further discussion about osmolarity).

Ultrastructural preservation was evaluated directly by cryo-EM (Fig. 4, Fig. S2), which shows, although plasma membrane is no longer present, retention of bundled microtubule organization and characteristic axonal mitochondrial morphology under the selected conditions. We have clarified this distinction in the revised manuscript.

l. 107. The authors observed that reducing osmolarity up to 50% did not significantly influence mitochondrial motility. Nonetheless, the physiology of the neurons is likely strongly affected by a drop of 50% in osmolarity, which is used later to prepare ghost neurons. This might result in very rapid structural changes at the molecular level.

We agree that a reduction in osmolarity could potentially affect neuronal physiology. However, in our experiments we did not observe evidence that the 160 mOsm condition used for Ghost Neuron preparation caused significant cellular perturbation. To minimize potential stress, hypotonic exposure was limited to 5 minutes prior to blotting and vitrification. Within this interval, we did not observe gross morphological changes (also see above), axonal breakage, or impairment of mitochondrial motility at 160 mOsm. These observations are consistent with previous reports showing that neurons tolerate moderate hypotonic conditions (PMID: 1907290), which we now cite in the revised manuscript.

To further assess potential cellular stress, we performed additional experiments measuring neurite density and axonal extension following prolonged exposure (24 h) to 160 mOsm medium. We did not detect reductions in neurite density or overt signs of neuronal death under these conditions. During the experiment axons continued to extend and remained active. Together with the mitochondrial motility measurements and prior literature, these results indicate that neurons tolerate the 160 mOsm condition without detectable cytotoxic effects. Importantly, membrane rupture itself occurs only during mechanical blotting immediately prior to plunge freezing. Thus, hypotonic incubation is transient and precedes vitrification by only seconds. We have added these additional control experiments and clarifications to the revised manuscript.

l. 115. What does gentle blotting mean?

We revised the terminology to simply 'blotting,'. The detail of the blotting procedure is described in Methods.

Mitochondria in axons and dendrites are quite elongated. Yet, in the ghost neurons, mitochondria look mostly round. This indicates that they have undergone significant morphological changes.

We would like to clarify that mitochondria are not uniformly elongated across neuronal compartments. In axons, mitochondria are typically short and ovoid, or appear as a series of discrete "beads," while more elongated morphologies are characteristic of dendrites (PMID: 30479337, 35262630). In our datasets, mitochondria in ghost neurons show similar ovoid shapes and remain closely associated with axonal microtubule bundles, with no evidence of swelling, vacuolization, or fragmentation (Fig. 4B, 8 S5B, also see Movie 2). We have added a clarification in the text.

The authors claim resolutions ranging from 3.26 to 5.32 Å for their single particle analysis reconstructions. The cryo-EM maps were not provided and a visual inpection cannot confirm these values. The PDB EM validation reports do not at all support the resolutions claimed by the authors. Indeed, instead of 3.26 Å, the report gives a resolution of 7.26 Å. For the map claimed at 3.66 Å, the report finds 4.45 Å. For the one claimed at 3.96 Å, the report says 6.18 Å. And for the map claimed at 5.32 Å, a resolution of 9.04 Å is calculated by the validation report. It seems that the masks used by the authors for resolution estimate are too tight and lead to significant resolution overestimate.

The lower unmasked FSC values reported in the validation report reflect differences in FSC calculation procedures and the map volumes included in the analysis. For both microtubule and actin reconstructions, refinement and resolution estimation were performed using gold-standard FSC with soft masks focused on the structurally ordered regions, consistent with standard practice in filament refinement and local reconstruction. In the deposition, we provide the full, unmasked maps and corresponding half-maps, as required for validation. The EMDB validation pipeline calculates FSC over the entire unmasked volume; for elongated filamentous assemblies reconstructed in relatively large boxes, this incorporates surrounding background as well as peripheral or less coherently aligned regions, yielding a lower resolution estimate than the masked FSC focused on the ordered filament core. This effect is particularly pronounced for microtubule lattice states, where high-resolution signal is concentrated within the aligned protofilament region while other regions contribute more strongly to the unmasked FSC as background signal.
Consistent with this notion, we tested that cropping of the maps to remove excess empty volume without applying any mask improved agreement between masked and unmasked FSC estimates, indicating that the discrepancy is driven largely by background contribution than the absence of high-resolution signal in the ordered filament portion. However, we retained the full map volumes (and edge of the map removed for the case of actin) in the deposition to preserve structural context and avoid introducing biased interpretation. This behavior is also consistent with CryoSPARC processing discussions, which note that unfiltered half-maps can appear substantially noisier than filtered reconstructions; in large reconstruction volumes, this noise can contribute to lower unmasked FSC estimates relative to the ordered region, with the magnitude of the discrepancy varying between datasets (https://discuss.cryosparc.com/t/noisy-halfmaps-after-nu-refinement/24708/11). In our case, importantly, the refined maps display structural features consistent with the reported resolution range, including clear side-chain densities and well-resolved secondary structure in actin, as well as separation of α- and β-tubulin features such as the S9–S10 loops (a feature typically resolved at beyond ~4 Å) in microtubules. Snapshots of the densities are now shown in Fig. S8, and we also improved our maps, also added model building on the structures (Microtubules and F-actin). Together, these features support the reported resolution estimates

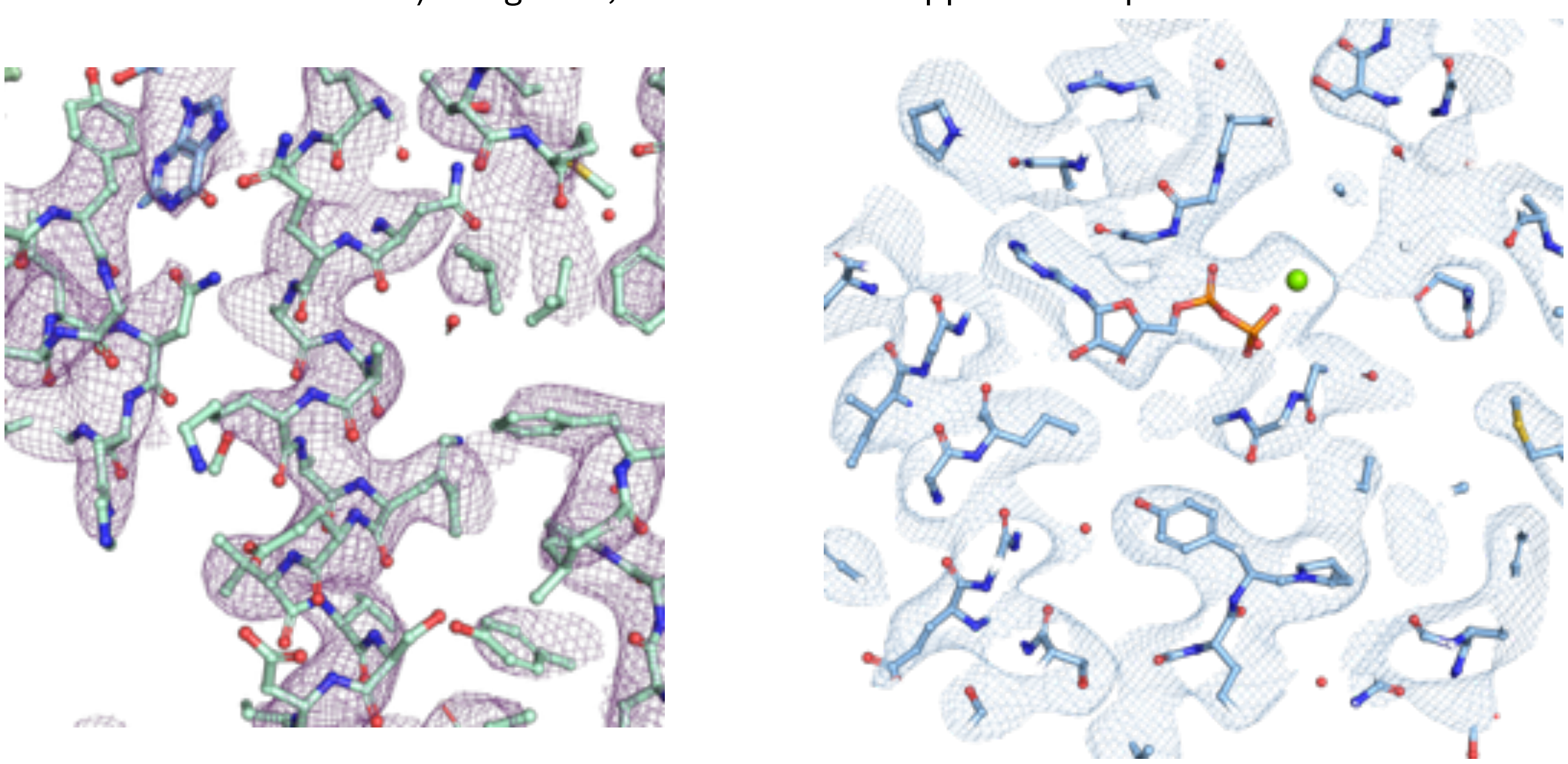

despite the lower unmasked FSC values.

Representative density of the cryo-EM reconstruction of the stretched microtubule lattice (left: 3.66 Å) and actin (right: 3.2 Å) showing the quality of the maps used for model building. The maps are shown together with the refined atomic models. The levels of detail visible in the maps are consistent with resolutions of ~3.2-3.6 Å.

l. 197. The distribution of the two types of microtubule lattice is reported. Yet, it is not clear how robust the detection of the type is. This should be quantified. The distribution of likelihood of each particular microtubule segment to be of one or the other type should be shown. More over, the claim that the found distribution reflects the situation in situ should be backed by distribution obtained in cryo-ET data of intact neurons.

To evaluate the robustness of lattice-state classification, we repeated 3D classification six times using independent random initializations. All runs produced similar class distributions (~60% stretched, ~40% compact). For the final dataset (30,423 compact segments), 94.8–96.6% of particles were assigned to the same class in each of the other five runs, indicating high reproducibility of classification. In addition, per-particle posterior probabilities showed the majority of segments assigned with high confidence (posterior values close to 0 or 1), consistent with well-separated structural states. Effective Sample Size (ESS) values remained high throughout classification, further supporting stable and non-degenerate class separation. These analyses are now included in Supplementary Fig. S8.

The reviewer also suggested comparing these results with observations from intact neurons using cryo-ET. While the signal-to-noise ratio of tomographic data does not permit the same level of structural classification as SPA analysis, we examined power spectra from individual microtubule segments in cryo-ET data of intact neurites covered with plasma membrane. In several cases, the layer-line corresponding to the tubulin repeat showed broadened (example 1) or partially split peaks (example 2) indicating the coexistence of two nearby periodicities, as shown below.

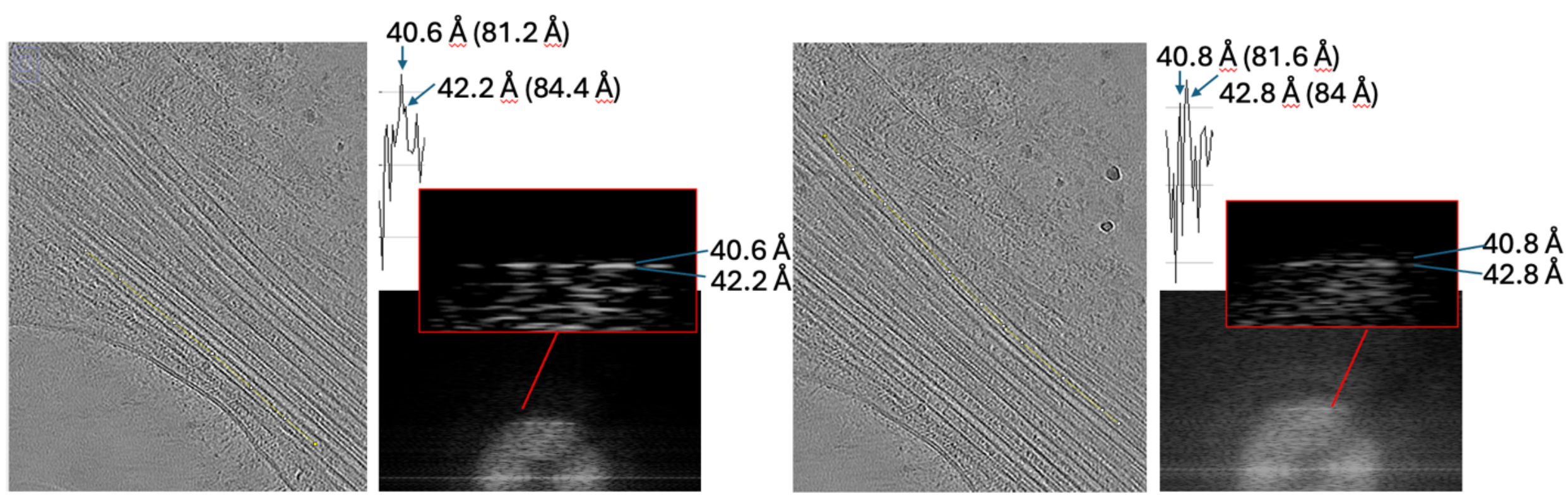

Example 1 Example 2

Because the signal in these spectra is inherently limited by tomographic SNR and segment length, a rigorous statistical analysis would require substantially larger datasets and extended analysis beyond the scope of the present study. We therefore consider these observations preliminary and have not included them in the manuscript. Nevertheless, they are consistent with the lattice heterogeneity revealed by the higher-resolution SPA analysis.

l.220. Neurites, which are the target cell regions of the ghost neuron technique, are actually accessible by cryo-ET without the need of cryo-FIB milling. Thus the advantage of the technique is not obvious.

The neurites are geometrically thin enough to be imaged by cryo-electron tomography without cryo-FIB milling, and several studies have successfully visualized neuronal processes by cryo-ET. However, while neurites are accessible in terms of thickness, their dense cytoplasm and abundance of membrane-bound organelles substantially limit signal-to-noise ratio and, consequently, the

achievable molecular resolution (in practice, 10-20 Å resolution), which would not permit direct observation of conformational states, drug engagement, etc. The primary advantage of the Ghost Neuron approach is therefore not geometric accessibility, but contrast enhancement facilitating high-resolution single-particle analysis within neurite trajectories. This allowed us to resolve microtubule lattices at 3.6–4.0 Å, distinguish compact and stretched conformations, and F-actin with decorations. Such resolution is currently beyond what is typically achieved in intact neurites by cryo-ET. In addition, the approach provides a complementary dispersed particle fraction amenable to SPA. We have clarified this distinction in the revised manuscript.

It is not indicated how long the cells are exposed to low osmolarity before plunge freezing.
The exposure duration is 5 min, which is specified in the Results section. For clarity, we also added this information in the Methods.

REVIEWER COMMENTS

Reviewer #1 (Remarks to the Author):

In their revised manuscript, Bodakuntla et al. have addressed some of the key concerns raised in the reviews. In particular, my points about representation of previous works, better delineation fo the specific advantages and limitations of their ghost neurons procedure, and discussion of the procedure effect on the conformational state of microtubules have been thoroughly addressed. I have a couple of remaining points, mostly on the microtubule conformation results and interpretations, that can be addressed as text revisions before recommending acceptance for publication.

1. It would be better to unify the nomenclature of the tubulin conformation and use "expanded" throughout (as used in most of the literature), rather than "stretched".

We thank the reviewer for this suggestion. We have replaced "stretched" with "expanded" throughout the manuscript, figures, and legends.

2. The link between expanded/GTP/stable and compacted/GDP/labile (or dynamic) should still be toned down as it reflects a vision of microtubule structure rooted in classic articles on plus ends polymerization, rather than the more nuanced view of the states within the lattice away from plus ends, which are what the authors focus on.

We have revised the text to tone down this interpretation. We now describe the expanded/GTP-like and compact/GDP associations as a classical view most applicable to newly polymerized plus ends, and we clarify that this relationship is more complex within mature lattice interiors, particularly in axons. We also removed language that directly equated expanded and compact lattice states with stable and labile (dynamic) microtubule behavior. The revised manuscript instead interprets the expanded and compact segments as local structural states whose molecular determinants and functional consequences remain to be established.

3. The revised explanation and data regarding the "resolution" of the conformation assignment down to a single monomer is convincing. Still for a non-specialist it is a bit surprising that the difference in length between expanded and compacted conformations (1.9 Å) is lower than the resolution of the EM data – it might be useful to explain how that's possible.

We agree this distinction is worth clarifying for a general readership. Map resolution is estimated by FSC-based and describes the smallest independently resolvable density feature in a given reconstruction, whereas the inter-dimer spacing reported here is a relative distance measured from atomic models modeled into two independently classified and refined reconstructions, each representing an average over tens of thousands of individually aligned segments (48,459 expanded, 30,423 compact). Thus, 1.9 Å difference is not inferred from resolving 2 isolated density points separated by less than the nominal

map resolution, but from a systematic shift in the axial repeat measured across many equivalent tubulin heterodimers. Because the measurement is based on repeated structural elements averaged over many particles, the precision of the lattice spacing estimate can exceed the nominal resolution of an individual map feature. We have added this clarification to the Methods, alongside the model building and refinement statistics for the two lattice states.

4. Regarding the conformation classification, it's not entirely clear how the authors validate that there are only two states (expanded or compacted with no intermediates). What about the unlabeled monomers in Fig. 7, 8 and S7? Do they represent segments where assignment fails, or states that are not one or the other? Overall in the dataset, what is the % of imaged microtubule length that is 1) assigned to expanded conformation 2) assigned to compacted conformation 3) not assigned to one or the other?

We agree that this point required clarification. We have revised the manuscript to state more explicitly that our classification resolves two major lattice states, expanded and compact, but does not exclude rare or transient intermediate conformations below the detection threshold of the present analysis. The two classes correspond to well-established axial lattice spacings from prior structural studies, and in our dataset they were robustly separated: per-particle posterior probabilities clustered near 0 or 1 rather than broadly populating intermediate values (Fig. S8B), and class assignments were highly reproducible across six independent classification runs, with 94.8–96.6% consistency (Fig. S8E).
We also clarified the meaning of the unlabeled regions in Figs. 7, 8, and S7. These regions do not mean a third or intermediate lattice state. They correspond to segments excluded during upstream particle cleaning before the lattice-state classification, or not traced by filament tracing function. Of the 158,108 initially picked segments along 1,296 µm of analyzed microtubules, 78,882 segments passed cleaning, then 48,459 were assigned to the expanded conformation and 30,423 to the compact conformation (corresponding to 61.4% expanded and 38.6% compact of the cleaned particles). Relative to the initially picked segments, this corresponds to 30.6% expanded, 19.2% compact, and 50.1% excluded during 2D classification. We have added this quantitative breakdown to the Methods and clarified in the figure legends that unlabeled positions reflect upstream processing exclusions rather than unresolved intermediate lattice states.

5. Regarding the validation of the conformation classification, it would be useful to show a plus end from the existing data, as plus ends are known to have an expanded conformation cap followed by a compacted lattice.

We thank the reviewer for this helpful suggestion. A representative microtubule end is rare to find, but an example can be found in the mapped dataset shown in Fig. S7C. We have now revised the Fig. S7 legend to point out the leftmost inset, where a microtubule plus end shows an expanded lattice patch at the tip followed by compact lattice assignments toward the shaft. Because our acquisition strategy was designed to map lattice states along axonal microtubule shafts and did not specifically target the microtubule ends, plus ends are infrequently captured

in these montages. We therefore would like to present this example as an internal consistency check instead of a systematic analysis of plus-end cap structure.

6. A recent article from the Roll-Mecak lab (Zehr et al. Nat Struct Mol Biol 2026) has examined the tubulin compaction state along the axons of neurons, proposing that all monomers are found in the expanded state. It would be good if the authors could mention this and explain why they obtain a different result (mix of compacted and expanded states).

We thank the reviewer for bringing this recent study. We have now added Zehr et al. (2026) to the revised manuscript. This study reports that axonal microtubules in human iPSC-derived neurons are predominantly expanded despite being GDP-bound. This is consistent with our finding that the expanded state predominates in axonal microtubules (61% of segments) and supports the general uncoupling of nucleotide state from lattice conformation. The compacted population we classified out may reflect differences in species, neuronal origin, developmental stage, and sampled axonal region, or analysis strategy, including our focus on resolving local heterogeneity within individual microtubules.

Reviewer #2 (Remarks to the Author):

This manuscript is much improved. The authors have answered my comments with major revisions of the text and figures, and included new cryoCLEM data. Together, these address the major concerns I had. I congratulate the authors on an excellent study and recommend this manuscript for publication with minor changes (not requiring further peer-review).

Minor changes:

Title: "Ghost neuron unlocks in situ high-resolution structural mapping of intracellular architecture in neurons". Since the ghost neuron preparation is, as the authors acknowledge, not exactly 'in situ' and this terminology has been taken out of the main text in the revised manuscript, it might be necessary to change the title also. The current title also has the 'neuron' noun twice, which seems a bit clunky. Could go with 'Ghost neuron preparation unlocks high-resolution structures with cellular context'.
We thank the reviewer for this suggestion. Addressing the concerns, we adapted the title to 'Ghost Neuron preparation unlocks high-resolution structural mapping of intracellular architecture in axons'.

L160: "suggesting that neurons can withstand short-term hypotonic". This needs to be slightly more cautiously written because though neuronal swellings may not arise and axonal transport was unperturbed, electrophysiological functions are almost certainly impaired (even within a few seconds).
We thank the reviewer for this suggestion. We now modified the text to limit the conclusion to the assays performed, namely, that short-term hypotonic exposure did not cause gross axonal disruption or detectable impairment of mitochondrial transport under the conditions tested.

L328: “by conventional cryo-ET or extraction-based methods”. Using the term ‘conventional cryo-ET’ here and elsewhere in the manuscript is ambiguous and annoy the cryo-ET community. I strongly recommend being more specific eg. rephrase to “by cryoET of intact cells”.
We thank the reviewer for this suggestion and agree the term "conventional" is inappropriate. We have replaced it to “cryo-ET of intact cells” and “cryo-ET of intact neurites”.

There may be the odd minor grammatical error – I haven’t combed the text this closely – please check.
We have now double checked all the manuscript for grammatical errors.

Reviewer #3 (Remarks to the Author):

The authors have addressed satisfactorily all my concerns. The manuscript has been significantly improved and should be published. I noticed two minor points: the newly added author, S. Glauser, is not listed in the author contribution section; in table S1, two maps have the same code (EMD-76718).
We thank the reviewer for pointing this out. We have now added the author contribution. ‘S.G. synthesized and provided the fluorescent Epothilone-B probe used for membrane integrity assays.’ We have fixed the table S1.
